# Perioperative corticosteroids and pancreatic surgery outcomes: a systematic review and meta-analysis combining human expertise and AI support (ChatGPT)

**DOI:** 10.1007/s13304-026-02750-3

**Published:** 2026-07-08

**Authors:** Marcello Di Martino, Andrea Saibanti, Gianluca Cassese, Mariantonietta Alagia, Fabrizio Panaro, Matteo Donadon

**Affiliations:** 1 Department of Health Sciences, University of Piemonte Orientale, Novara, Italy; 2 Division of Surgery, Maggiore Della Carità University Hospital, Corso Mazzini 18, 28100 Novara, Italy; 3 Division of General Surgical Oncology, Robotic and HPB Surgery, SS. Antonio E Biagio E Cesare, Arrigo University Hospital, Alessandria, Italy

**Keywords:** Pancreatectomy, Pancreatic adenocarcinoma, Pancreatitis, Post-operative pancreatic fistula, Corticosteroids

## Abstract

Pancreatic surgery is associated with high postoperative morbidity. The role of corticosteroids (CCS) in pancreatic surgery remains uncertain. Systematic review and meta-analysis conducted in accordance with PRISMA guidelines. Eligible studies included patients undergoing pancreatic resection with perioperative CCS administration. Primary outcomes were mortality, morbidity and surgical site infections (SSI). Data extraction was cross-validated with artificial intelligence (ChatGPT). Nine studies (1913 patients), including five RCTs (554 patients), were included. CCS regimens consisted of hydrocortisone or dexamethasone, with or without postoperative continuation. The analysis showed no significant differences in mortality or major complications. Data from RCTs showed that CCS reduced SSI (OR 0.53, 95%CI 0.32–0.91, *p* = 0.02). Also, among patients undergoing pancreatoduodenectomy, CCS reduced morbidity (OR 0.61, 95%CI 0.39–0.98), SSI (OR 0.57, 95%CI 0.40–0.82), and shortened hospital stay (MD –1.57 days, *p* = 0.04). Agreement between manual and AI-assisted data extraction was high (r = 0.97–1.00), but substantial discrepancies were found. Perioperative CCS appear safe in pancreatic surgery and may reduce morbidity and SSI. Large multicentre RCTs are needed to define optimal regimens and identify patients most likely to benefit. AI-assisted review may complement traditional approaches but still require further refinement before they can be considered as reliable as human effort.

**Supplementary Information:**

The online version contains supplementary material available at https://doi.org/10.1007/s13304-026-02750-3.

## Introduction

Pancreatic surgery remains associated with considerable postoperative morbidity, most notably the development of clinically relevant postoperative pancreatic fistula (CR-POPF) [1] and infectious complications. Despite significant advances in surgical techniques, perioperative care, and risk stratification model, postoperative complications remain high [2–5].

A variety of mitigation strategies have been explored, including surgical technique modifications, pharmacological interventions and perioperative nutrition [6–8]. More recently, postoperative inflammation of the pancreatic remnant has been increasingly recognized as a complication after partial pancreatic resections [9–11], with evidence suggesting that postoperative acute pancreatitis (PPAP), defined as postoperative serum hyperamylasemia combined with clinically and radiologically relevant features, may play a central role in the development of major postoperative complications [2, 12].

Corticosteroids (CCS) have attracted growing interest due to their potent anti-inflammatory effects. Their use in other fields of major surgery, such as hepatic, and colorectal resections, has shown potential in reducing systemic inflammatory response syndrome (SIRS) and complications related to excessive inflammation [13–18]. However, the role of CCS in pancreatic surgery remains controversial and based on relatively low quality evidence [19]. While chronic use of CCS is known to impair wound healing and increase infection risk [20], short-term perioperative administration may offer clinical benefit, particularly in modulating inflammatory responses and mitigating POPF development [21]. Previous systematic reviews have examined this topic but were affected by methodological limitations and reported inconsistent results [19–21]. Current clinical practice regarding perioperative corticosteroid administration in pancreatic surgery remains highly heterogeneous, with some centres adopting routine use and others avoiding it altogether, reflecting the absence of clear evidence-based recommendations.

This review aims to synthesize the current evidence regarding the role of perioperative CCS in pancreatic resections. Specifically, the present study aimed to: (a) provide clinicians with a practical overview of the timing, dosage, and rationale for steroid administration in the perioperative setting; (b) evaluate the impact of CCS on postoperative outcomes, including morbidity and mortality, with particular attention to CR-POPF and surgical site infections (SSI), which remain the Achilles’ heel of pancreatic surgery; (c) address the absence of a clear standard of care, as no international guideline currently recommends for or against the routine use of perioperative CCS in this context [22, 23]. This updated review is the product of a joint effort combining human clinical expertise and artificial intelligence (AI), ChatGPT, with the goal of assess the reliability of AI-tools and the possibility of enhancing the efficiency of evidence synthesis in surgical research.

## Methods

### Eligibility criteria

The review included studies that enrolled adult patients submitted to pancreatic resection and administration of CCS during the perioperative period.

### Search strategy

The search was undertaken according to the PRISMA guidelines [24]. Two researchers systematically searched Medline, Web of Science and the Cochrane Library for reports published before the 8th of March 2025, using a combined text and MeSH search strategy. The search terms for the literature review were divided into two groups. The first group contained the keywords glucocorticoids, hydrocortisone and dexamethasone; the second group contained the keywords pancreatic surgery, pancreaticoduodenectomy and distal pancreatectomy. The search terms were structured by combining one word from each group in such a way that all possible combinations were employed. References from relevant papers were also included in order to constitute the initial pool of articles.

### Study selection

Studies that met the following criteria were included: (a) participants aged 18 years or older; (b) observational prospective and retrospective, or randomised controlled design; (c) patients submitted to pancreatic resection and administration of CCS during the perioperative period. Experimental studies on animal models, case reports, short case series with fewer than 10 patients, reviews, editorials and comments were also excluded. When duplicate reports from the same study were identified, only the most recent publication or the one with the longest follow-up period was included. The full text of each article was assessed if it could not be excluded by the initial review. Post-operative outcomes were extrapolated from the figure and tables when they were not reported.

### Data extraction

Two researchers (AS and MA) assessed the abstracts of the selected studies to determine their eligibility. Full articles were selected for further assessment. The extracted data included country of study; design; number of participants; age; surgical procedure performed; perioperative mortality and morbidities. The extracted data included country of study; study design; number of participants; patient age; surgical procedure performed; perioperative outcomes including reported mortality; major and minor morbidity according to Clavien-Dindo classification [25]; CR-POPF (1) when specifically reported, or postoperative pancreatic fistula (POPF), including both biochemical leaks and CR-POPF, when the type of pancreatic fistula was not clearly specified; bile leak [26]; surgical site infection (SSI); delayed gastric emptying (DGE) [27]; reintervention; readmission; and length of hospital stay (LOS). Disagreements over data extraction were resolved by consensus between the two authors and with the support of a third reviewer (MDM).

### Evaluation of studies and statistical analysis

Two researchers (MDM and GC) independently evaluated the included studies for quality assessment according to either the Newcastle–Ottawa Scale [28], in case of observational studies, or the Cochrane Risk of Bias Tool, in case of experimental studies [29]. The data were analysed using the statistical software Review Manager 5.4 and presented as medians and proportions along with a corresponding minimum–maximum range. Differences in dichotomous variables were calculated using an odds ratio (OR) and respective 95% confidence intervals (CI); for continuous variables, the mean difference (MD) was calculated with a 95%CI. A random-effects model was used to take into account the heterogeneity of the estimates. Values were considered statistically significant when P was less than 0.05. The overlapping of CI was used to visually assess the heterogeneity. Heterogeneity was statistically explored with the Chi-square test, with significance set to a p-value of 0.10, and the quantity of heterogeneity was measured with the I^2^ statistic.

Furthermore, the ChatGPT artificial intelligence system (OpenAI, San Francisco, CA, USA) was employed to perform an independent data extraction from the included studies. This step was conceived as an exploratory, proof-of-concept analysis aimed at assessing the potential role of AI in systematic reviews. Specifically, the comparison sought to evaluate the precision of AI outputs, the degree of discrepancy between human- and AI-based data extraction, and the independence and reliability of AI in interpreting scientific literature. A correlation analysis was therefore conducted, with identification of discrepancies between AI-driven and human-performed data extraction.

## Results

### Literature review

**Fig. 1:**
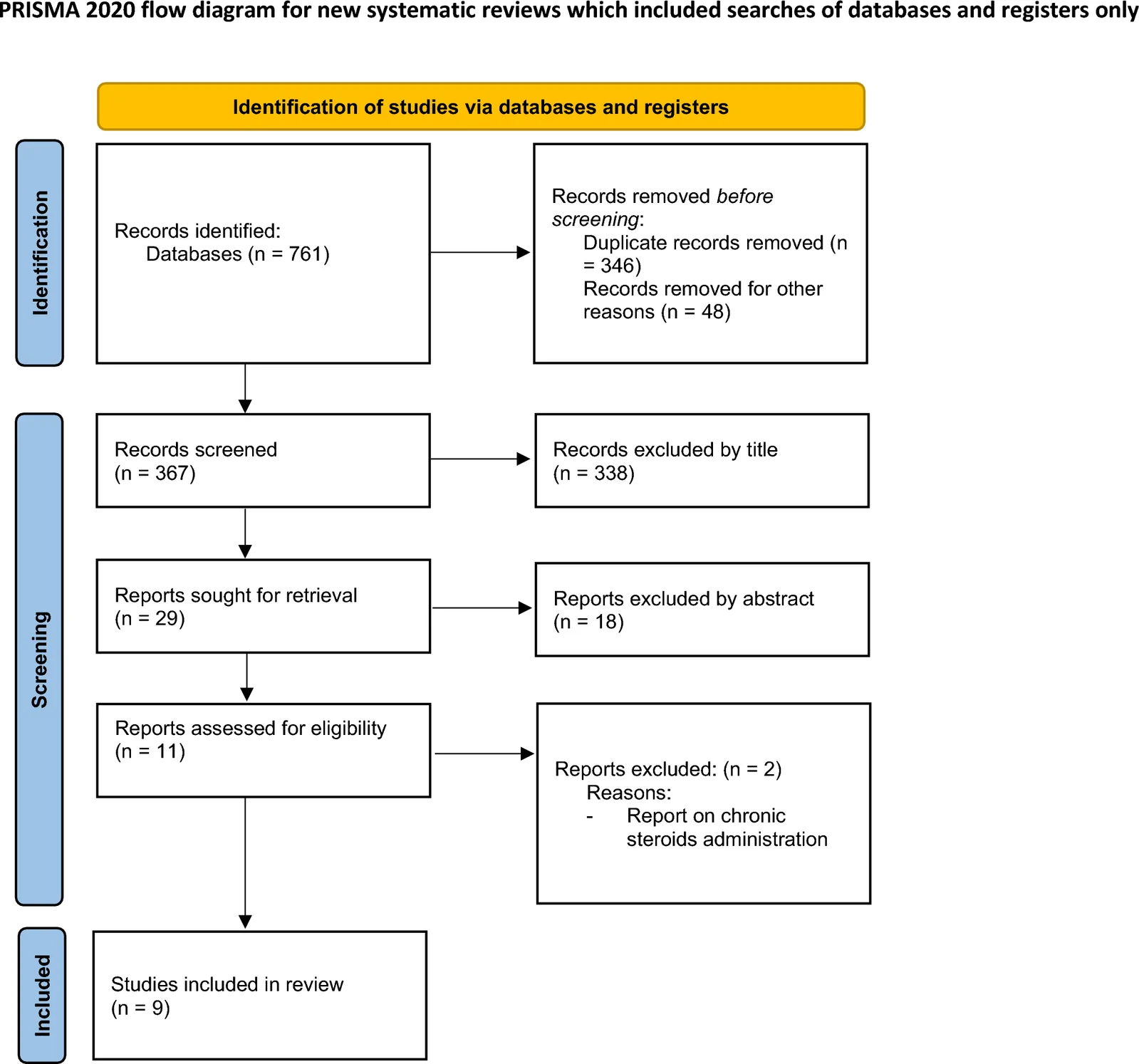
PRISMA flow chart

The literature search yielded 761 records, 750 of which were excluded due to study characteristics or methodology. The full-text articles of 11 papers were assessed for eligibility [30–40]. Finally, 9 studies [30, 32, 33, 35–40] (1913 patients) were ultimately included in the analysis (Fig. 1).

### Study and patients’ characteristics

Five randomised clinical trial were found [30, 33, 35, 36, 39] (554 patients) (Table 1), and none of the included papers was considered as having a high risk of bias (Supplementary Tables 1 and 2). Four manuscripts assessed exclusively patients submitted to pancreatoduodenectomy (PD) [33, 35, 36, 38] (1076 patients), one focused on patients submitted to distal pancreatectomy (PD) [30] and the remaining evaluated patients submitted to various type of pancreatic resections [32, 37, 39, 40]. Median age ranged from 55 to 67.5 years. Other patient and disease characteristics such as the administration of neoadjuvant chemotherapy, the number of patients submitted to venous resection or the number of patients with higher risk for POPF such as a soft pancreas or a small pancreatic duct are shown in Table 1.

**Table 1:** Characteristics of included studies: study design, patient demographics, surgical procedures, and baseline characteristics

Study ID	Country	RCT	Retrospective or prospective	PSM	Sample size (n)	Age (median)	Sex (n. females)	Surgical procedure	Neoadjuvant therapy (n)	Venous reconstruction (n)	Soft pancreas (n)	Small pancreatic duct (< 3 mm) (n)
Antila [30]	Finland	1	Prospective	0	31	67.5	20	DP	–	–	31	29
Call [32]	USA	0	Retrospective	1	144	66	68	Various	40	–	–	–
Chen [33]	China	1	Prospective	0	265	62	105	PD	8	30	116	–
Kant [35]	India	1	Prospective	0	70	55	23	PD	–	–	57	–
Laaninen [36]	Finland	1	Prospective	0	62	65	23	PD	–	–	–	–
Newhook [37]	USA	0	Retrospective	0	373	65	163	Various	281	–	–	–
Sandini [38]	USA	0	Prospective	0	679	66	357	PD	202	–	–	–
Tarvainen [39]	Finland	1	Prospective	0	126	65.5	66	Various	4	9	–	–
Zhang [40]	China	0	Retrospective	1	128	63.5	54	Various	0	–	–	–

*RCT* Randomised clinical trial, *PSM* Propensity score matching, *DP* Distal pancreatectomy, *PD* Pancreatoduodenectomy

### Timing and dosage of steroid administered

CCS were administered either intraoperatively alone or intraoperatively with continued dosing in the early postoperative period. Hydrocortisone was generally given as a fixed regimen of 100 mg intraoperatively followed by repeated doses every 8 h until the second postoperative day (total of 8–9 doses) [30, 35, 36, 39]. Dexamethasone was mostly administered as a single intraoperative dose, with variability in timing and dosage: fixed weight-based regimens (0.2 mg/kg) or flat dosing (1–10 mg) [32, 33, 37, 38]. (Table 2).

**Table 2:** Corticosteroid regimens and outcomes: timing, dosage, and type of corticosteroid administered; reported clinical outcomes

Study ID	Sample size (n)	Intervention	Notes on CCS administration	Notes on reported clinical outcomes
Antila [30]	31	Hydrocortisone	100 mg hydrocortisone intraoperatively, then every 8 h until the second postoperative day (total of 9 doses)	Hydrocortisone reduced overall major complications and clinically significant POPF
Call [32]	144	Dexamethasone	Between 1 and 10 mg dexamethasone intraoperatively	Dexamethasone improved overall survival
Chen [33]	265	Dexamethasone	0.2 mg/kg dexamethasone intraoperatively	Dexamethasone did not significantly reduce postoperative complications
Kant [35]	70	Hydrocortisone	100 mg hydrocortisone intraoperatively, then every 8 h until the second postoperative day (total of 8 doses)	Hydrocortisone reduced overall complications
Laaninen [36]	62	Hydrocortisone	100 mg hydrocortisone intraoperatively. In high-risk patients, treatment continued with 100 mg every 8 h until the second postoperative day	Hydrocortisone reduced overall complications
Newhook [37]	373	Dexamethasone	dexamethasone intraoperatively at the discretion of the anaesthesiologist	Dexamethasone did not significantly reduce postoperative complications
Sandini [38]	679	Dexamethasone	Between 4 and 10 mg of dexamethasone intraoperatively	Dexamethasone reduced infectious complications and improved overall survival
Tarvainen [39]	126	Hydrocortisone	100 mg hydrocortisone intraoperatively, then every 8 h until the second postoperative day 2 (9 doses)	Hydrocortisone did not significantly reduce postoperative complications
Zhang [40]	128	Glucocorticoids	Either dexamethasone (5 or 10 mg) or methylprednisolone (40–60–80 mg) Postoperatively, either 5 or 10 mg of dexamethasone was used	Glucocorticoids improved recurrence-free survival

*CCS* Corticosteroids

### Clinical outcomes

Overall, six out of the nine included studies reported beneficial effects of CCS use [30, 32, 35, 36, 38, 40]. CCS were associated with reduced post-operative complications in four studies [30, 35, 36, 38], and improved survival in three studies [32, 38, 40]. Conversely, three large studies found no significant reduction in postoperative complications with CCS administration [33, 37, 39] (Table 2).

The meta-analysis showed no significant difference in mortality and morbidity, including POPF, bile leak, delayed gastric emptying (DGE). A trend toward reduced SSI (OR 0.72, 95% CI 0.51–1.03, *p* = 0.07; I^2^ = 19%) was observed, although these did not reach statistical significance. CCS did not significantly influence reintervention or readmission rates, or length of hospital stay (Table 3). None of the included studies reported an increased incidence of post-pancreatectomy diabetes or steroid-related adverse events or in the CCS group.

**Table 3:** Summary of meta-analysis comparing perioperative corticosteroid versus non-corticosteroid groups for mortality, morbidity, and other clinical outcomes

Outcomes	Studies	Patients (CCS vs non-CCS)	OR / MD (95% CI)	p-value	I^2^ (%)
CCS vs Non-CCS Outcomes: global data					
Mortality	6	458 vs 938	1.05 (0.42–2.60)	0.92	0
Major morbidity	7	617 vs 1024	0.91 (0.63–1.30)	0.59	22
Minor morbidity	5	366 vs 840	0.76 (0.53–1.10)	0.15	23
Global morbidity	7	617 vs 1024	0.75 (0.48–1.16)	0.20	54
CR-POPF	7	617 vs 1024	0.77 (0.55–1.07)	0.12	0
POPF	8	681 vs 1088	0.79 (0.56–1.10)	0.16	9
Bile leak	4	343 vs 791	0.93 (0.46–1.88)	0.84	0
SSI	7	618 vs 1025	0.72 (0.51–1.03)	0.07	19
DGE	8	681 vs 1088	0.99 (0.70–1.40)	0.96	25
Reintervention	4	303 vs 777	0.58 (0.22–1.50)	0.26	1
Readmission	5	260 vs 743	0.91 (0.59–1.41)	0.68	0
Length of stay (days)	6	500 vs 462	− 0.20 (− 1.68 to 1.28)	0.80	59
Data from randomised clinical trials					
Mortality	5	277 vs 312	1.12 (0.32–3.86)	0.86	0
Major morbidity	5	277 vs 312	0.78 (0.39–1.59)	0.50	42
Minor morbidity	4	249 vs 278	0.67 (0.40–1.14)	0.14	29
Global morbidity	5	277 vs 312	0.61 (0.29–1.30)	0.20	59
CR-POPF	5	277 vs 312	0.68 (0.40–1.15)	0.15	23
POPF	5	277 vs 312	0.79 (0.52–1.21)	0.28	14
Bile leak	2	162 vs 165	0.62 (0.16–2.44)	0.49	31
**SSI**	**4**	**214 vs 249**	**0.53 (0.32–0.91)**	0.02*	**0**
DGE	5	277 vs 312	0.75 (0.39–1.44)	0.38	42
Reintervention	3	186 vs 215	0.30 (0.07–1.27)	0.10	0
Readmission	4	143 vs 181	1.04 (0.48–2.25)	0.92	0
Length of stay (days)	5	277 vs 312	− 0.38 (− 2.54–1.78)	0.73	67
Data on patients undergoing pancreatoduodenectomy					
Mortality	6	458 vs 938	1.05 (0.42–2.60)	0.92	0
Major morbidity	7	617 vs 1024	0.91 (0.63–1.30)	0.59	22
Minor morbidity	4	301 vs 579	0.67 (0.39–1.15)	0.15	56
**Global morbidity**	**6**	**540 vs 1012**	**0.61 (0.39–0.98)**	0.04*	**42**
CR-POPF	5	412 vs 827	0.72 (0.47–1.09)	0.12	0
POPF (overall)	5	410 vs 825	0.69 (0.46–1.03)	0.07	0
Bile leak	3	192 vs 382	0.96 (0.44–2.08)	0.91	11
**SSI**	**6**	**523 vs 1013**	**0.57 (0.40–0.82)**	0.003*	**0**
DGE	4	368 vs 742	0.72 (0.45–1.14)	0.16	20
Reintervention	2	118 vs 199	0.57 (0.17–1.99)	0.38	28
Readmission	3	180 vs 666	0.82 (0.49–1.35)	0.43	0
**LOS (days)**	**3**	**197 vs 235**	**− 1.57 (− 3.05 to − 0.09)**	0.04*	**0**

Asterisk indicates statistically signifi cant values (*p* < 0.05)

*CCS* Corticosteroids, *OR* Odd ratio, *MD* Mean difference, *CR-POPF* Clinically relevant post-operative pancreatic fistula, *POPF* Post-operative pancreatic fistula, *SSI* Surgical site infection, *DGE* Delayed gastric emptying, *LOS* Length of stay

### Data from randomised clinical trials

**Fig. 2:**
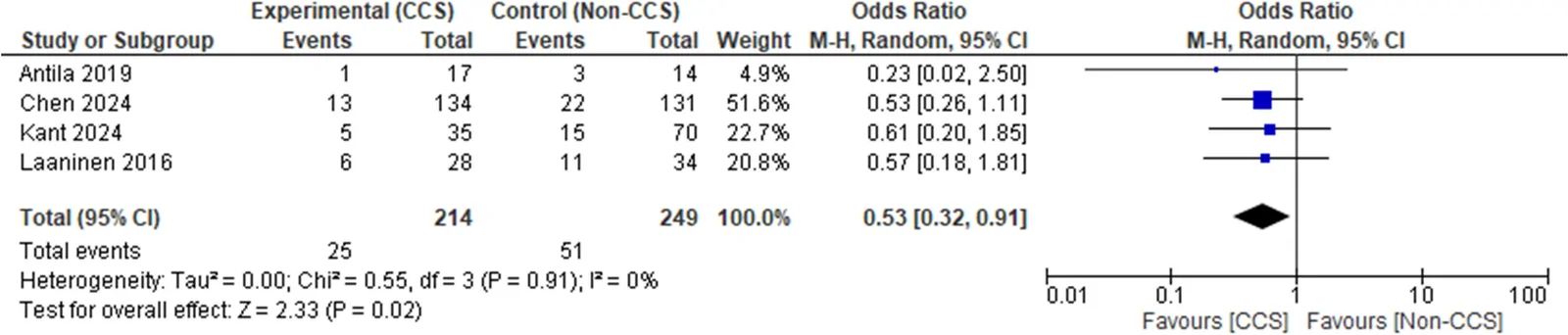
Forest plot of randomized controlled trials. Comparison of surgical site infection between patients receiving perioperative corticosteroids and controls RCT subgroup)

When restricting the analysis to RCTs (n = 554 patients) CCS showed a significant reduction in SSI in comparison with non-CCS (OR 0.53, 95% CI 0.32–0.91, *p* = 0.02, I^2^ = 0%) (Fig. 2 and Table 3). No significant differences were found in mortality, major, minor and global morbidity, POPF, bile leak, DGE or reintervention or readmission rates.

### Data on patients submitted to pancreatoduodenectomy

**Fig. 3:**
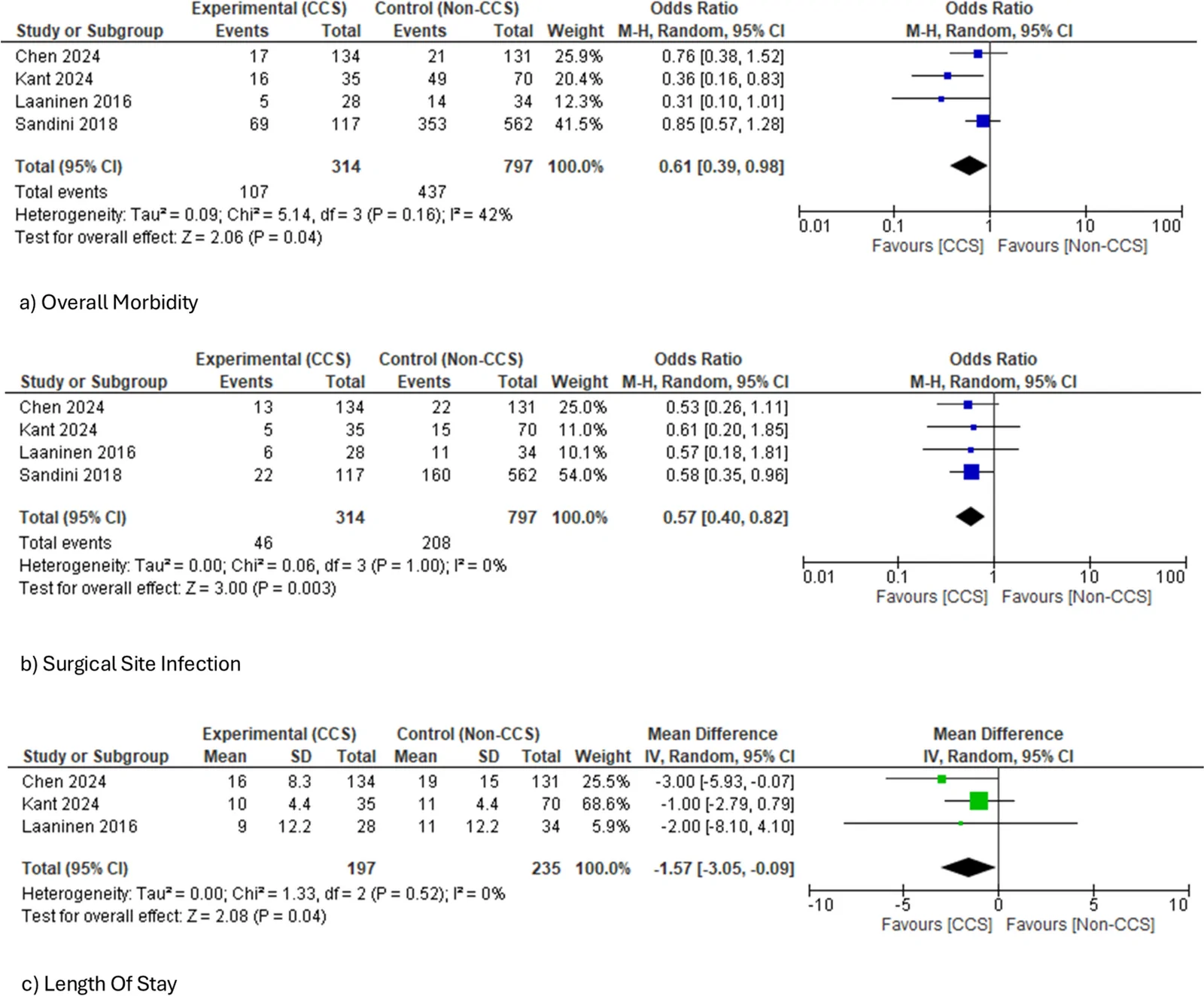
Forest plots of pancreatoduodenectomy subgroup, **a** Overall morbidity; **b** Surgical site infection; **c** Postoperative length of stay

In patients undergoing pancreaticoduodenectomy (n = 1076 patients) CCS was associated with a significant reduction in overall morbidity (OR 0.61, 95% CI 0.39–0.98, *p* = 0.04), a lower incidence of SSI (OR 0.57, 95% CI 0.40–0.82, *p* = 0.003), and a significantly shortened postoperative LOS with non-CCS (MD –1.57 days, 95% CI –3.05 to –0.09, *p* = 0.04) (Fig. 3 and Table 3). No significant differences were observed in mortality, major morbidity, POPF, bile leak, DGE, reintervention, or hospital readmission.

### Sensitivity analysis from data on hydrocortisone and dexamethasone

A sensitivity analyses stratified by steroid type showed that neither the subgroup of studies on hydrocortisone (4 studies, n = 289 patients), nor those on dexamethasone (4 studies, n = 1461 patients) presented significant differences in any of the assessed outcomes compared with non-CCS (Supplementary Table 3).

### Human expertise versus artificial intelligence (ChatGPT): correlation analysis

Pairwise correlation of matched variables across the two datasets demonstrated good agreement. Pearson correlation coefficients ranged from 0.97 to 1.00 for most variables, including sample size, patient allocation, mortality, CR-POPF, pancreatic fistula, postoperative haemorrhage, delayed gastric emptying, SSI, reintervention, and readmission. Most correlations were statistically significant (p < 0.001) (Supplementary Table 4). A heatmap representation confirmed the strong consistency of results between datasets (Supplementary Fig. 1). Despite the overall high concordance, some substantial discrepancies (> 5% relative difference) were found as detailed in Supplementary Table 5.

## Discussion

This systematic review and meta-analysis evaluated the role of CCS in the perioperative setting, providing a practical overview of the CCS administration. Across nine studies with 1913 patients, including five randomized controlled trials, the analysis found that CCS administration did not significantly influence overall mortality major morbidity, POPF, or other procedure-specific complications. However, perioperative CCS may reduce the risk of SSI and overall morbidity in patients undergoing PD and may also be associated with a decrease in postoperative LOS.

These findings are consistent with the well-established anti-inflammatory effects of CCS, which have previously demonstrated benefits in other surgical domains such as hepatic and colorectal resections [13–18]. RCT on major hepatectomy have shown that a single preoperative dose of methylprednisolone significantly reduces postoperative IL-6 and attenuates early SIRS without increasing infectious morbidity [13, 41, 42]. Additional studies reported improved early liver function and shorter hospital stay [43]. Likewise, in colorectal and inflammatory bowel disease surgery, perioperative dexamethasone has been associated with reduced postoperative ileus, decreased inflammatory response, and improved short-term recovery, with no consistent increase in wound complications or anastomotic leakage [15, 44]. In the present analysis, the reduction in SSI observed in the RCT subgroup (OR 0.53, 95% CI 0.32–0.91) and in patients undergoing PD (OR 0.57, 95% CI 0.40–0.82) suggests that perioperative CCS may attenuate the systemic inflammatory response and improve tissue healing [34]. Importantly, these potential benefits were not accompanied by an increased risk of post-operative complications such as POPF or bile leakage, complications typically feared in association with CCS use [34]. Also, none of the included studies reported an increased incidence of post-pancreatectomy diabetes or steroid-related adverse events or in the CCS group. This supports the hypothesis that short-term perioperative administration is safe and does not replicate the adverse effects observed with chronic steroid therapy [30, 35, 36, 38].

Considering the growing body of literature recognising PPAP as a key driver of major postoperative complications [9–12, 45], the findings from the present may raise intriguing questions on the potential relevance of CCS in pancreatic surgery. The inflammatory cascade triggered by remnant injury and enzymatic activation may exacerbate local tissue damage and impair healing [46]. In this context, CCS could theoretically mitigate inflammatory severity, reduce oedema, and improve perfusion, particularly at the anastomotic site [14]. However, the present evidence does not yet allow definitive conclusions, and further research is required to clarify whether CCS directly modulate the risk of PPAP.

Considering the absence of a clear standard of care, given that no international guideline currently recommends for or against the routine use of perioperative CCS [22, 23], this review contributes to a growing body of literature, including a recent systematic review reporting no definitive association between CCS and post-pancreatectomy outcomes [19]. However, while Liu et al. aimed to focus on PD, their analysis included papers assessing patients not only undergoing PD but also other pancreatic resections [37] which may have increased heterogeneity and influenced the final estimates. By contrast, subgroup analyses from RCT in the present review, providing a cleaner estimate of effect and limiting confounding, suggested an association between CCS and reductions in overall complications and specifically SSI. These RCT-focused data were not reported in Liu et al.’s analysis.

While the methodological quality of the included studies represents a relative strength, five out nine studies being a RCT and none judged as having high risk of bias, sample sizes remained modest, particularly in the hydrocortisone subgroup and none of included studies stratified patients according to risk models for postoperative complications in pancreatic surgery or the development of PPAP [5, 6, 8, 47]. Also, it should be considered that the observed benefits were not universal across all analyses [33, 37]. This heterogeneity may reflect differences in dosing regimens, duration of administration, and patient selection across studies. While the present review provides a global overview of current trends in perioperative CCS administration, no clear advantage of a specific regimen, for examples intraoperative-only or continued postoperative CCS administration emerged. Similarly, no clear signal identified which patients, such as those at higher risk of complications or with PPAP, might benefit most from CCS. Moreover, the lack of significant effect on POPF, despite theoretical benefits on reducing indicates that the pathophysiology of procedure-specific complications likely extends beyond systemic inflammatory modulation [3, 21].

A further novelty of this review lies in the incorporation of ChatGPT into evidence synthesis [48, 49]. Correlation analyses demonstrated good agreement between AI-assisted and manual data extraction, supporting the feasibility of AI as a complementary tool for enhancing efficiency and reproducibility in surgical research. This aligns with the growing literature on AI applications in biomedical research, where natural language processing and large language models are increasingly employed for tasks such as automated screening of abstracts and data extraction [50–52]. However, despite the overall strong concordance, discrepancies were still identified. These errors highlight both the promise and the limitations of current general-purpose AI tools, reinforcing the need for continuous human oversight and expert clinical interpretation [51, 52]. Therefore, while AI may reduce the workload of some clinical research-related tasks, current tools, particularly general-purpose platforms such as ChatGPT not specifically designed for this purpose, still require further.

This study has limitations. The overall number of included patients was limited, and heterogeneity in surgical techniques, perioperative care, and CCS dosing regimens complicates pooled interpretation. In particular, substantial heterogeneity in corticosteroid regimens, including type, dosage, timing, and duration of administration, limits the ability to identify an optimal therapeutic strategy. Furthermore, some subgroup findings, such as reduction in SSI, were statistically significant only in specific populations, and caution is warranted when extrapolating to all category of patients and pancreatic resections. Also, long-term outcomes, such as quality of life and oncological results, remain largely unexplored. refinement before they can be considered as reliable as human effort. Finally, the absence of a standardized prompting framework and the variability in AI interpretation further underscore the need for structured workflows in future applications.

## Conclusions

Perioperative administration of corticosteroids appears to be safe in pancreatic surgery but does not provide consistent benefit across major outcomes. A potential reduction in surgical site infections may be observed in selected subgroups, particularly in pancreatoduodenectomy. However, current evidence is insufficient to support routine use. Future large, multicentre randomized trials are needed to define optimal regimens and identify patient populations most likely to benefit.

The integration of artificial intelligence into systematic reviews is feasible and shows promise for improving efficiency and reproducibility. At present, however, AI tools remain complementary to expert human judgment and require further refinement before independent application in surgical research.

## Supplementary Information

Below is the link to the electronic supplementary material.

Supplementary file1: Supplementary Figure 1: Heatmap of Pearson’s correlation coefficients (r) for all matched outcomes, ordered according to the original dataset structure. The analysis demonstrates good correlation (r = 0.97–1.00, p < 0.001) across most outcomes, confirming consistency between datasets.

Supplementary file2 (PDF 631 KB)

Supplementary file3 (DOCX 30 KB)

## Data Availability

Data will be provided upon resonable requests.
