# Supplementary material for: Perioperative corticosteroids and pancreatic surgery outcomes: a systematic review and meta-analysis combining human expertise and AI support (ChatGPT)
Source: Updates Surg. 2026 Jul 8;78(6):2549–59. doi: 10.1007/s13304-026-02750-3 (PMC13737091; doi:10.1007/s13304-026-02750-3)
Supplement: Supplementary file 1 [file 13304_2026_2750_MOESM1_ESM.pdf]

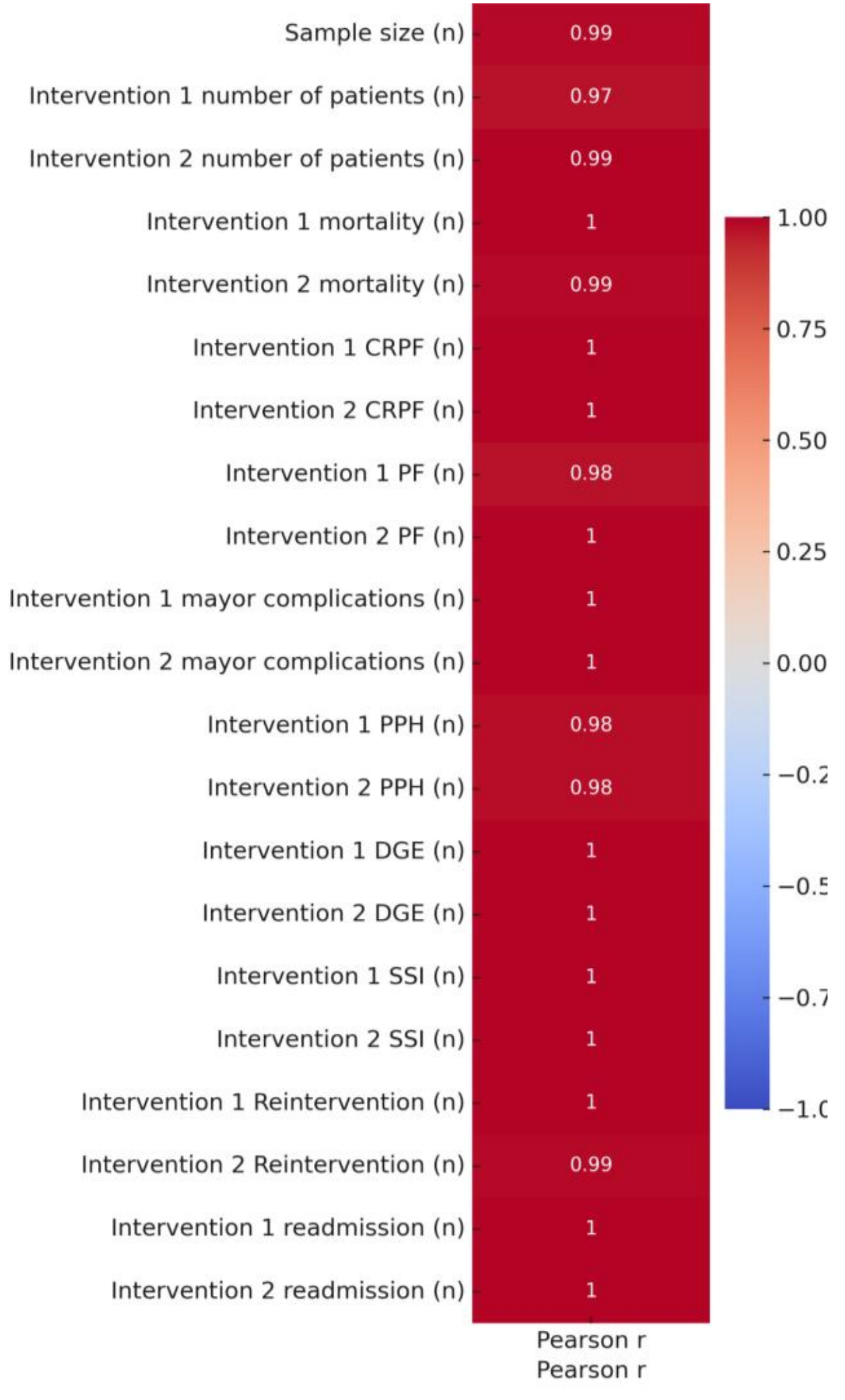
Sample size (n) 0.99
Intervention 1 number of patients (n) 0.97
Intervention 2 number of patients (n) 0.99
Intervention 1 mortality (n) 1
Intervention 2 mortality (n) 0.99
Intervention 1 CRPF (n) 1
Intervention 2 CRPF (n) 1
Intervention 1 PF (n) 0.98
Intervention 2 PF (n) 1
Intervention 1 mayor complications (n) 1
Intervention 2 mayor complications (n) 1
Intervention 1 PPH (n) 0.98
Intervention 2 PPH (n) 0.98
Intervention 1 DGE (n) 1
Intervention 2 DGE (n) 1
Intervention 1 SSI (n) 1
Intervention 2 SSI (n) 1
Intervention 1 Reintervention (n) 1
Intervention 2 Reintervention (n) 0.99
Intervention 1 readmission (n) 1
Intervention 2 readmission (n) 1
Pearson r
Pearson r
1.00
0.75
0.50
0.25
0.00
