# Supplementary material for: Perioperative corticosteroids and pancreatic surgery outcomes: a systematic review and meta-analysis combining human expertise and AI support (ChatGPT)
Source: Updates Surg. 2026 Jul 8;78(6):2549–59. doi: 10.1007/s13304-026-02750-3 (PMC13737091; doi:10.1007/s13304-026-02750-3)

CCS vs Non-CCS: 1.Mortality

	Experimental (CCS)		Control (Non-CCS)			Odds Ratio
Study or Subgroup	Events	Total	Events	Total	Weight	M-H, Random, 95% CI
Antila 2019	0	17	0	14		Not estimable
Chen 2024	3	134	3	131	31.7%	0.98 [0.19, 4.93]
Kant 2024	0	35	1	70	8.0%	0.65 [0.03, 16.43]
Laaninen 2016	0	28	0	34		Not estimable
Sandini 2018	2	117	10	562	35.5%	0.96 [0.21, 4.44]
Tarvainen 2020	2	63	1	63	14.1%	2.03 [0.18, 23.01]
Zhang 2021	1	64	1	64	10.7%	1.00 [0.06, 16.34]
**Total (95% CI)**		**458**		**938**	**100.0%**	**1.05 [0.42, 2.60]**
Total events	8		16			

Heterogeneity: Tau² = 0.00; Chi² = 0.39, df = 4 (P = 0.98); I² = 0%
Test for overall effect: Z = 0.10 (P = 0.92)

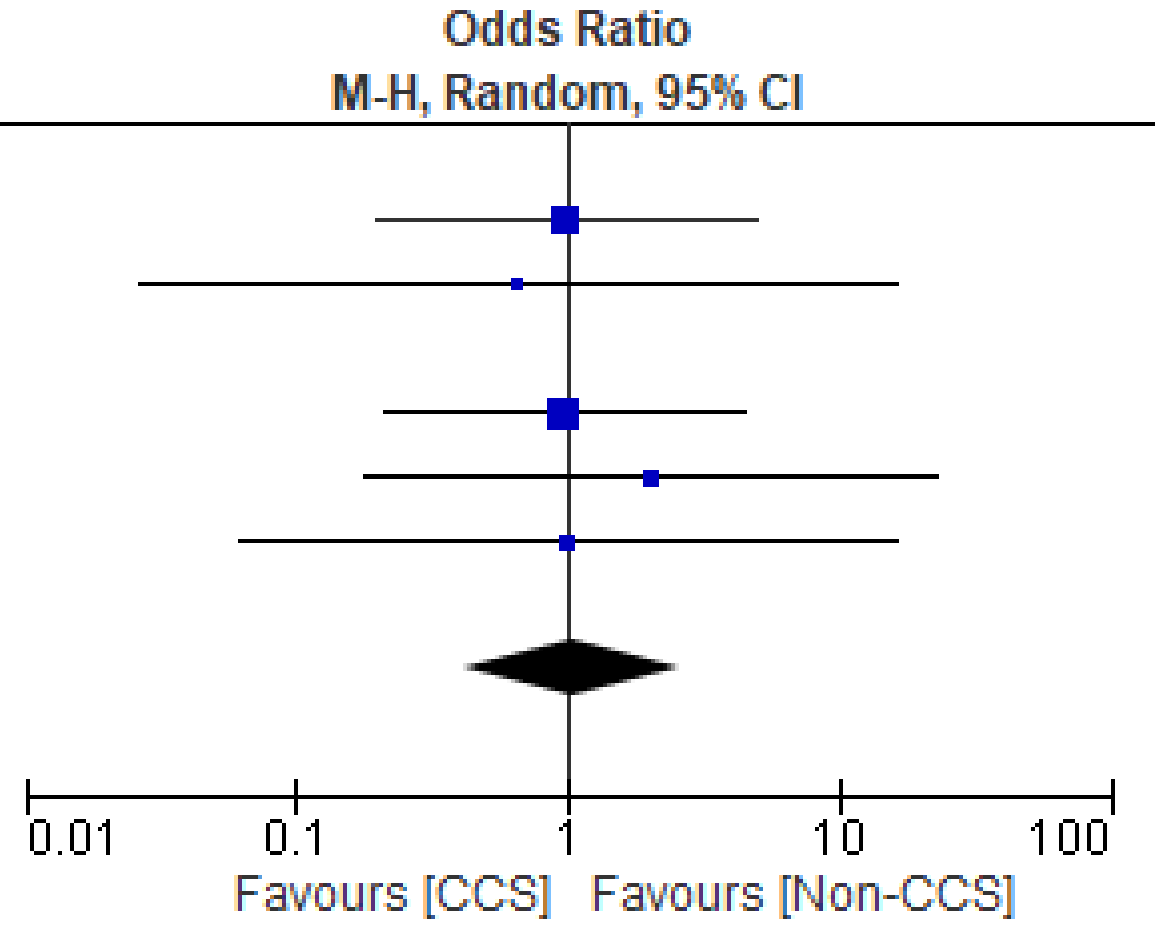

CCS vs Non-CCS: 2.Morbidity mayor

Study or Subgroup	Experimental (CCS) Events	Experimental (CCS) Total	Control (Non-CCS) Events	Control (Non-CCS) Total	Weight	Odds Ratio M-H, Random, 95% CI
Antila 2019	1	17	3	14	2.2%	0.23 [0.02, 2.50]
Chen 2024	17	134	21	131	19.5%	0.76 [0.38, 1.52]
Kant 2024	3	35	5	70	5.4%	1.22 [0.27, 5.42]
Laaninen 2016	5	28	14	34	8.2%	0.31 [0.10, 1.01]
Newhook 2021	47	223	29	150	28.2%	1.11 [0.66, 1.87]
Sandini 2018	20	117	104	562	27.7%	0.91 [0.54, 1.54]
Tarvainen 2020	10	63	5	63	8.8%	2.19 [0.70, 6.82]
**Total (95% CI)**		**617**		**1024**	**100.0%**	**0.91 [0.63, 1.30]**
Total events	103		181			

Heterogeneity: Tau² = 0.05; Chi² = 7.72, df = 6 (P = 0.26); I² = 22%

Test for overall effect: Z = 0.53 (P = 0.59)

Odds Ratio
M-H, Random, 95% CI

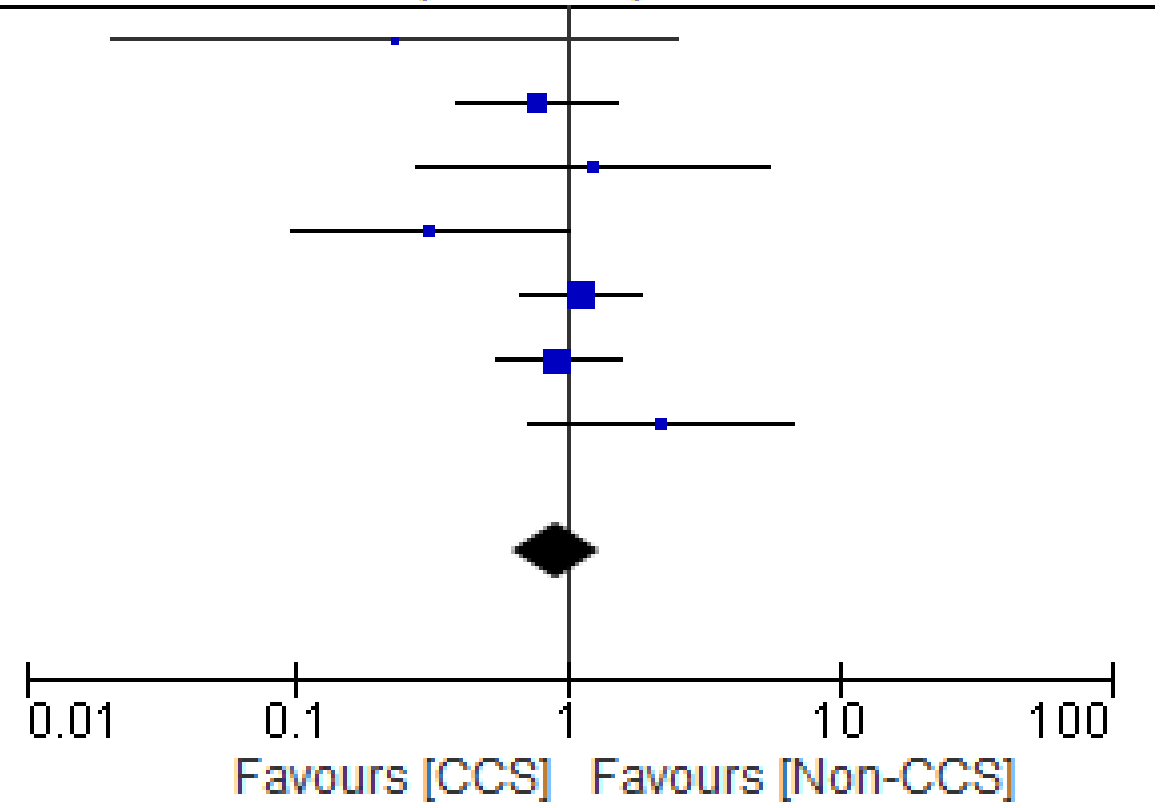

CCS vs Non-CCS: 3.Morbidity minor

Study or Subgroup	Experimental (CCS) Events	Experimental (CCS) Total	Control (Non-CCS) Events	Control (Non-CCS) Total	Weight	Odds Ratio M-H, Random, 95% CI
Antila 2019	10	17	9	14	5.9%	0.79 [0.18, 3.41]
Chen 2024	17	134	21	131	21.3%	0.76 [0.38, 1.52]
Kant 2024	13	35	45	70	15.6%	0.33 [0.14, 0.76]
Sandini 2018	49	117	249	562	41.9%	0.91 [0.61, 1.36]
Tarvainen 2020	50	63	49	63	15.3%	1.10 [0.47, 2.57]
**Total (95% CI)**		**366**		**840**	**100.0%**	**0.76 [0.53, 1.10]**
Total events	139		373			

Heterogeneity: Tau² = 0.04; Chi² = 5.21, df = 4 (P = 0.27); I² = 23%
Test for overall effect: Z = 1.45 (P = 0.15)

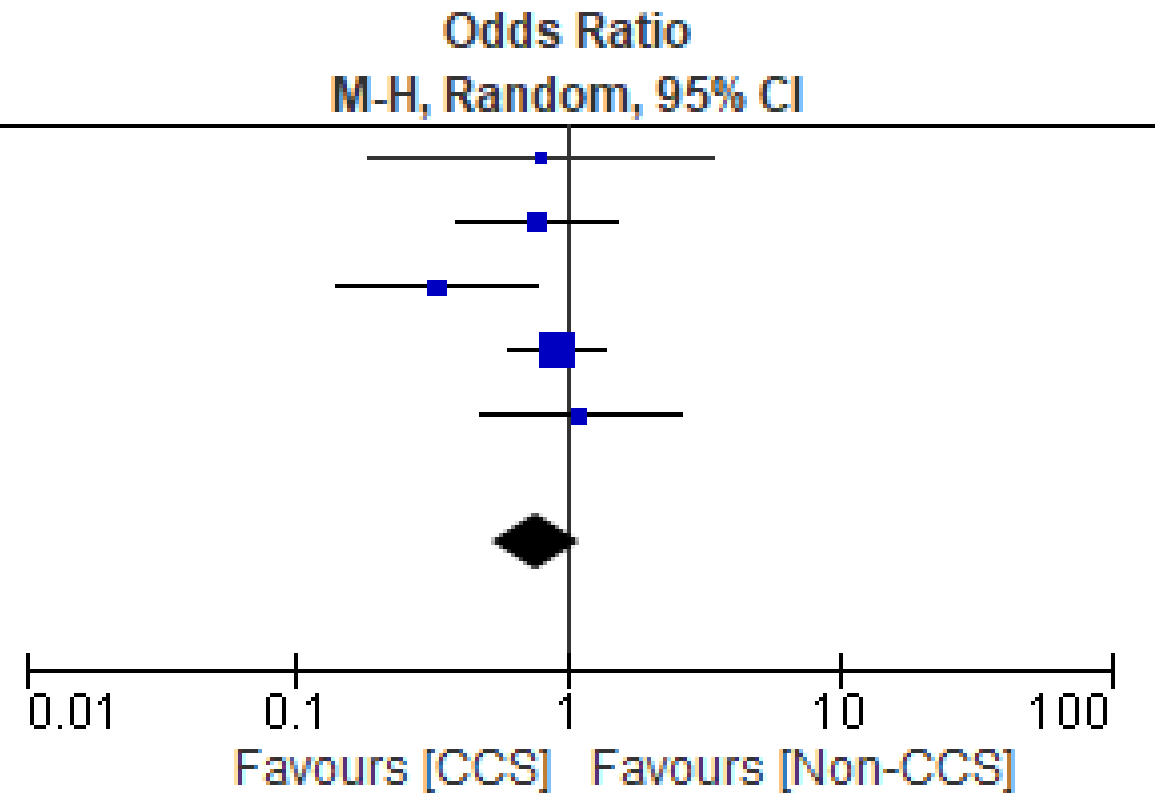

CCS vs Non-CCS: 4.Morbidity global

Study or Subgroup	Experimental (CCS) Events	Experimental (CCS) Total	Control (Non-CCS) Events	Control (Non-CCS) Total	Weight	Odds Ratio M-H, Random, 95% CI	Odds Ratio M-H, Random, 95% CI
Antila 2019	11	17	12	14	5.0%	0.31 [0.05, 1.84]	
Chen 2024	17	134	21	131	17.4%	0.76 [0.38, 1.52]	
Kant 2024	16	35	49	70	14.5%	0.36 [0.16, 0.83]	
Laaninen 2016	5	28	14	34	9.5%	0.31 [0.10, 1.01]	
Newhook 2021	47	223	29	150	21.5%	1.11 [0.66, 1.87]	
Sandini 2018	69	117	353	562	24.3%	0.85 [0.57, 1.28]	
Tarvainen 2020	60	63	54	63	7.8%	3.33 [0.86, 12.95]	
**Total (95% CI)**		**617**		**1024**	**100.0%**	**0.75 [0.48, 1.16]**	
Total events	225		532				

Heterogeneity: Tau² = 0.16; Chi² = 12.94, df = 6 (P = 0.04); I² = 54%

Test for overall effect: Z = 1.28 (P = 0.20)

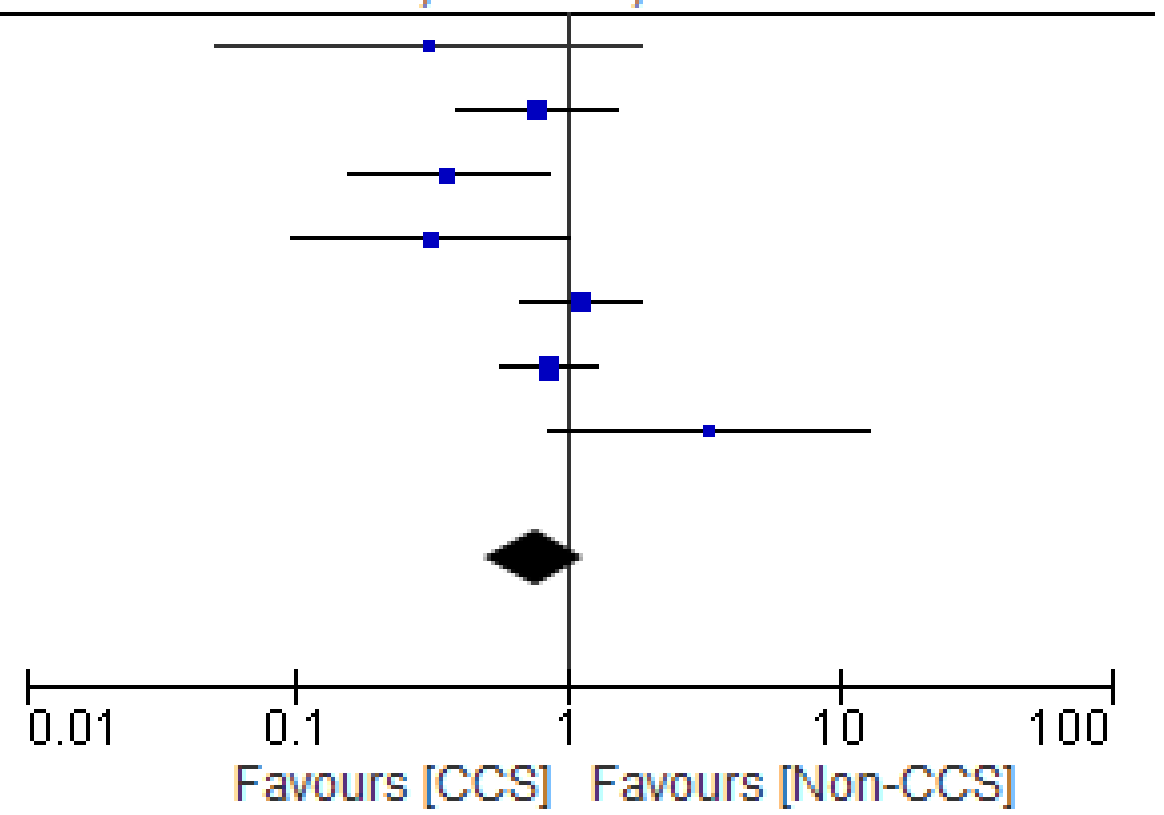

CCS vs Non-CCS: 5.CR-POPF

	Experimental (CCS)		Control (Non-CCS)			Odds Ratio	Odds Ratio
**Study or Subgroup**	**Events**	**Total**	**Events**	**Total**	**Weight**	**M-H, Random, 95% CI**	**M-H, Random, 95% CI**
Antila 2019	1	17	6	14	2.2%	0.08 [0.01, 0.82]	
Chen 2024	34	134	41	131	39.8%	0.75 [0.44, 1.28]	
Kant 2024	5	35	15	70	9.4%	0.61 [0.20, 1.85]	
Laaninen 2016	1	28	4	34	2.3%	0.28 [0.03, 2.64]	
Newhook 2021	14	223	10	150	16.2%	0.94 [0.41, 2.17]	
Sandini 2018	6	117	34	562	14.4%	0.84 [0.34, 2.05]	
Tarvainen 2020	14	63	13	63	15.8%	1.10 [0.47, 2.57]	
**Total (95% CI)**		**617**		**1024**	**100.0%**	**0.77 [0.55, 1.07]**	
Total events	75		123				

Heterogeneity: Tau² = 0.00; Chi² = 5.56, df = 6 (P = 0.47); I² = 0%

Test for overall effect: Z = 1.55 (P = 0.12)

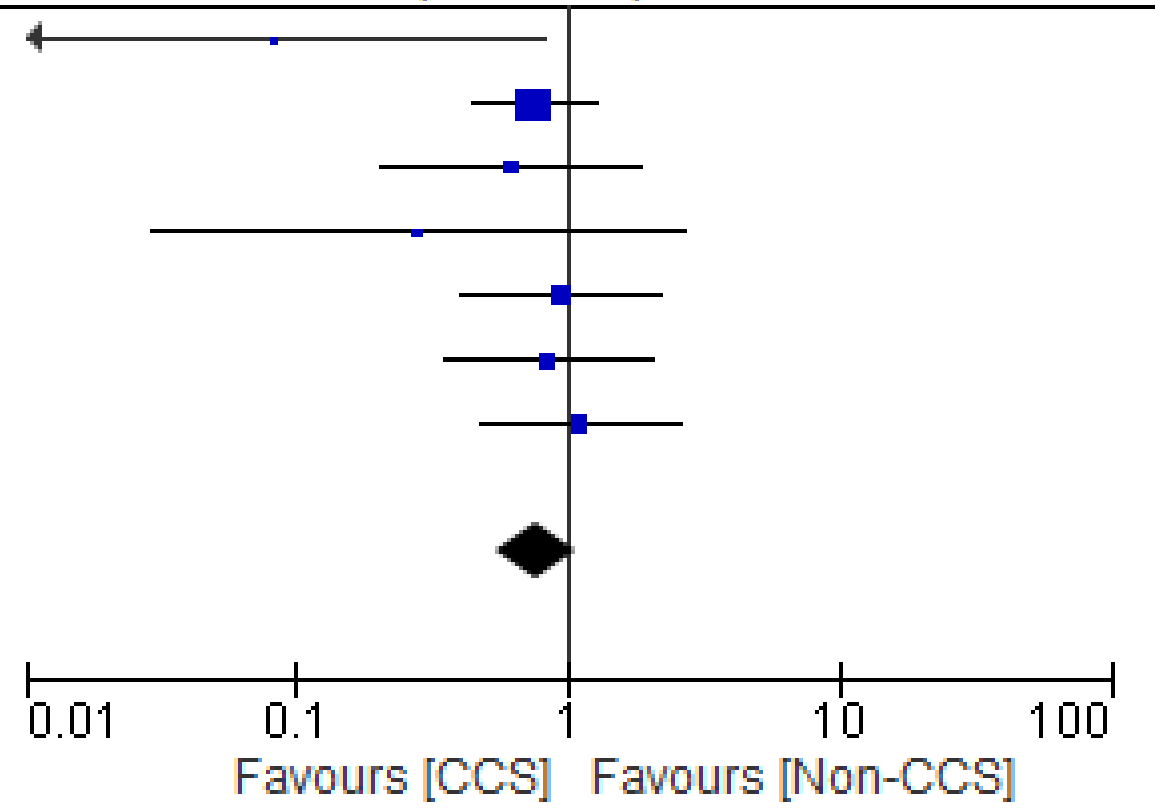

CCS vs Non-CCS: 6.POPF (biochemical leak and CR-POPF)

	Experimental (CCS)		Control (Non-CCS)			Odds Ratio
**Study or Subgroup**	**Events**	**Total**	**Events**	**Total**	**Weight**	**M-H, Random, 95% CI**
Antila 2019	7	17	7	14	5.4%	0.70 [0.17, 2.91]
Chen 2024	34	134	41	131	30.8%	0.75 [0.44, 1.28]
Kant 2024	5	35	15	70	8.7%	0.61 [0.20, 1.85]
Laaninen 2016	3	28	10	34	5.5%	0.29 [0.07, 1.18]
Newhook 2021	14	223	10	150	14.5%	0.94 [0.41, 2.17]
Sandini 2018	6	117	34	562	13.0%	0.84 [0.34, 2.05]
Tarvainen 2020	39	63	34	63	19.5%	1.39 [0.68, 2.82]
Zhang 2021	1	64	7	64	2.5%	0.13 [0.02, 1.08]
**Total (95% CI)**		**681**		**1088**	**100.0%**	**0.79 [0.56, 1.10]**
Total events	109		158			

Heterogeneity: $Tau^2 = 0.02$; $Chi^2 = 7.67$, $df = 7$ ($P = 0.36$); $I^2 = 9\%$

Test for overall effect: $Z = 1.40$ ($P = 0.16$)

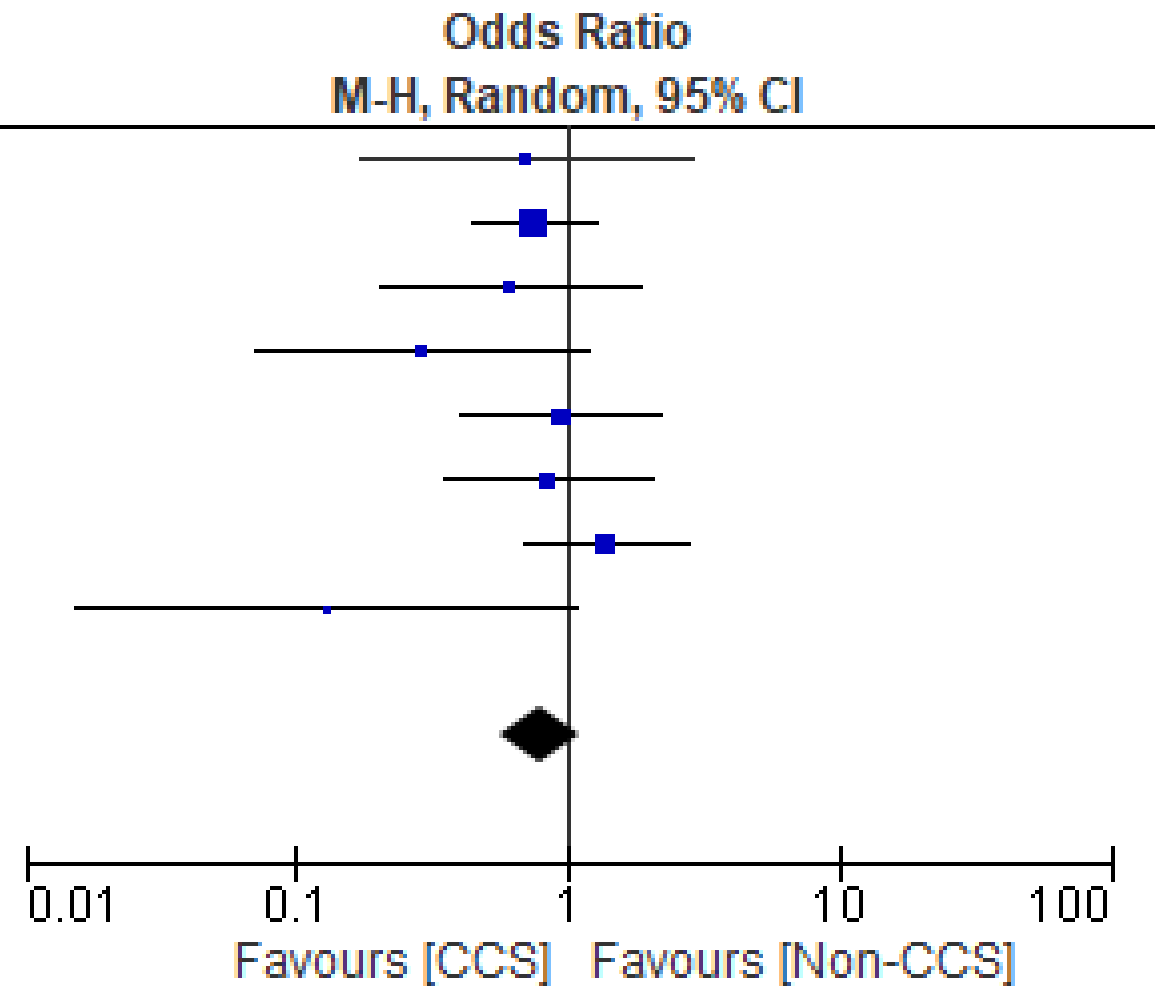

CCS vs Non-CCS: 7.Bile Leak

	Experimental (CCS)		Control (Non-CCS)			Odds Ratio	Odds Ratio
Study or Subgroup	Events	Total	Events	Total	Weight	M-H, Random, 95% CI	M-H, Random, 95% CI
Chen 2024	6	134	6	131	36.8%	0.98 [0.31, 3.11]	
Laaninen 2016	1	28	5	34	10.1%	0.21 [0.02, 1.96]	
Sandini 2018	5	117	18	562	48.3%	1.35 [0.49, 3.71]	
Zhang 2021	0	64	1	64	4.8%	0.33 [0.01, 8.21]	
**Total (95% CI)**		**343**		**791**	**100.0%**	**0.93 [0.46, 1.88]**	
Total events	12		30				

Heterogeneity: Tau² = 0.00; Chi² = 2.67, df = 3 (P = 0.45); I² = 0%

Test for overall effect: Z = 0.20 (P = 0.84)

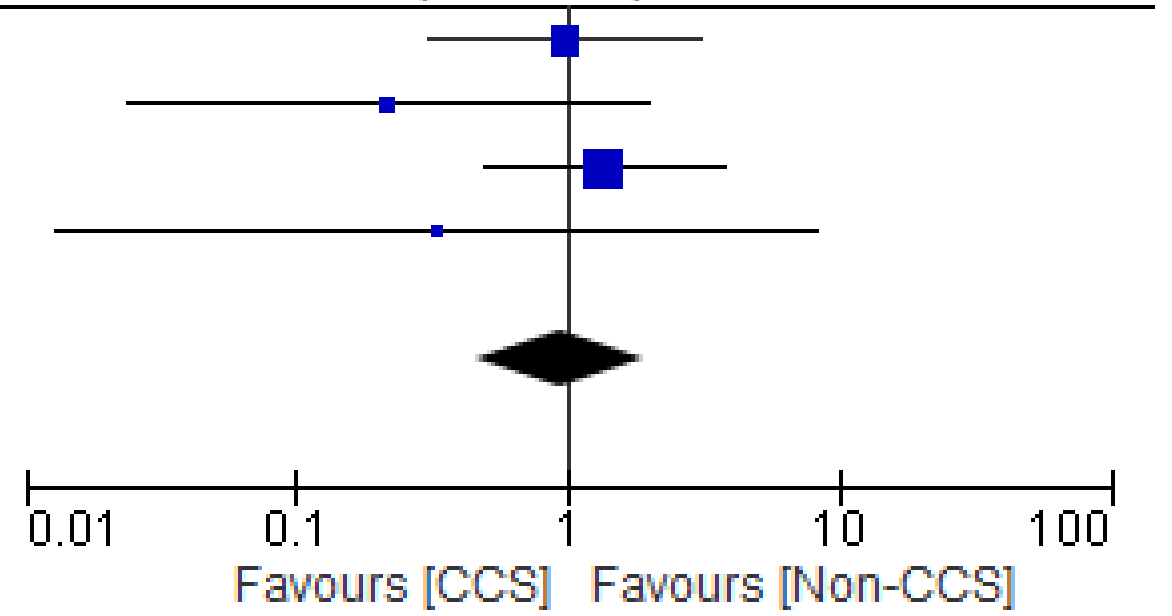

CCS vs Non-CCS: 8.SSI

Study or Subgroup	Experimental (CCS) Events	Experimental (CCS) Total	Control (Non-CCS) Events	Control (Non-CCS) Total	Weight	Odds Ratio M-H, Random, 95% CI
Antila 2019	1	17	3	14	2.0%	0.23 [0.02, 2.50]
Chen 2024	13	134	22	131	17.3%	0.53 [0.26, 1.11]
Kant 2024	5	35	15	70	8.7%	0.61 [0.20, 1.85]
Laaninen 2016	6	28	11	34	8.1%	0.57 [0.18, 1.81]
Newhook 2021	64	223	37	150	31.5%	1.23 [0.77, 1.97]
Sandini 2018	22	117	160	562	29.5%	0.58 [0.35, 0.96]
Zhang 2021	2	64	2	64	2.9%	1.00 [0.14, 7.33]
**Total (95% CI)**		**618**		**1025**	**100.0%**	**0.72 [0.51, 1.03]**
Total events	113		250			

Heterogeneity: $Tau^2 = 0.04$; $Chi^2 = 7.44$, df = 6 (P = 0.28); $I^2 = 19\%$

Test for overall effect: Z = 1.82 (P = 0.07)

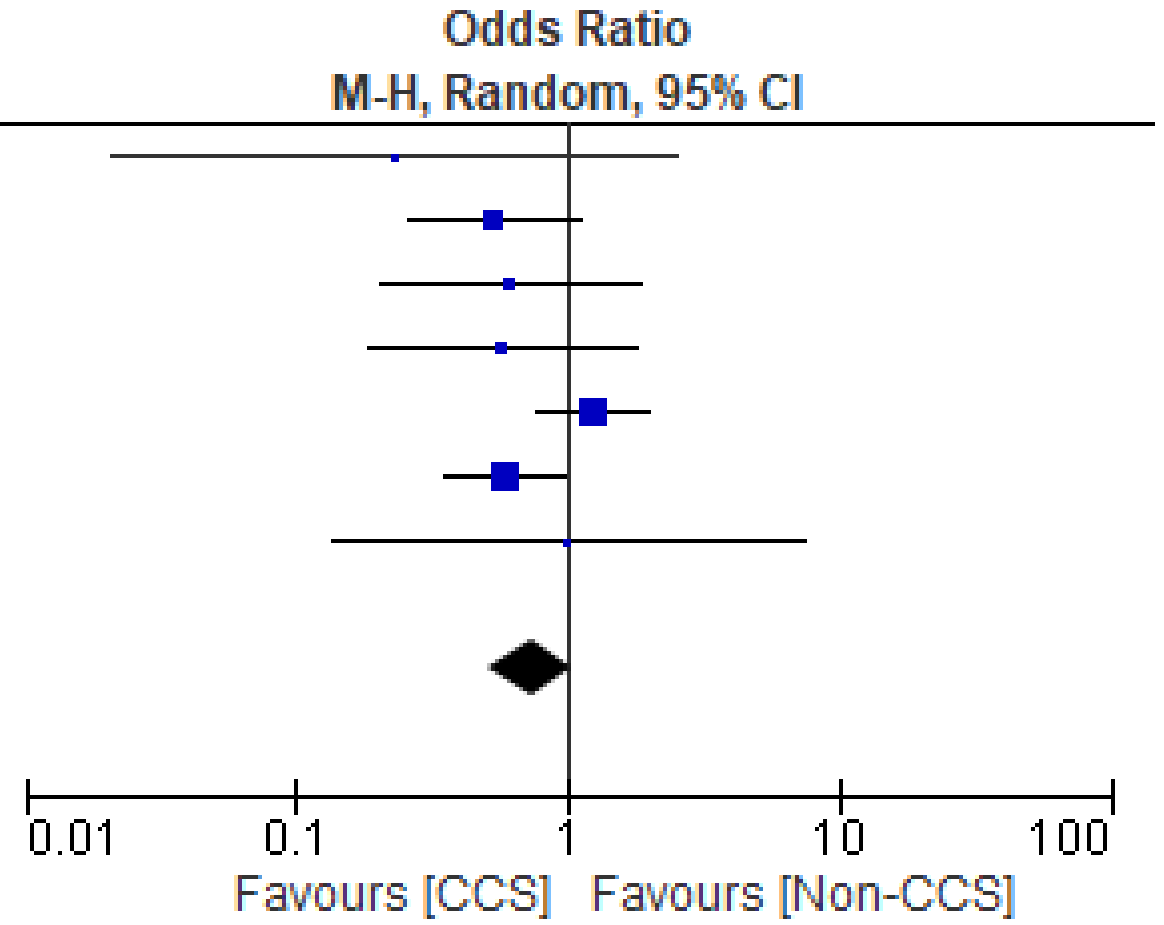

CCS vs Non-CCS: 9.DGE

	Experimental (CCS)		Control (Non-CCS)			Odds Ratio
Study or Subgroup	Events	Total	Events	Total	Weight	M-H, Random, 95% CI
Antila 2019	1	17	1	14	1.4%	0.81 [0.05, 14.28]
Chen 2024	13	134	17	131	14.8%	0.72 [0.33, 1.55]
Kant 2024	3	35	17	70	6.3%	0.29 [0.08, 1.08]
Laaninen 2016	8	28	15	34	8.9%	0.51 [0.17, 1.47]
Newhook 2021	60	223	33	150	26.0%	1.31 [0.80, 2.12]
Sandini 2018	19	117	90	562	23.2%	1.02 [0.59, 1.75]
Tarvainen 2020	19	63	12	63	13.2%	1.84 [0.80, 4.20]
Zhang 2021	6	64	4	64	6.2%	1.55 [0.42, 5.78]
**Total (95% CI)**		**681**		**1088**	**100.0%**	**0.99 [0.70, 1.40]**
Total events	129		189			

Heterogeneity: $Tau^2 = 0.06$; $Chi^2 = 9.33$, df = 7 (P = 0.23); $I^2 = 25\%$

Test for overall effect: Z = 0.05 (P = 0.96)

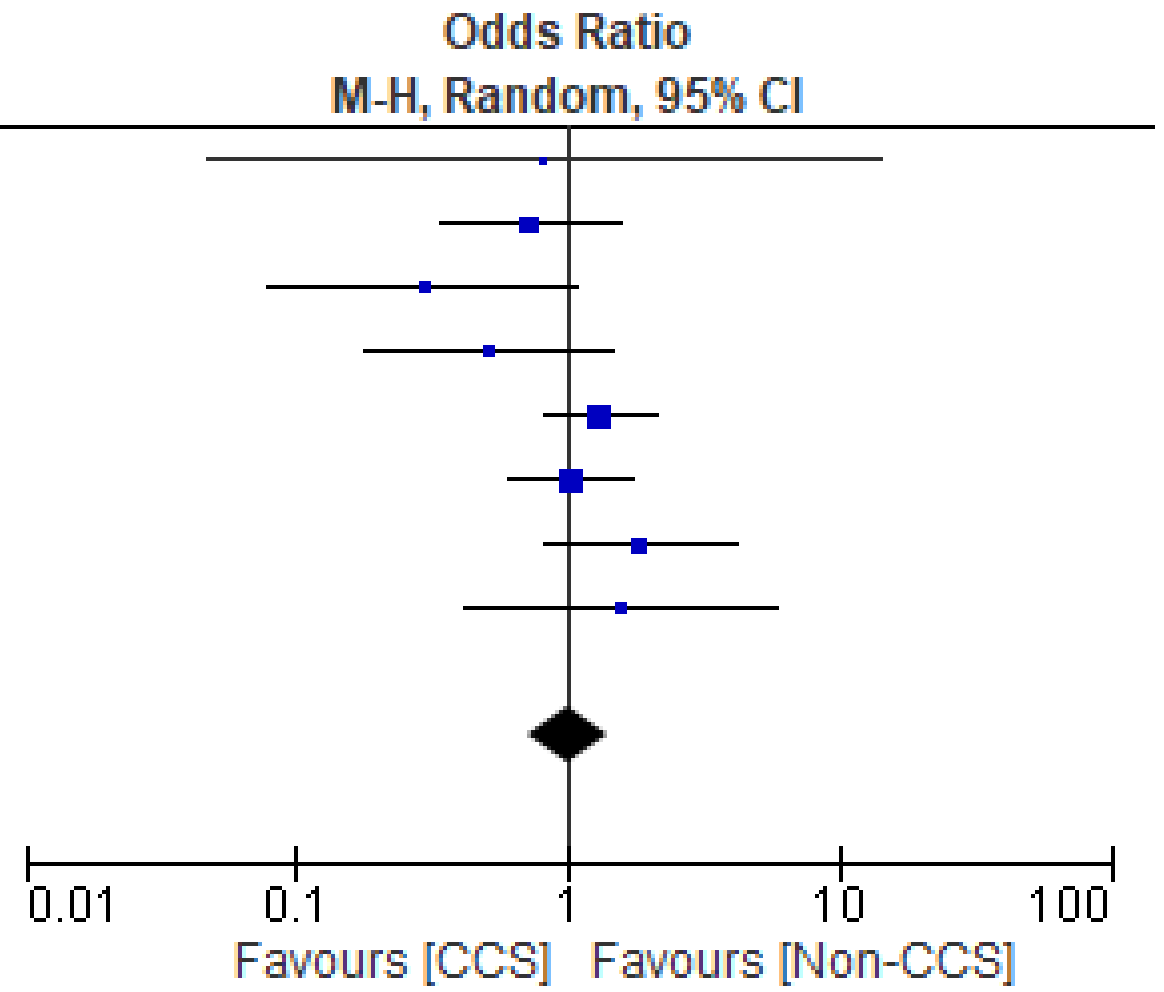

CCS vs Non-CCS: 10.Reintervention

Study or Subgroup	Experimental (CCS) Events	Experimental (CCS) Total	Control (Non-CCS) Events	Control (Non-CCS) Total	Weight	Odds Ratio M-H, Random, 95% CI
Antila 2019	0	17	1	14	8.4%	0.26 [0.01, 6.82]
Chen 2024	1	134	7	131	20.2%	0.13 [0.02, 1.10]
Kant 2024	1	35	2	70	15.1%	1.00 [0.09, 11.42]
Sandini 2018	3	117	15	562	56.3%	0.96 [0.27, 3.37]
**Total (95% CI)**		**303**		**777**	**100.0%**	**0.58 [0.22, 1.50]**
Total events	5		25			

Heterogeneity: Tau² = 0.01; Chi² = 3.02, df = 3 (P = 0.39); I² = 1%

Test for overall effect: Z = 1.12 (P = 0.26)

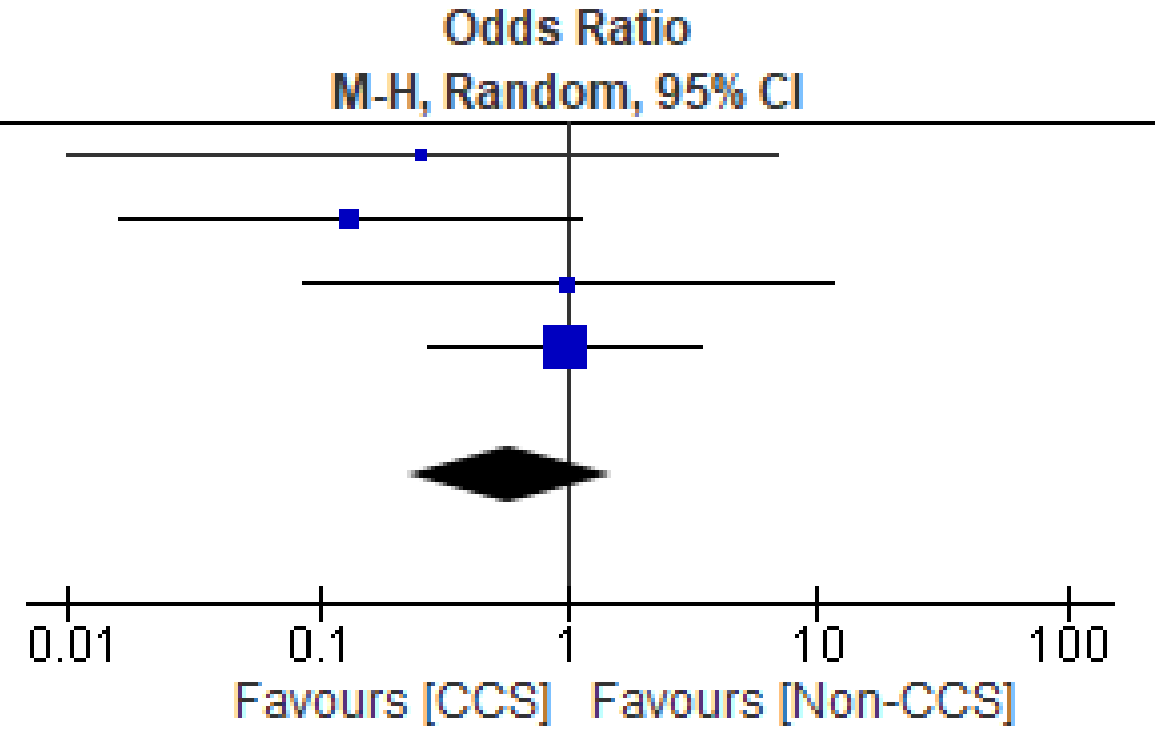

CCS vs Non-CCS: 11.Readmission

Study or Subgroup	Experimental (CCS) Events	Experimental (CCS) Total	Control (Non-CCS) Events	Control (Non-CCS) Total	Weight	Odds Ratio M-H, Random, 95% CI
Antila 2019	3	17	3	14	6.1%	0.79 [0.13, 4.68]
Kant 2024	2	35	4	70	6.3%	1.00 [0.17, 5.74]
Laaninen 2016	0	28	4	34	2.2%	0.12 [0.01, 2.31]
Sandini 2018	19	117	104	562	67.4%	0.85 [0.50, 1.46]
Tarvainen 2020	10	63	7	63	18.0%	1.51 [0.54, 4.25]
**Total (95% CI)**		**260**		**743**	**100.0%**	**0.91 [0.59, 1.41]**
Total events	34		122			

Heterogeneity: Tau² = 0.00; Chi² = 2.84, df = 4 (P = 0.58); I² = 0%

Test for overall effect: Z = 0.42 (P = 0.68)

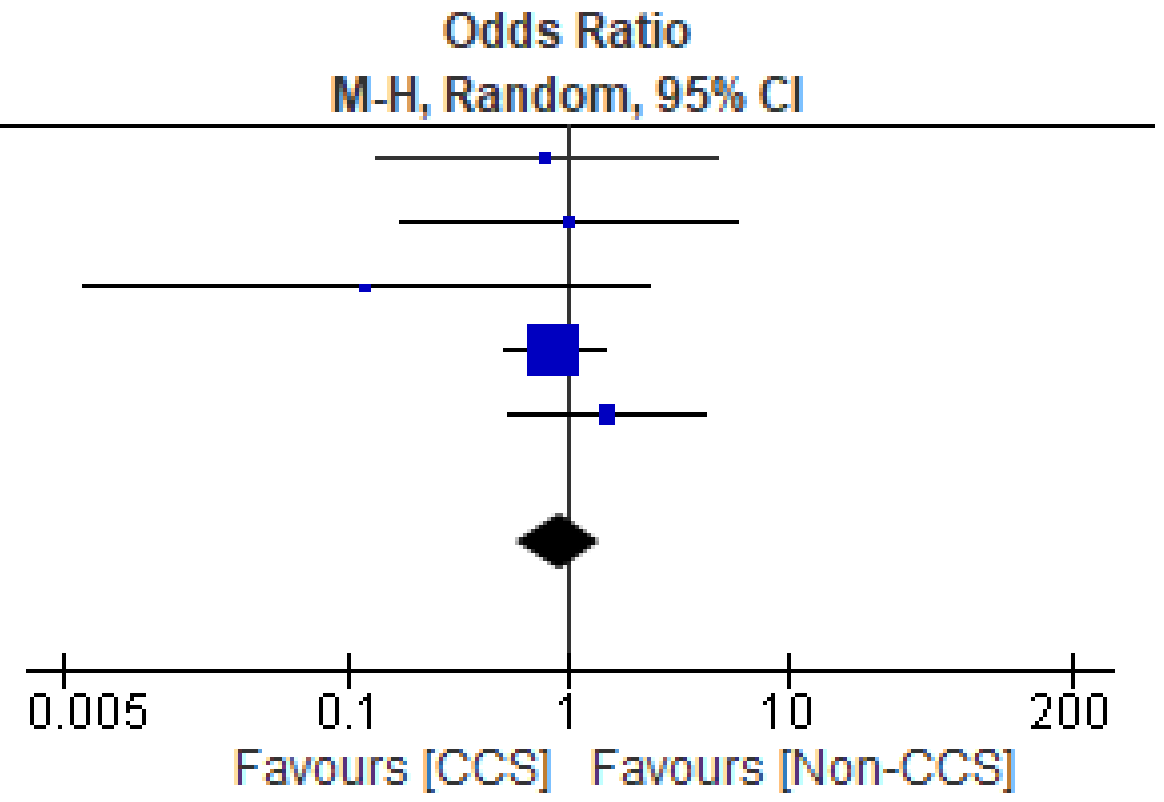

CCS vs Non-CCS: 12.LOS

Study or Subgroup	Experimental (CCS) Mean	SD	Total	Control (Non-CCS) Mean	SD	Total	Weight	Mean Difference IV, Random, 95% CI
Antila 2019	8	5.2	17	7	8	14	7.2%	1.00 [-3.87, 5.87]
Chen 2024	16	8.3	134	19	15	131	14.4%	-3.00 [-5.93, -0.07]
Kant 2024	10	4.4	35	11	4.4	70	22.4%	-1.00 [-2.79, 0.79]
Laaninen 2016	9	12.2	28	11	12.2	34	5.0%	-2.00 [-8.10, 4.10]
Newhook 2021	8	6.2	223	8	6.2	150	26.5%	0.00 [-1.28, 1.28]
Tarvainen 2020	10	4.4	63	8	4.4	63	24.4%	2.00 [0.46, 3.54]
**Total (95% CI)**			**500**			**462**	**100.0%**	**-0.20 [-1.68, 1.28]**

Heterogeneity: $Tau^2 = 1.72$; $Chi^2 = 12.25$, df = 5 (P = 0.03); $I^2 = 59\%$
Test for overall effect: Z = 0.26 (P = 0.80)

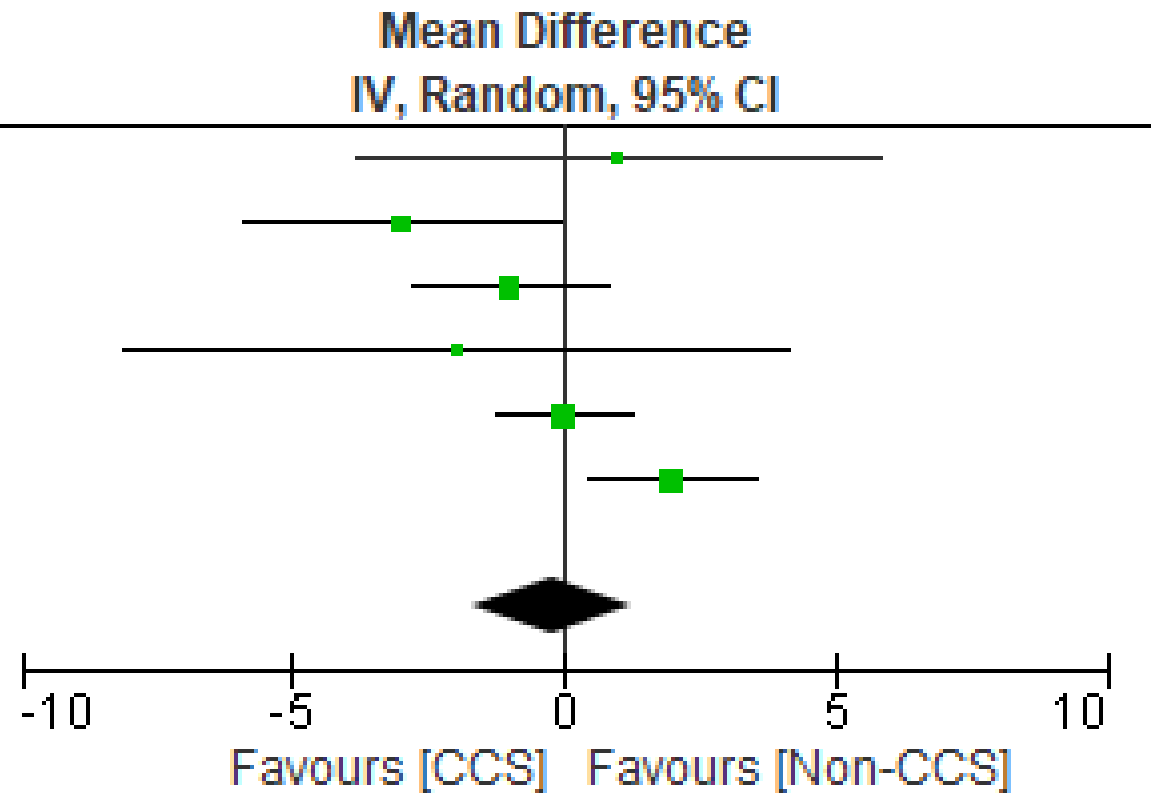

RCT - CCS vs Non-CCS: 1.Mortality

Study or Subgroup	Experimental (CCS) Events	Experimental (CCS) Total	Control (Non-CCS) Events	Control (Non-CCS) Total	Weight	Odds Ratio M-H, Random, 95% CI	Odds Ratio M-H, Random, 95% CI
Antila 2019	0	17	0	14		Not estimable	
Chen 2024	3	134	3	131	58.9%	0.98 [0.19, 4.93]	
Kant 2024	0	35	1	70	14.8%	0.65 [0.03, 16.43]	
Laaninen 2016	0	28	0	34		Not estimable	
Tarvainen 2020	2	63	1	63	26.2%	2.03 [0.18, 23.01]	
**Total (95% CI)**		**277**		**312**	**100.0%**	**1.12 [0.32, 3.86]**	
Total events	5		5				

Heterogeneity: $Tau^2 = 0.00$; $Chi^2 = 0.37$, df = 2 (P = 0.83); $I^2 = 0\%$

Test for overall effect: Z = 0.17 (P = 0.86)

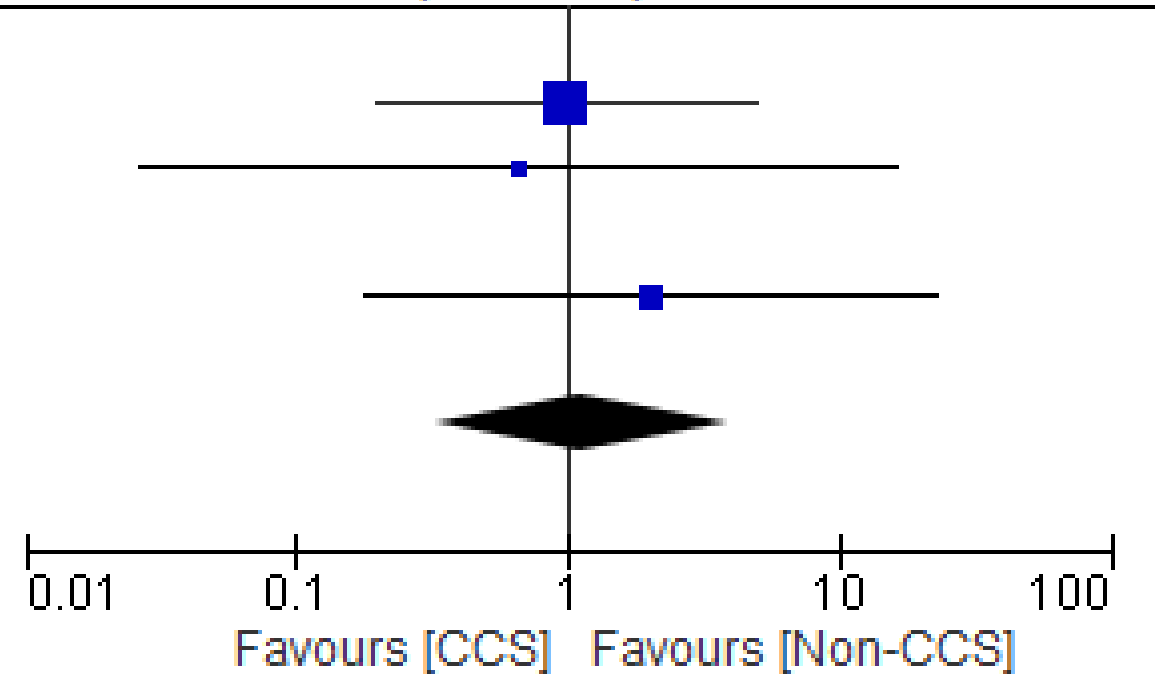

RCT - CCS vs Non-CCS: 2.Morbidity mayor

	Experimental (CCS)		Control (Non-CCS)			Odds Ratio	Odds Ratio
Study or Subgroup	Events	Total	Events	Total	Weight	M-H, Random, 95% CI	M-H, Random, 95% CI
Antila 2019	1	17	3	14	7.5%	0.23 [0.02, 2.50]	
Chen 2024	17	134	21	131	34.1%	0.76 [0.38, 1.52]	
Kant 2024	3	35	5	70	15.6%	1.22 [0.27, 5.42]	
Laaninen 2016	5	28	14	34	20.9%	0.31 [0.10, 1.01]	
Tarvainen 2020	10	63	5	63	21.9%	2.19 [0.70, 6.82]	
**Total (95% CI)**		**277**		**312**	**100.0%**	**0.78 [0.39, 1.59]**	
Total events	36		48				

Heterogeneity: Tau² = 0.26; Chi² = 6.84, df = 4 (P = 0.14); I² = 42%

Test for overall effect: Z = 0.68 (P = 0.50)

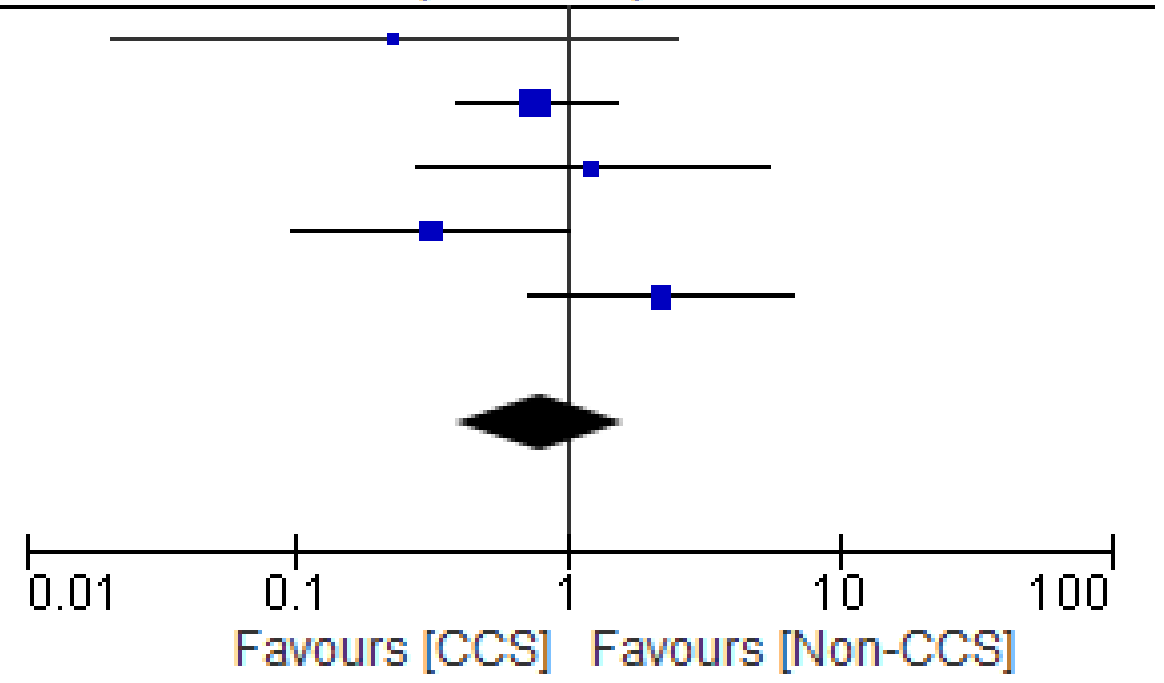

RCT - CCS vs Non-CCS: 3.Morbidity minor

Study or Subgroup	Experimental (CCS) Events	Experimental (CCS) Total	Control (Non-CCS) Events	Control (Non-CCS) Total	Weight	Odds Ratio M-H, Random, 95% CI
Antila 2019	10	17	9	14	11.4%	0.79 [0.18, 3.41]
Chen 2024	17	134	21	131	34.9%	0.76 [0.38, 1.52]
Kant 2024	13	35	45	70	27.1%	0.33 [0.14, 0.76]
Tarvainen 2020	50	63	49	63	26.6%	1.10 [0.47, 2.57]
**Total (95% CI)**		**249**		**278**	**100.0%**	**0.67 [0.40, 1.14]**
Total events	90		124			

Heterogeneity: Tau² = 0.09; Chi² = 4.24, df = 3 (P = 0.24); I² = 29%

Test for overall effect: Z = 1.47 (P = 0.14)

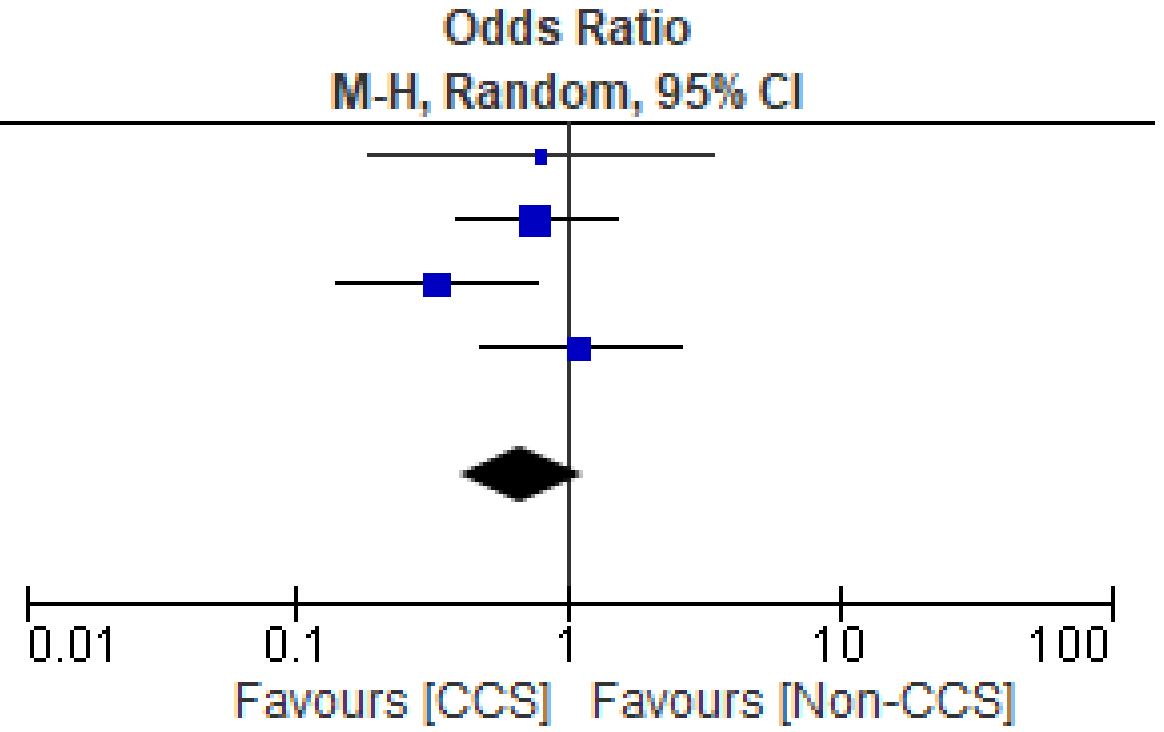

RCT - CCS vs Non-CCS: 4.Morbidity global

Study or Subgroup	Experimental (CCS) Events	Experimental (CCS) Total	Control (Non-CCS) Events	Control (Non-CCS) Total	Weight	Odds Ratio M-H, Random, 95% CI	Odds Ratio M-H, Random, 95% CI
Antila 2019	11	17	12	14	11.8%	0.31 [0.05, 1.84]	
Chen 2024	17	134	21	131	27.6%	0.76 [0.38, 1.52]	
Kant 2024	16	35	49	70	24.9%	0.36 [0.16, 0.83]	
Laaninen 2016	5	28	14	34	19.1%	0.31 [0.10, 1.01]	
Tarvainen 2020	60	63	54	63	16.6%	3.33 [0.86, 12.95]	
**Total (95% CI)**		**277**		**312**	**100.0%**	**0.61 [0.29, 1.30]**	
Total events	109		150				

Heterogeneity: Tau² = 0.41; Chi² = 9.75, df = 4 (P = 0.04); I² = 59%

Test for overall effect: Z = 1.28 (P = 0.20)

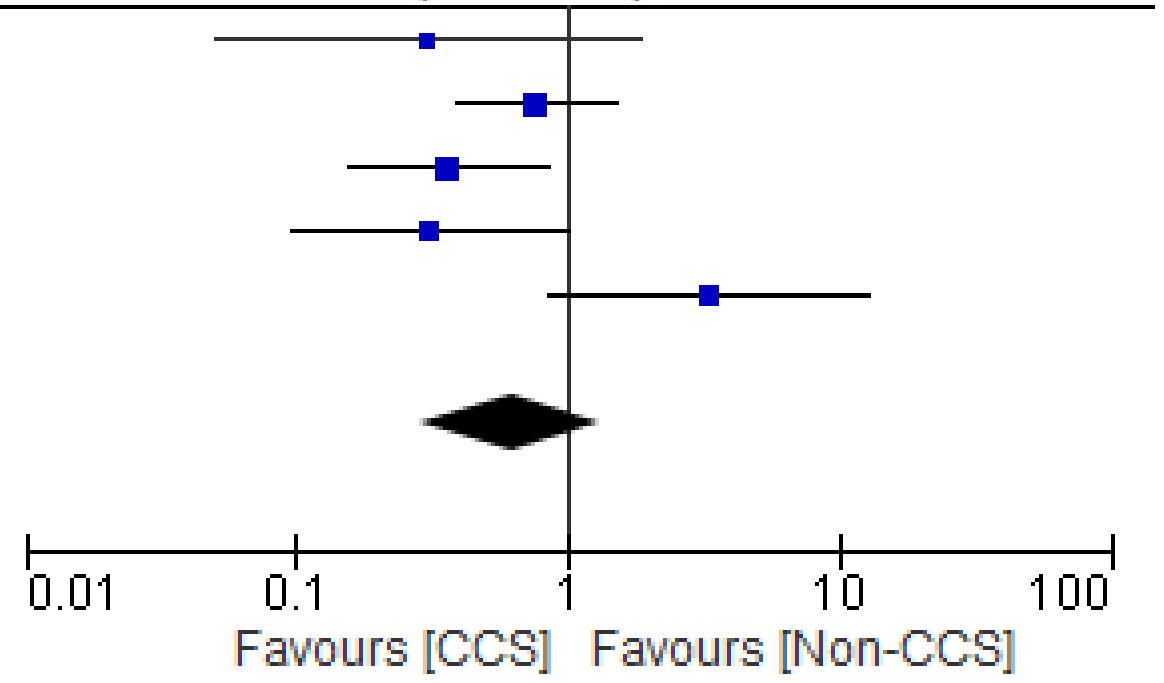

RCT - CCS vs Non-CCS: 5.CR-POPF

Study or Subgroup	Experimental (CCS) Events	Experimental (CCS) Total	Control (Non-CCS) Events	Control (Non-CCS) Total	Weight	Odds Ratio M-H, Random, 95% CI
Antila 2019	1	17	6	14	5.1%	0.08 [0.01, 0.82]
Chen 2024	34	134	41	131	45.2%	0.75 [0.44, 1.28]
Kant 2024	5	35	15	70	18.0%	0.61 [0.20, 1.85]
Laaninen 2016	1	28	4	34	5.2%	0.28 [0.03, 2.64]
Tarvainen 2020	14	63	13	63	26.5%	1.10 [0.47, 2.57]
**Total (95% CI)**		**277**		**312**	**100.0%**	**0.68 [0.40, 1.15]**
Total events	55		79			

Heterogeneity: Tau² = 0.09; Chi² = 5.20, df = 4 (P = 0.27); I² = 23%

Test for overall effect: Z = 1.44 (P = 0.15)

Odds Ratio
M-H, Random, 95% CI

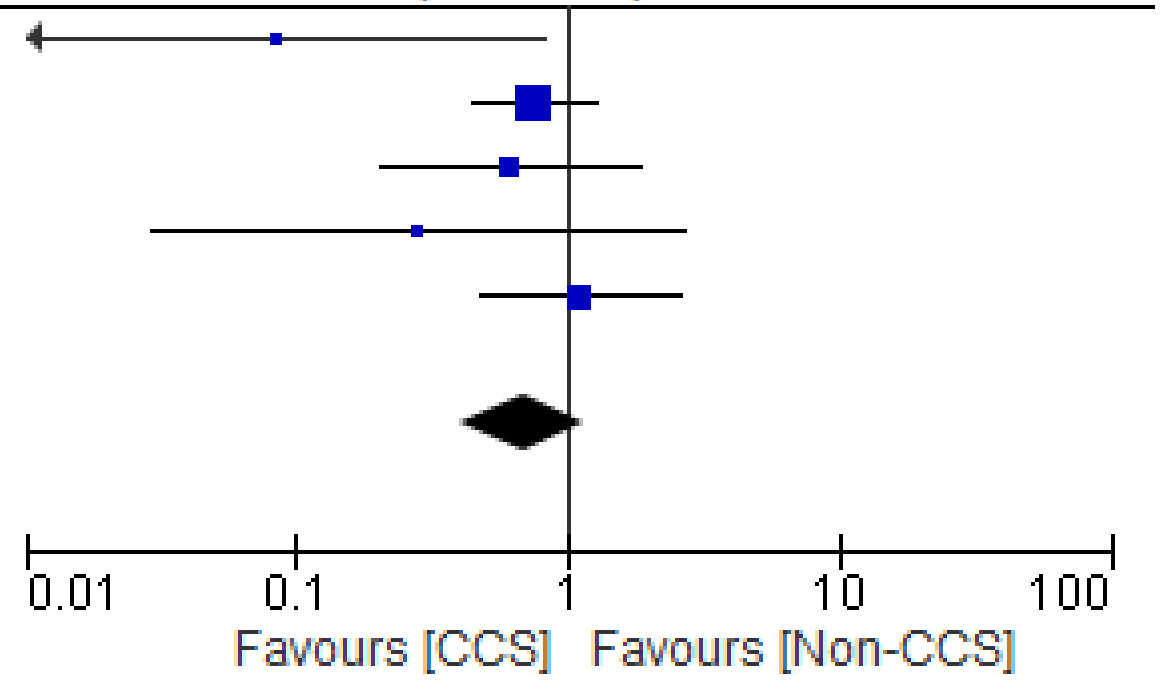

RCT - CCS vs Non-CCS: 6.POPF (biochemical leak and CR-POPF)

Study or Subgroup	Experimental (CCS) Events	Experimental (CCS) Total	Control (Non-CCS) Events	Control (Non-CCS) Total	Weight	Odds Ratio M-H, Random, 95% CI
Antila 2019	7	17	7	14	8.2%	0.70 [0.17, 2.91]
Chen 2024	34	134	41	131	42.3%	0.75 [0.44, 1.28]
Kant 2024	5	35	15	70	13.1%	0.61 [0.20, 1.85]
Laaninen 2016	3	28	10	34	8.4%	0.29 [0.07, 1.18]
Tarvainen 2020	39	63	34	63	27.9%	1.39 [0.68, 2.82]
**Total (95% CI)**		**277**		**312**	**100.0%**	**0.79 [0.52, 1.21]**
Total events	88		107			

Heterogeneity: $Tau^2 = 0.03$; $Chi^2 = 4.66$, df = 4 (P = 0.32); $I^2 = 14\%$

Test for overall effect: Z = 1.08 (P = 0.28)

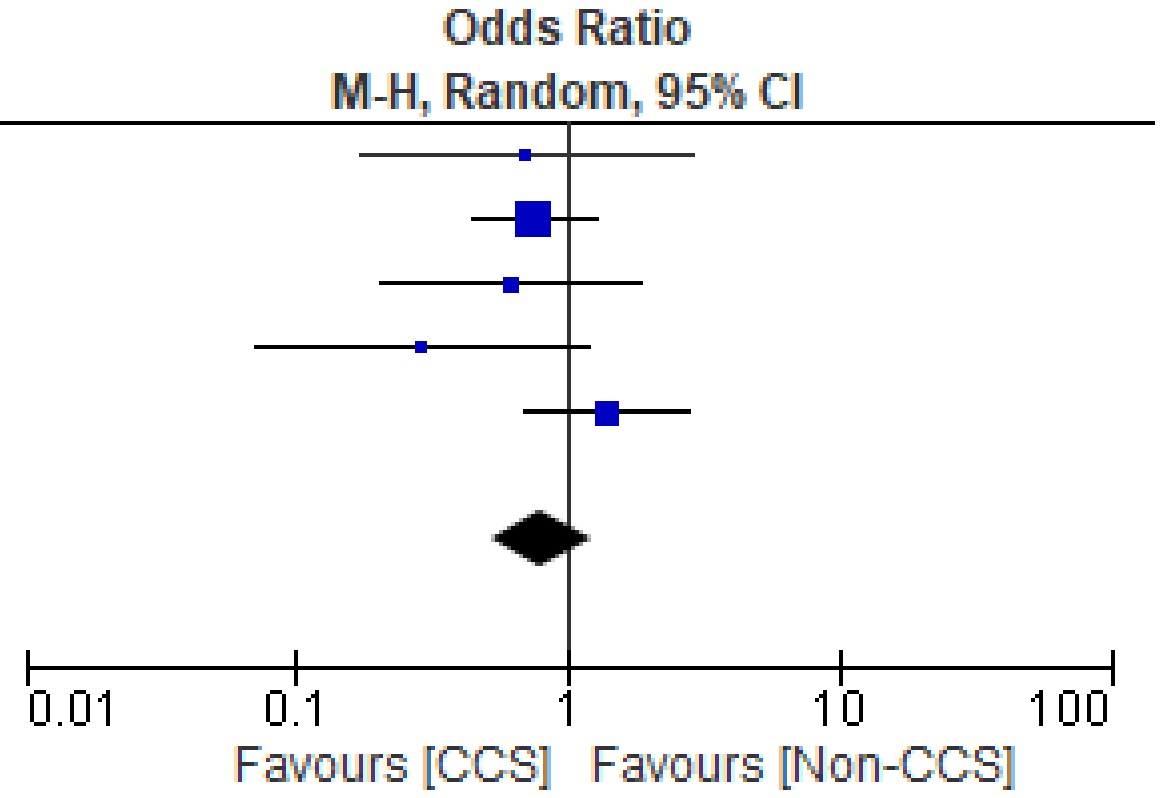

RCT - CCS vs Non-CCS: 7.Bile Leak

	Experimental (CCS)		Control (Non-CCS)			Odds Ratio	Odds Ratio
**Study or Subgroup**	**Events**	**Total**	**Events**	**Total**	**Weight**	**M-H, Random, 95% CI**	**M-H, Random, 95% CI**
Chen 2024	6	134	6	131	69.8%	0.98 [0.31, 3.11]	
Laaninen 2016	1	28	5	34	30.2%	0.21 [0.02, 1.96]	
**Total (95% CI)**		**162**		**165**	**100.0%**	**0.62 [0.16, 2.44]**	
Total events	7		11				

Heterogeneity: Tau² = 0.36; Chi² = 1.44, df = 1 (P = 0.23); I² = 31%
Test for overall effect: Z = 0.69 (P = 0.49)

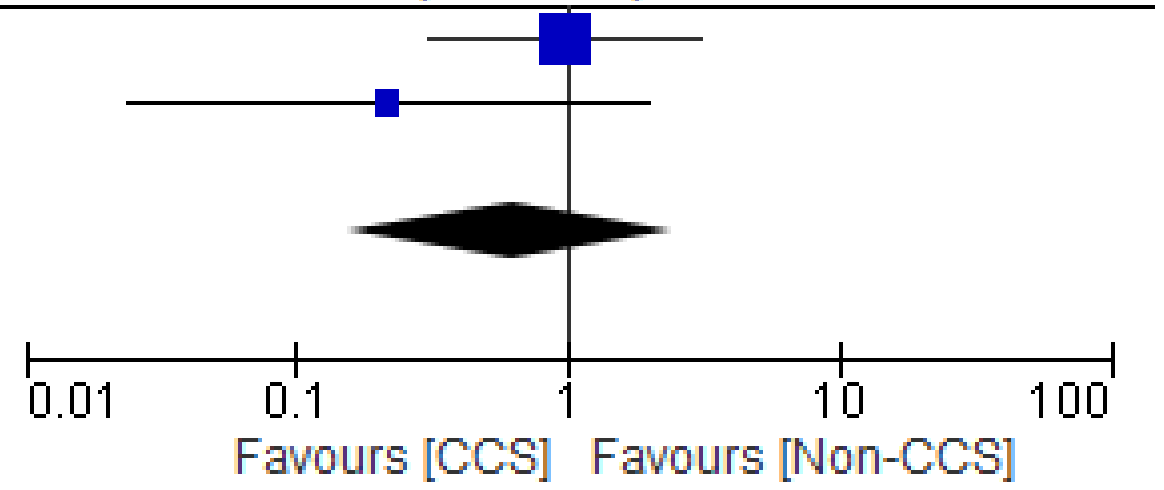

RCT - CCS vs Non-CCS: 8.SSI

Study or Subgroup	Experimental (CCS) Events	Experimental (CCS) Total	Control (Non-CCS) Events	Control (Non-CCS) Total	Weight	Odds Ratio M-H, Random, 95% CI
Antila 2019	1	17	3	14	4.9%	0.23 [0.02, 2.50]
Chen 2024	13	134	22	131	51.6%	0.53 [0.26, 1.11]
Kant 2024	5	35	15	70	22.7%	0.61 [0.20, 1.85]
Laaninen 2016	6	28	11	34	20.8%	0.57 [0.18, 1.81]
**Total (95% CI)**		**214**		**249**	**100.0%**	**0.53 [0.32, 0.91]**
Total events	25		51			

Heterogeneity: Tau² = 0.00; Chi² = 0.55, df = 3 (P = 0.91); I² = 0%

Test for overall effect: Z = 2.33 (P = 0.02)

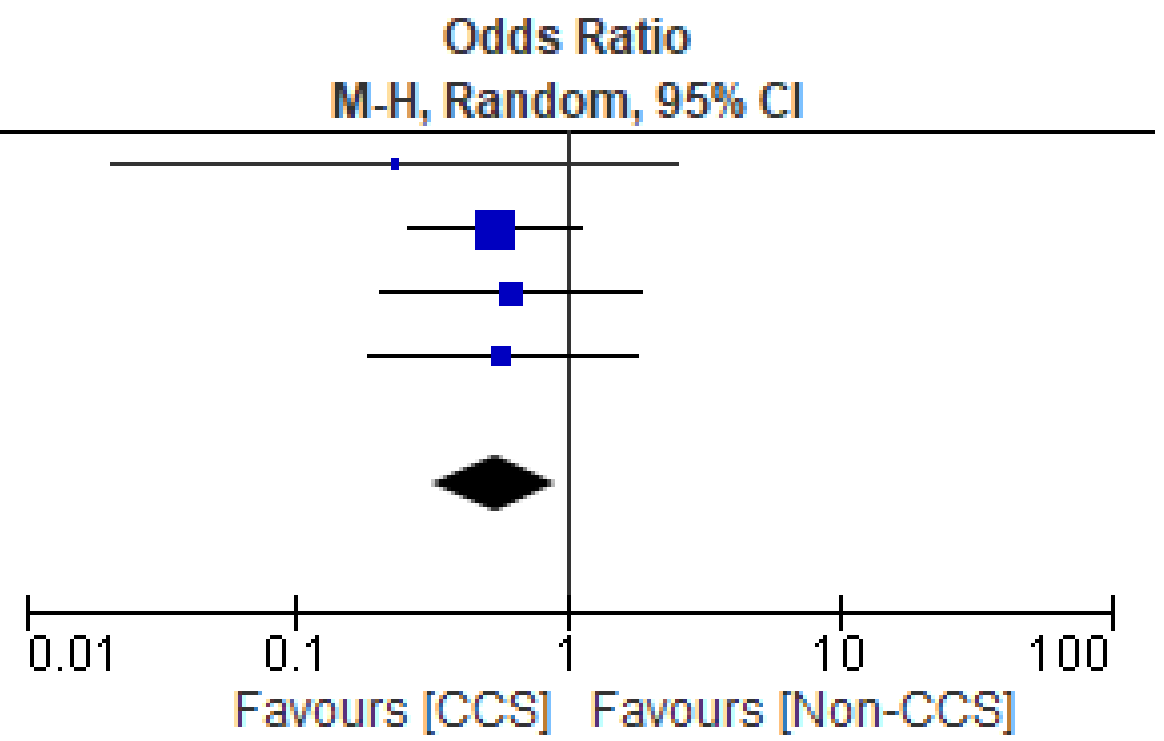

RCT - CCS vs Non-CCS: 9.DGE

Study or Subgroup	Experimental (CCS) Events	Experimental (CCS) Total	Control (Non-CCS) Events	Control (Non-CCS) Total	Weight	Odds Ratio M-H, Random, 95% CI
Antila 2019	1	17	1	14	4.7%	0.81 [0.05, 14.28]
Chen 2024	13	134	17	131	29.5%	0.72 [0.33, 1.55]
Kant 2024	3	35	17	70	16.7%	0.29 [0.08, 1.08]
Laaninen 2016	8	28	15	34	21.4%	0.51 [0.17, 1.47]
Tarvainen 2020	19	63	12	63	27.7%	1.84 [0.80, 4.20]
**Total (95% CI)**		**277**		**312**	**100.0%**	**0.75 [0.39, 1.44]**
Total events	44		62			

Heterogeneity: Tau² = 0.22; Chi² = 6.95, df = 4 (P = 0.14); I² = 42%

Test for overall effect: Z = 0.87 (P = 0.38)

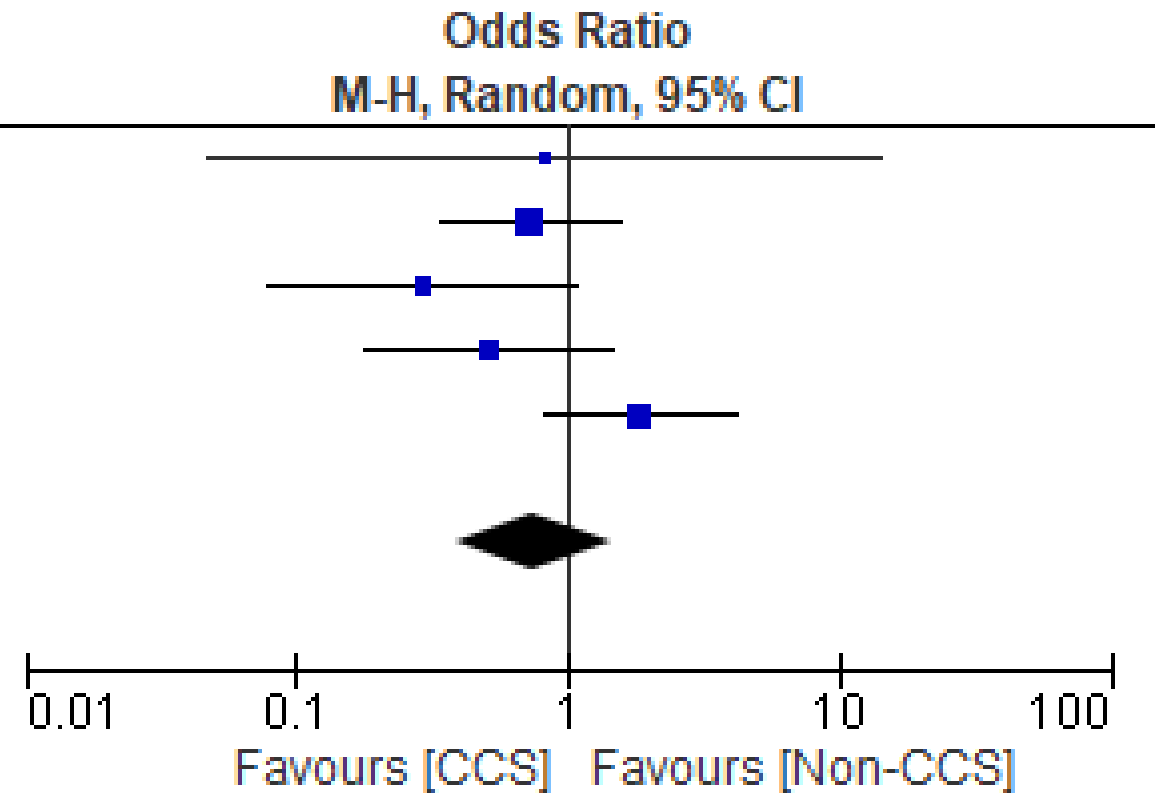

RCT - CCS vs Non-CCS: 10.Reintervention

Study or Subgroup	Experimental (CCS) Events	Experimental (CCS) Total	Control (Non-CCS) Events	Control (Non-CCS) Total	Weight	Odds Ratio M-H, Random, 95% CI
Antila 2019	0	17	1	14	19.1%	0.26 [0.01, 6.82]
Chen 2024	1	134	7	131	46.2%	0.13 [0.02, 1.10]
Kant 2024	1	35	2	70	34.7%	1.00 [0.09, 11.42]
**Total (95% CI)**		**186**		**215**	**100.0%**	**0.30 [0.07, 1.27]**
Total events	2		10			

Heterogeneity: $Tau^2 = 0.00$; $Chi^2 = 1.55$, $df = 2$ ($P = 0.46$); $I^2 = 0\%$

Test for overall effect: $Z = 1.63$ ($P = 0.10$)

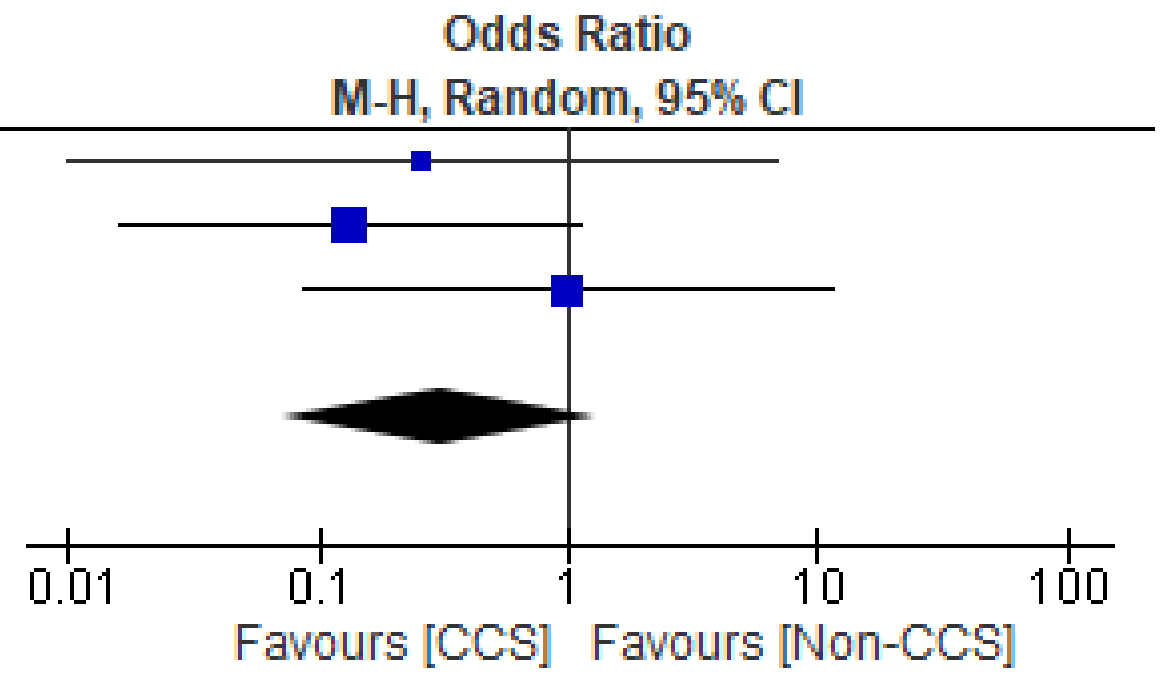

RCT - CCS vs Non-CCS: 11.Readmission

Study or Subgroup	Experimental (CCS) Events	Experimental (CCS) Total	Control (Non-CCS) Events	Control (Non-CCS) Total	Weight	Odds Ratio M-H, Random, 95% CI
Antila 2019	3	17	3	14	18.6%	0.79 [0.13, 4.68]
Kant 2024	2	35	4	70	19.4%	1.00 [0.17, 5.74]
Laaninen 2016	0	28	4	34	6.7%	0.12 [0.01, 2.31]
Tarvainen 2020	10	63	7	63	55.2%	1.51 [0.54, 4.25]
**Total (95% CI)**		**143**		**181**	**100.0%**	**1.04 [0.48, 2.25]**
Total events	15		18			

Heterogeneity: $Tau^2 = 0.00$; $Chi^2 = 2.73$, $df = 3$ ($P = 0.43$); $I^2 = 0\%$

Test for overall effect: $Z = 0.10$ ($P = 0.92$)

Odds Ratio
M-H, Random, 95% CI

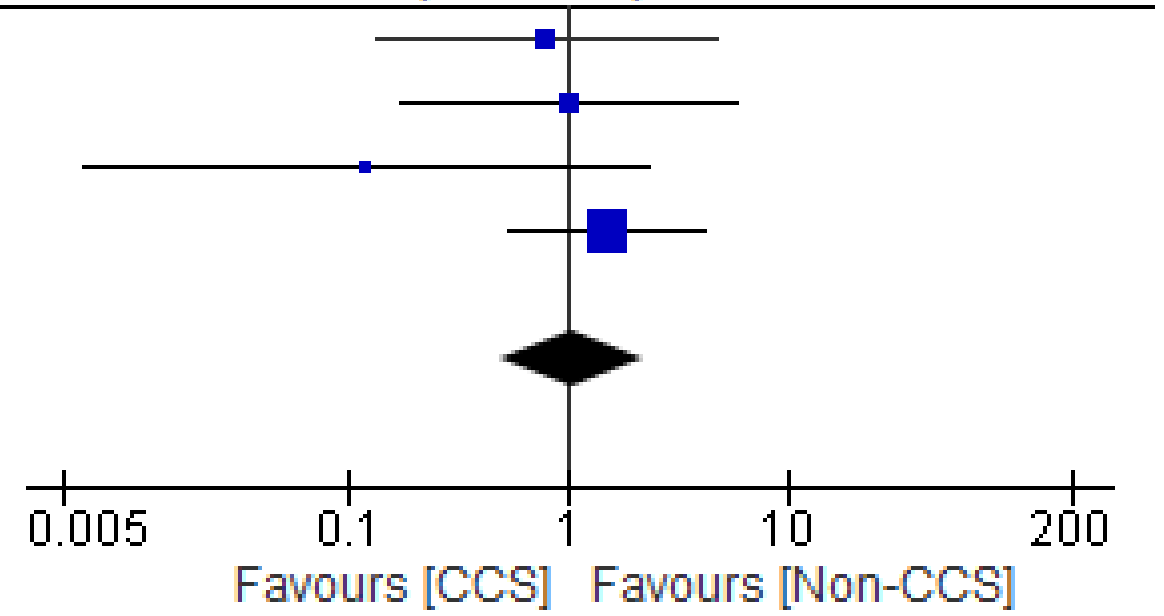

RCT - CCS vs Non-CCS: 12.LOS

	Experimental (CCS)			Control (Non-CCS)				Mean Difference
**Study or Subgroup**	**Mean**	**SD**	**Total**	**Mean**	**SD**	**Total**	**Weight**	**IV, Random, 95% CI**
Antila 2019	8	5.2	17	7	8	14	12.5%	1.00 [-3.87, 5.87]
Chen 2024	16	8.3	134	19	15	131	21.1%	-3.00 [-5.93, -0.07]
Kant 2024	10	4.4	35	11	4.4	70	27.9%	-1.00 [-2.79, 0.79]
Laaninen 2016	9	12.2	28	11	12.2	34	9.2%	-2.00 [-8.10, 4.10]
Tarvainen 2020	10	4.4	63	8	4.4	63	29.3%	2.00 [0.46, 3.54]
**Total (95% CI)**			**277**			**312**	**100.0%**	**-0.38 [-2.54, 1.78]**

Heterogeneity: Tau² = 3.53; Chi² = 12.20, df = 4 (P = 0.02); I² = 67%
Test for overall effect: Z = 0.35 (P = 0.73)

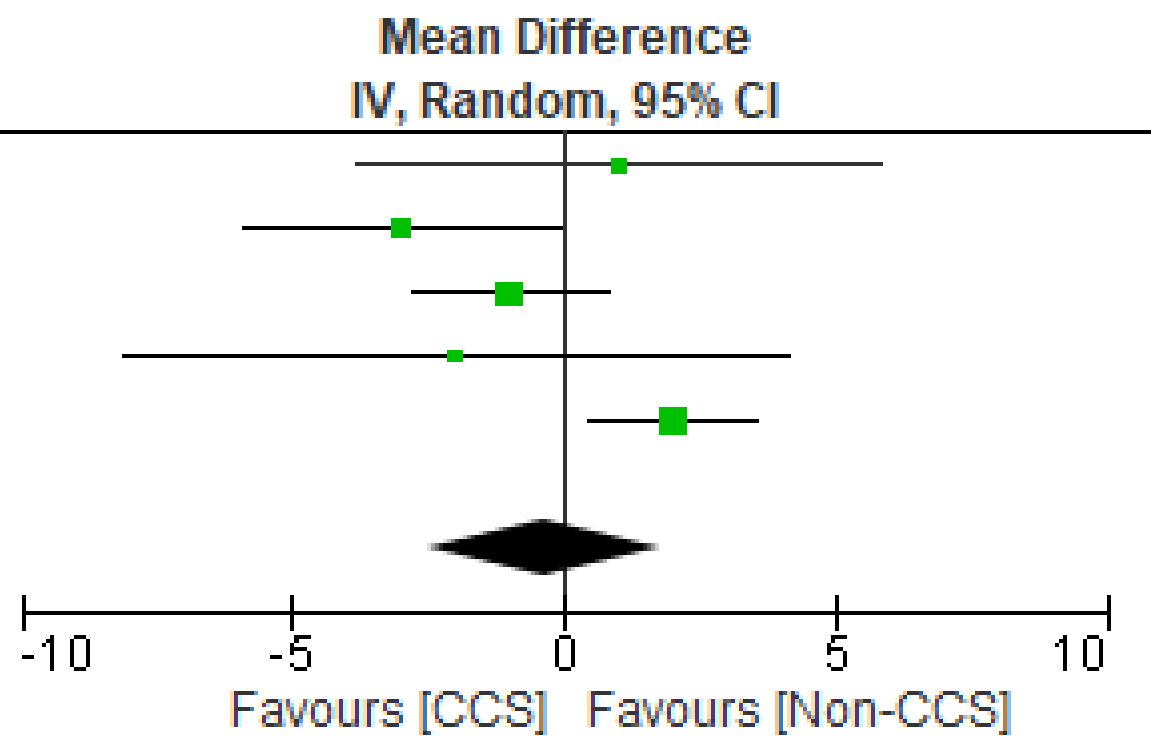

PD - CCS vs Non-CCS: 1.Mortality

Study or Subgroup	Experimental (CCS) Events	Experimental (CCS) Total	Control (Non-CCS) Events	Control (Non-CCS) Total	Weight	Odds Ratio M-H, Random, 95% CI
Chen 2024	3	134	3	131	42.2%	0.98 [0.19, 4.93]
Kant 2024	0	35	1	70	10.6%	0.65 [0.03, 16.43]
Laaninen 2016	0	28	0	34		Not estimable
Sandini 2018	2	117	10	562	47.2%	0.96 [0.21, 4.44]
**Total (95% CI)**		**314**		**797**	**100.0%**	**0.93 [0.32, 2.66]**
Total events	5		14			

Heterogeneity: Tau² = 0.00; Chi² = 0.05, df = 2 (P = 0.97); I² = 0%

Test for overall effect: Z = 0.14 (P = 0.89)

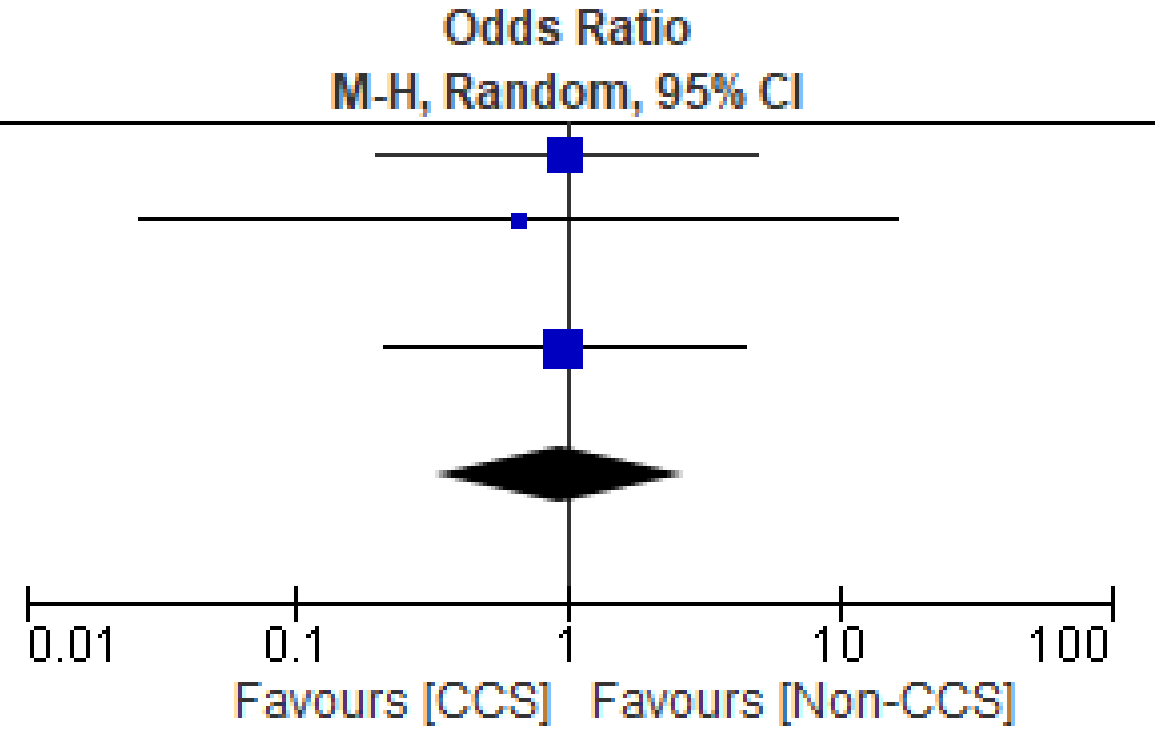

PD - CCS vs Non-CCS: 2.Morbidity mayor

Study or Subgroup	Experimental (CCS) Events	Experimental (CCS) Total	Control (Non-CCS) Events	Control (Non-CCS) Total	Weight	Odds Ratio M-H, Random, 95% CI
Chen 2024	17	134	21	131	30.5%	0.76 [0.38, 1.52]
Kant 2024	3	35	5	70	6.5%	1.22 [0.27, 5.42]
Laaninen 2016	5	28	14	34	10.4%	0.31 [0.10, 1.01]
Sandini 2018	20	117	104	562	52.5%	0.91 [0.54, 1.54]
**Total (95% CI)**		**314**		**797**	**100.0%**	**0.78 [0.54, 1.15]**
Total events	45		144			

Heterogeneity: Tau² = 0.00; Chi² = 2.99, df = 3 (P = 0.39); I² = 0%

Test for overall effect: Z = 1.25 (P = 0.21)

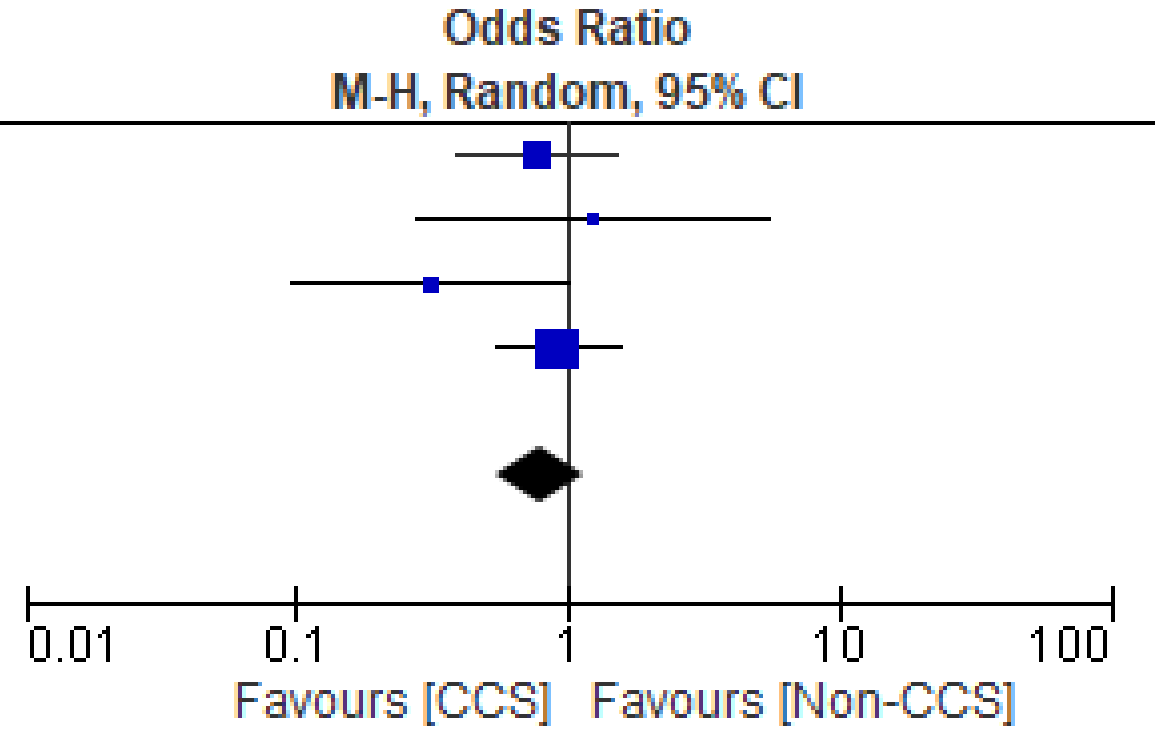

PD - CCS vs Non-CCS: 3.Morbidity minor

	Experimental (CCS)		Control (Non-CCS)			Odds Ratio
Study or Subgroup	Events	Total	Events	Total	Weight	M-H, Random, 95% CI
Chen 2024	17	134	21	131	30.5%	0.76 [0.38, 1.52]
Kant 2024	13	35	45	70	24.6%	0.33 [0.14, 0.76]
Sandini 2018	49	117	249	562	44.9%	0.91 [0.61, 1.36]
**Total (95% CI)**		**286**		**763**	**100.0%**	**0.67 [0.39, 1.15]**
Total events	79		315			

Heterogeneity: Tau² = 0.13; Chi² = 4.54, df = 2 (P = 0.10); I² = 56%
Test for overall effect: Z = 1.44 (P = 0.15)

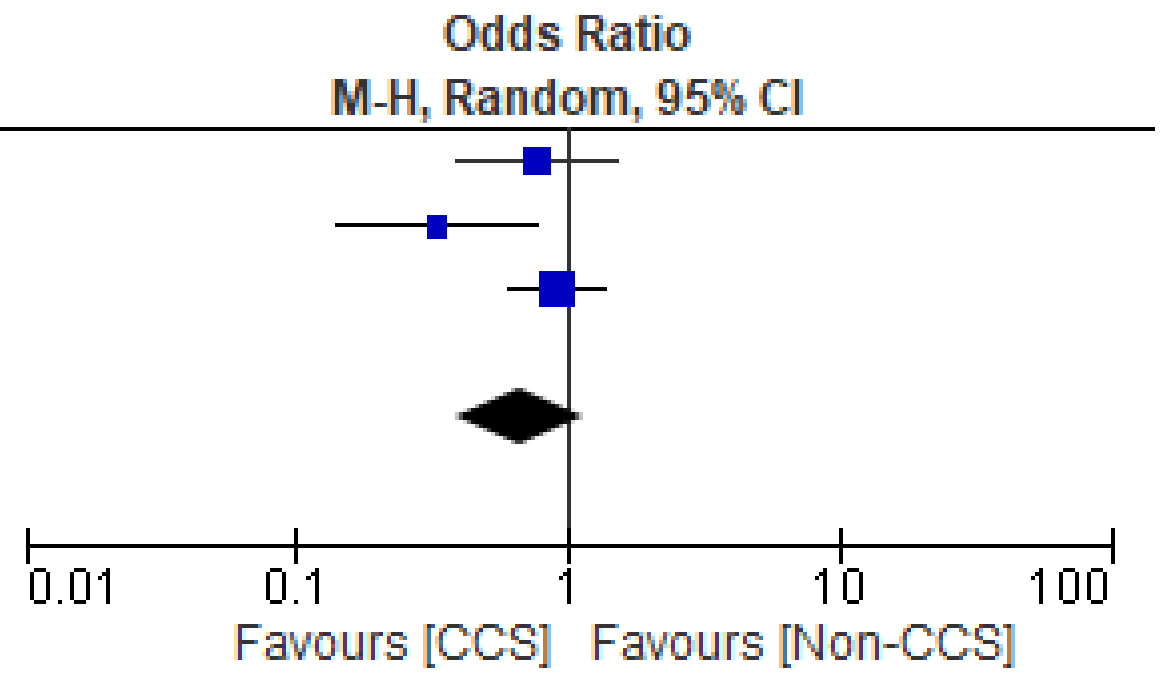

PD - CCS vs Non-CCS: 4.Morbidity global

Study or Subgroup	Experimental (CCS) Events	Experimental (CCS) Total	Control (Non-CCS) Events	Control (Non-CCS) Total	Weight	Odds Ratio M-H, Random, 95% CI
Chen 2024	17	134	21	131	25.9%	0.76 [0.38, 1.52]
Kant 2024	16	35	49	70	20.4%	0.36 [0.16, 0.83]
Laaninen 2016	5	28	14	34	12.3%	0.31 [0.10, 1.01]
Sandini 2018	69	117	353	562	41.5%	0.85 [0.57, 1.28]
**Total (95% CI)**		**314**		**797**	**100.0%**	**0.61 [0.39, 0.98]**
Total events	107		437			

Heterogeneity: $Tau^2 = 0.09$; $Chi^2 = 5.14$, df = 3 (P = 0.16); $I^2 = 42\%$

Test for overall effect: Z = 2.06 (P = 0.04)

Odds Ratio
M-H, Random, 95% CI

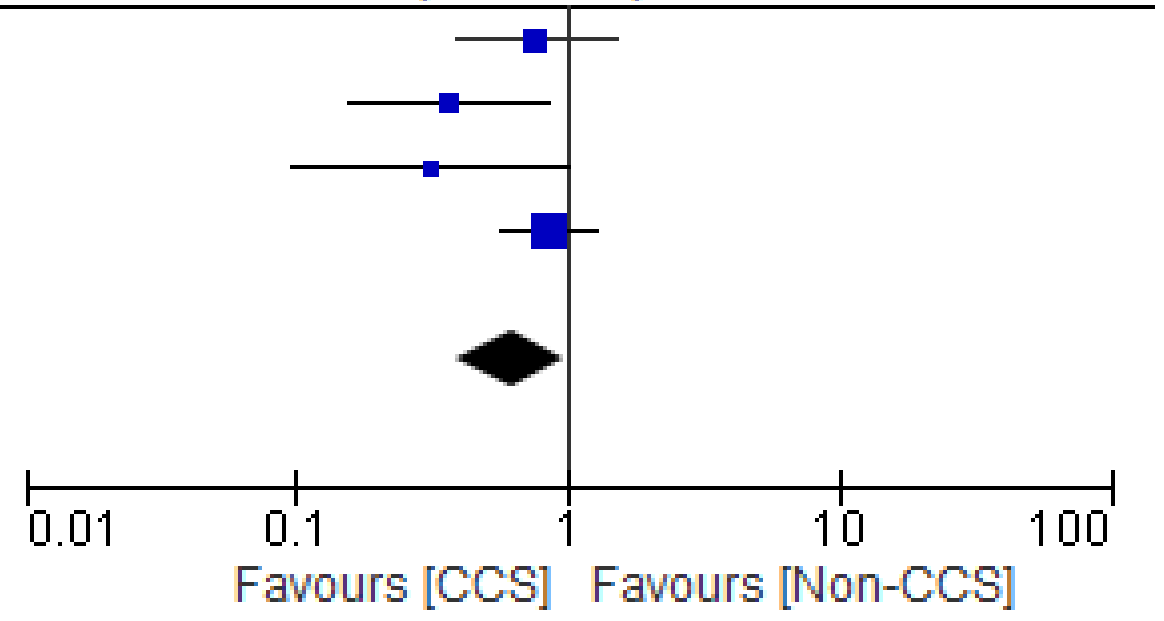

PD - CCS vs Non-CCS: 5.CR-POPF

	Experimental (CCS)		Control (Non-CCS)			Odds Ratio
**Study or Subgroup**	**Events**	**Total**	**Events**	**Total**	**Weight**	**M-H, Random, 95% CI**
Chen 2024	34	134	41	131	60.5%	0.75 [0.44, 1.28]
Kant 2024	5	35	15	70	14.2%	0.61 [0.20, 1.85]
Laaninen 2016	1	28	4	34	3.4%	0.28 [0.03, 2.64]
Sandini 2018	6	117	34	562	21.9%	0.84 [0.34, 2.05]
**Total (95% CI)**		**314**		**797**	**100.0%**	**0.72 [0.47, 1.09]**
Total events	46		94			

Heterogeneity: Tau² = 0.00; Chi² = 0.90, df = 3 (P = 0.82); I² = 0%

Test for overall effect: Z = 1.55 (P = 0.12)

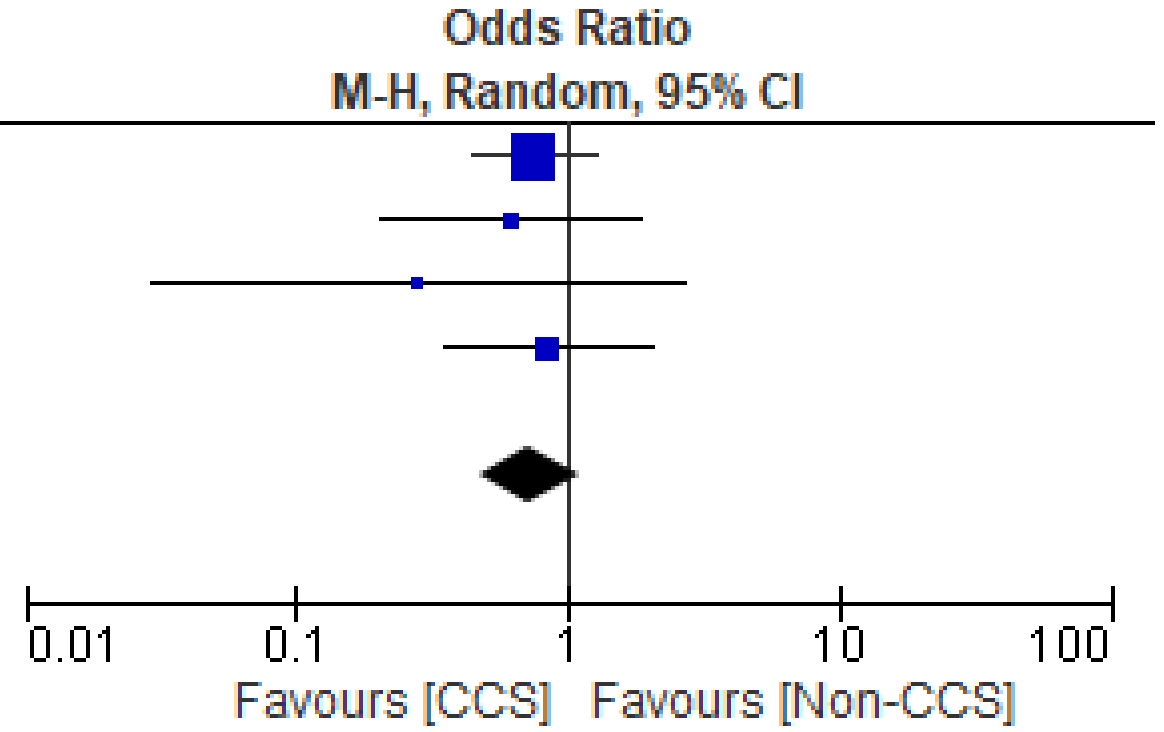

PD - CCS vs Non-CCS: 6.POPF (biochemical leak and CR-POPF)

	Experimental (CCS)		Control (Non-CCS)			Odds Ratio
Study or Subgroup	Events	Total	Events	Total	Weight	M-H, Random, 95% CI
Chen 2024	34	134	41	131	57.4%	0.75 [0.44, 1.28]
Kant 2024	5	35	15	70	13.5%	0.61 [0.20, 1.85]
Laaninen 2016	3	28	10	34	8.3%	0.29 [0.07, 1.18]
Sandini 2018	6	117	34	562	20.8%	0.84 [0.34, 2.05]
**Total (95% CI)**		**314**		**797**	**100.0%**	**0.69 [0.46, 1.03]**
Total events	48		100			

Heterogeneity: Tau² = 0.00; Chi² = 1.80, df = 3 (P = 0.62); I² = 0%

Test for overall effect: Z = 1.81 (P = 0.07)

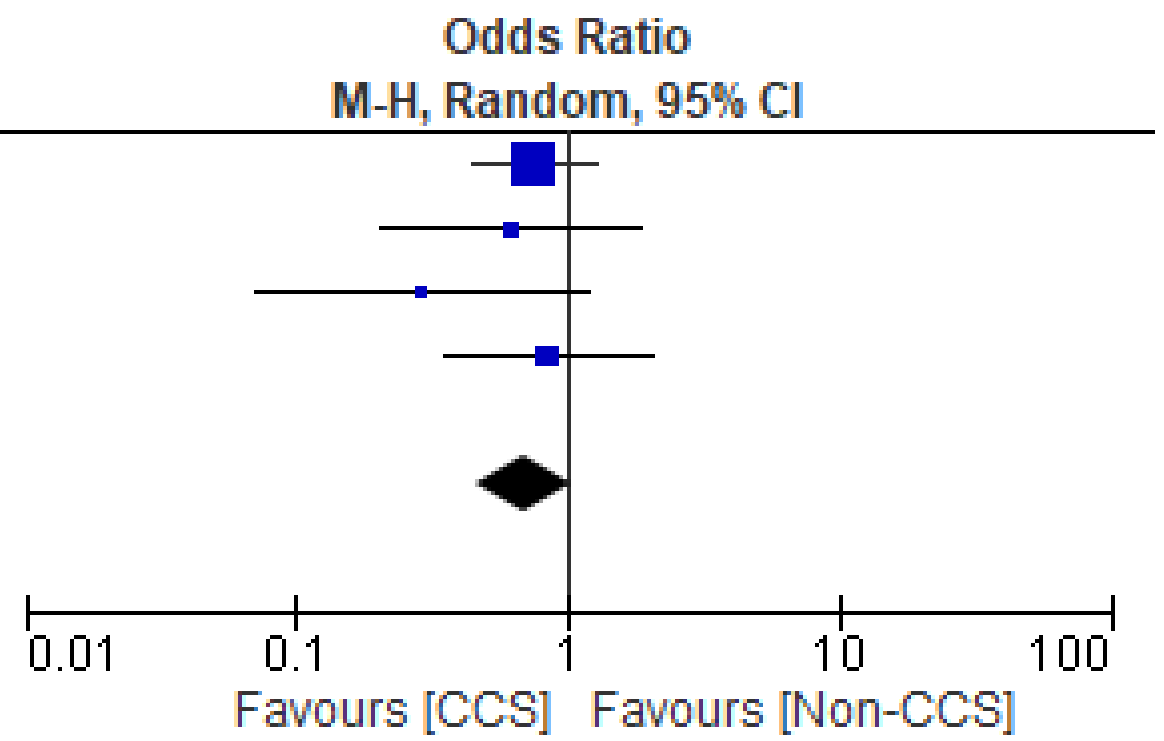

PD - CCS vs Non-CCS: 7.Bile Leak

Study or Subgroup	Experimental (CCS) Events	Experimental (CCS) Total	Control (Non-CCS) Events	Control (Non-CCS) Total	Weight	Odds Ratio M-H, Random, 95% CI	Odds Ratio M-H, Random, 95% CI
Chen 2024	6	134	6	131	39.0%	0.98 [0.31, 3.11]	
Laaninen 2016	1	28	5	34	11.9%	0.21 [0.02, 1.96]	
Sandini 2018	5	117	18	562	49.1%	1.35 [0.49, 3.71]	
**Total (95% CI)**		**279**		**727**	**100.0%**	**0.96 [0.44, 2.08]**	
Total events	12		29				

Heterogeneity: Tau² = 0.06; Chi² = 2.24, df = 2 (P = 0.33); I² = 11%

Test for overall effect: Z = 0.11 (P = 0.91)

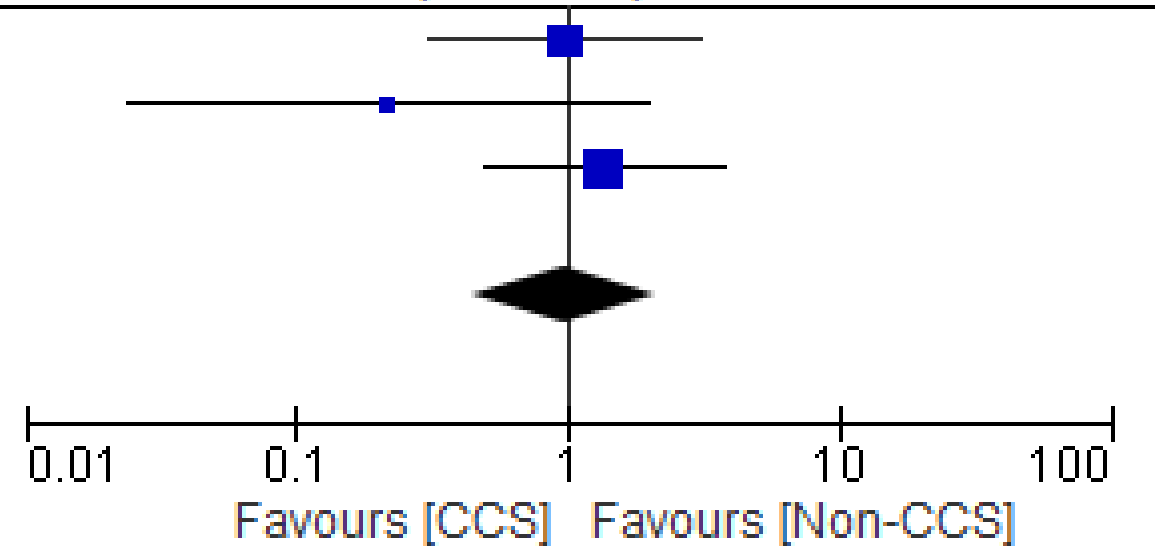

PD - CCS vs Non-CCS: 8.SSI

Study or Subgroup	Experimental (CCS) Events	Experimental (CCS) Total	Control (Non-CCS) Events	Control (Non-CCS) Total	Weight	Odds Ratio M-H, Random, 95% CI
Chen 2024	13	134	22	131	25.0%	0.53 [0.26, 1.11]
Kant 2024	5	35	15	70	11.0%	0.61 [0.20, 1.85]
Laaninen 2016	6	28	11	34	10.1%	0.57 [0.18, 1.81]
Sandini 2018	22	117	160	562	54.0%	0.58 [0.35, 0.96]
**Total (95% CI)**		**314**		**797**	**100.0%**	**0.57 [0.40, 0.82]**
Total events	46		208			

Heterogeneity: $Tau^2 = 0.00$; $Chi^2 = 0.06$, df = 3 (P = 1.00); $I^2 = 0\%$

Test for overall effect: Z = 3.00 (P = 0.003)

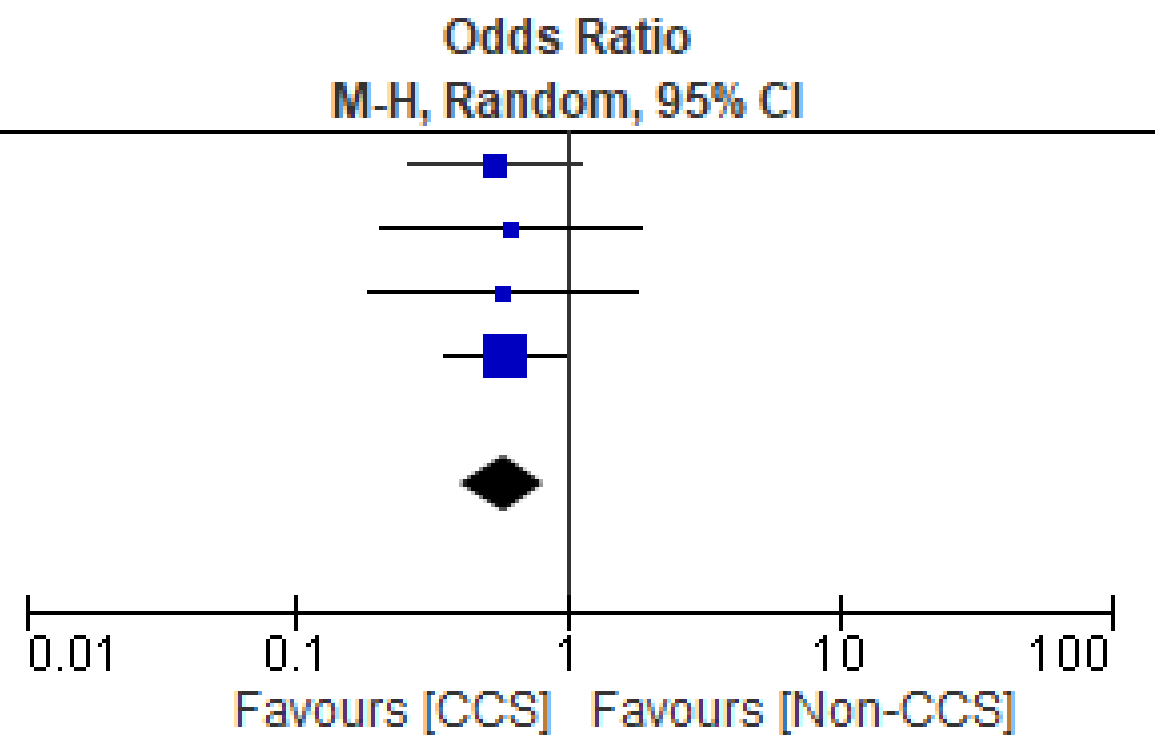

PD - CCS vs Non-CCS: 9.DGE

	Experimental (CCS)		Control (Non-CCS)			Odds Ratio
**Study or Subgroup**	**Events**	**Total**	**Events**	**Total**	**Weight**	**M-H, Random, 95% CI**
Chen 2024	13	134	17	131	27.7%	0.72 [0.33, 1.55]
Kant 2024	3	35	17	70	11.3%	0.29 [0.08, 1.08]
Laaninen 2016	8	28	15	34	16.2%	0.51 [0.17, 1.47]
Sandini 2018	19	117	90	562	44.8%	1.02 [0.59, 1.75]
**Total (95% CI)**		**314**		**797**	**100.0%**	**0.72 [0.45, 1.14]**
Total events	43		139			

Heterogeneity: Tau² = 0.05; Chi² = 3.77, df = 3 (P = 0.29); I² = 20%
Test for overall effect: Z = 1.41 (P = 0.16)

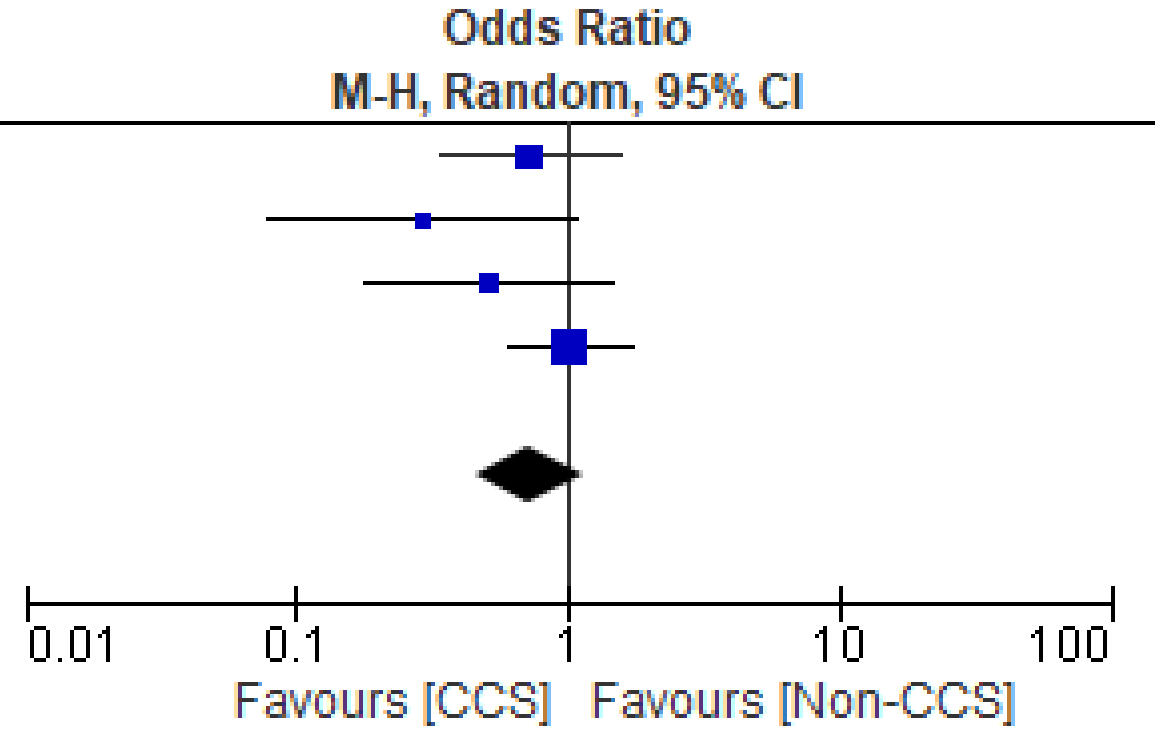

PD - CCS vs Non-CCS: 10.Reintervention

Study or Subgroup	Experimental (CCS) Events	Experimental (CCS) Total	Control (Non-CCS) Events	Control (Non-CCS) Total	Weight	Odds Ratio M-H, Random, 95% CI
Chen 2024	1	134	7	131	26.5%	0.13 [0.02, 1.10]
Kant 2024	1	35	2	70	21.1%	1.00 [0.09, 11.42]
Sandini 2018	3	117	15	562	52.3%	0.96 [0.27, 3.37]
**Total (95% CI)**		**286**		**763**	**100.0%**	**0.57 [0.17, 1.99]**
Total events	5		24			

Heterogeneity: Tau² = 0.36; Chi² = 2.77, df = 2 (P = 0.25); I² = 28%
Test for overall effect: Z = 0.88 (P = 0.38)

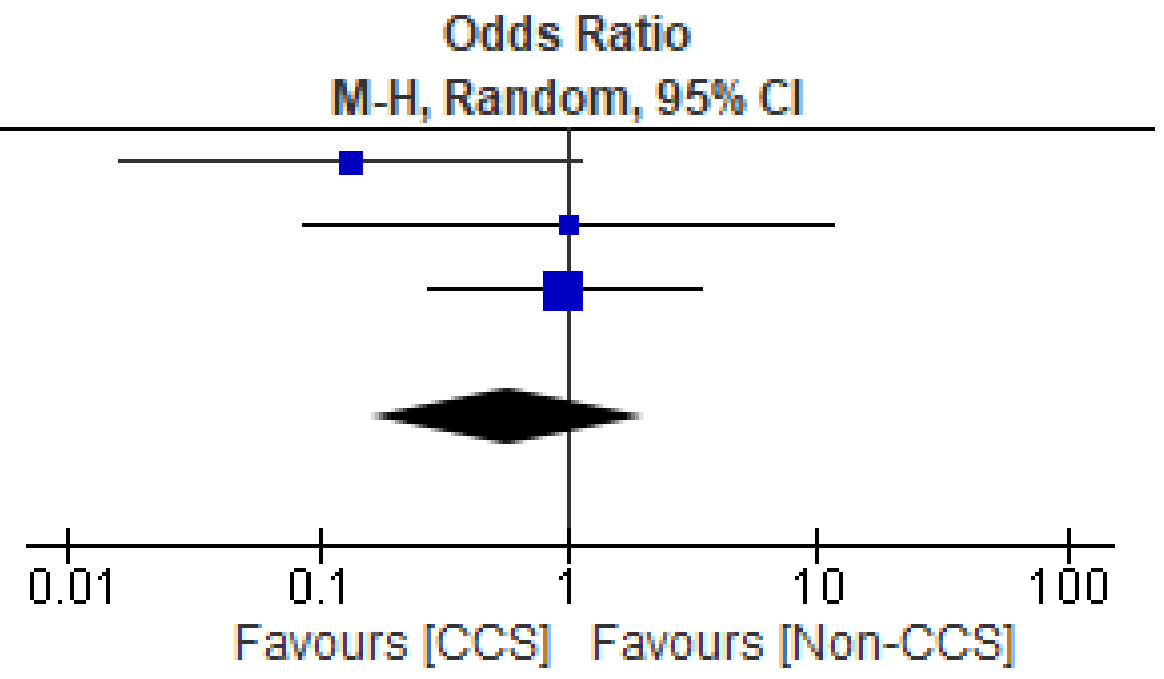

PD - CCS vs Non-CCS: 11.Readmission

Study or Subgroup	Experimental (CCS) Events	Experimental (CCS) Total	Control (Non-CCS) Events	Control (Non-CCS) Total	Weight	Odds Ratio M-H, Random, 95% CI	Odds Ratio M-H, Random, 95% CI
Kant 2024	2	35	4	70	8.3%	1.00 [0.17, 5.74]	
Laaninen 2016	0	28	4	34	2.9%	0.12 [0.01, 2.31]	
Sandini 2018	19	117	104	562	88.8%	0.85 [0.50, 1.46]	
**Total (95% CI)**		**180**		**666**	**100.0%**	**0.82 [0.49, 1.35]**	
Total events	21		112				

Heterogeneity: Tau² = 0.00; Chi² = 1.73, df = 2 (P = 0.42); I² = 0%

Test for overall effect: Z = 0.78 (P = 0.43)

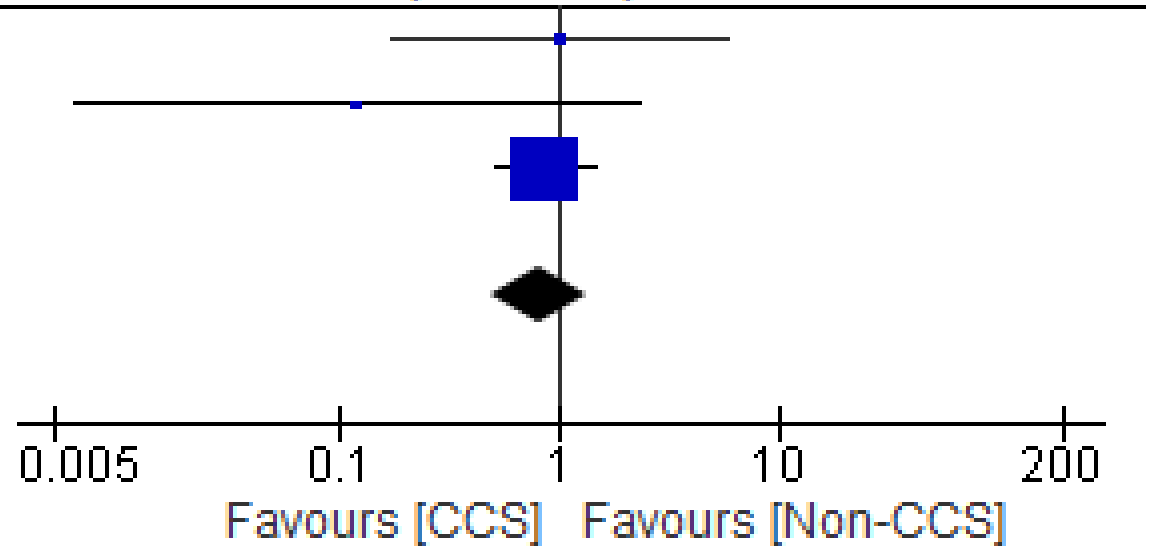

PD - CCS vs Non-CCS: 12.LOS

	Experimental (CCS)			Control (Non-CCS)				Mean Difference
**Study or Subgroup**	**Mean**	**SD**	**Total**	**Mean**	**SD**	**Total**	**Weight**	**IV, Random, 95% CI**
Chen 2024	16	8.3	134	19	15	131	25.5%	-3.00 [-5.93, -0.07]
Kant 2024	10	4.4	35	11	4.4	70	68.6%	-1.00 [-2.79, 0.79]
Laaninen 2016	9	12.2	28	11	12.2	34	5.9%	-2.00 [-8.10, 4.10]
**Total (95% CI)**			**197**			**235**	**100.0%**	**-1.57 [-3.05, -0.09]**

Heterogeneity: Tau² = 0.00; Chi² = 1.33, df = 2 (P = 0.52); I² = 0%

Test for overall effect: Z = 2.08 (P = 0.04)

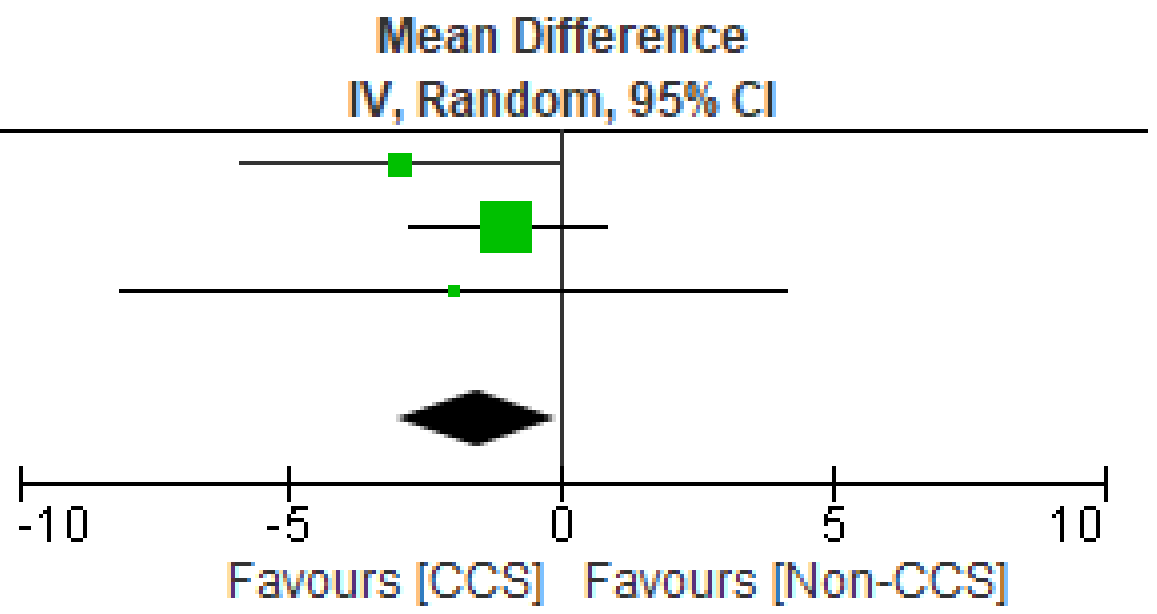

Hydrocortisone - CCS vs Non-CCS: 1.Mortality

	Experimental (CCS)		Control (Non-CCS)			Odds Ratio
**Study or Subgroup**	**Events**	**Total**	**Events**	**Total**	**Weight**	**M-H, Random, 95% CI**
Antila 2019	0	17	0	14		Not estimable
Kant 2024	0	35	1	70	36.1%	0.65 [0.03, 16.43]
Laaninen 2016	0	28	0	34		Not estimable
Tarvainen 2020	2	63	1	63	63.9%	2.03 [0.18, 23.01]
**Total (95% CI)**		**143**		**181**	**100.0%**	**1.35 [0.19, 9.37]**
Total events	2		2			

Heterogeneity: Tau² = 0.00; Chi² = 0.30, df = 1 (P = 0.58); I² = 0%
Test for overall effect: Z = 0.30 (P = 0.76)

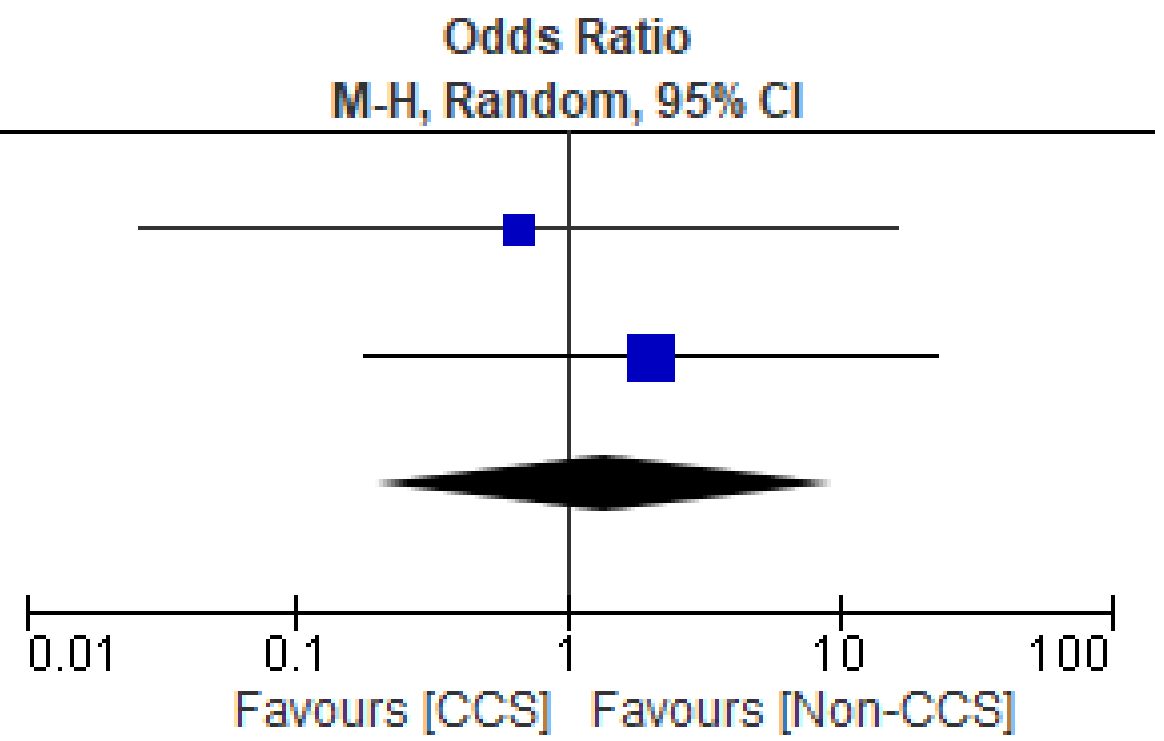

Hydrocortisone - CCS vs Non-CCS: 2.Morbidity mayor

	Experimental (CCS)		Control (Non-CCS)			Odds Ratio
Study or Subgroup	Events	Total	Events	Total	Weight	M-H, Random, 95% CI
Antila 2019	1	17	3	14	14.4%	0.23 [0.02, 2.50]
Kant 2024	3	35	5	70	24.8%	1.22 [0.27, 5.42]
Laaninen 2016	5	28	14	34	30.0%	0.31 [0.10, 1.01]
Tarvainen 2020	10	63	5	63	30.8%	2.19 [0.70, 6.82]
**Total (95% CI)**		**143**		**181**	**100.0%**	**0.76 [0.26, 2.27]**
Total events	19		27			

Heterogeneity: Tau² = 0.67; Chi² = 6.82, df = 3 (P = 0.08); I² = 56%
Test for overall effect: Z = 0.49 (P = 0.63)

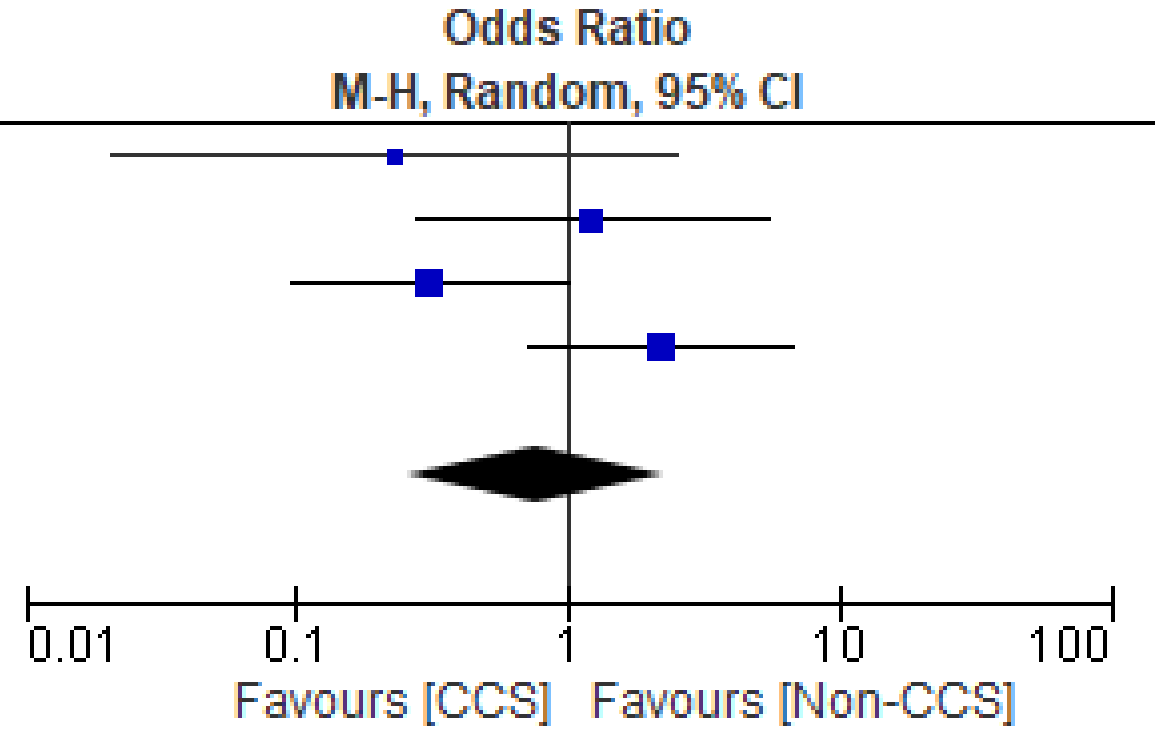

Hydrocortisone - CCS vs Non-CCS: 3.Morbidity minor

Study or Subgroup	Experimental (CCS) Events	Experimental (CCS) Total	Control (Non-CCS) Events	Control (Non-CCS) Total	Weight	Odds Ratio M-H, Random, 95% CI
Antila 2019	10	17	9	14	21.6%	0.79 [0.18, 3.41]
Kant 2024	13	35	45	70	39.4%	0.33 [0.14, 0.76]
Tarvainen 2020	50	63	49	63	39.0%	1.10 [0.47, 2.57]
**Total (95% CI)**		**115**		**147**	**100.0%**	**0.64 [0.28, 1.45]**
Total events	73		103			

Heterogeneity: Tau² = 0.27; Chi² = 4.04, df = 2 (P = 0.13); I² = 50%

Test for overall effect: Z = 1.07 (P = 0.28)

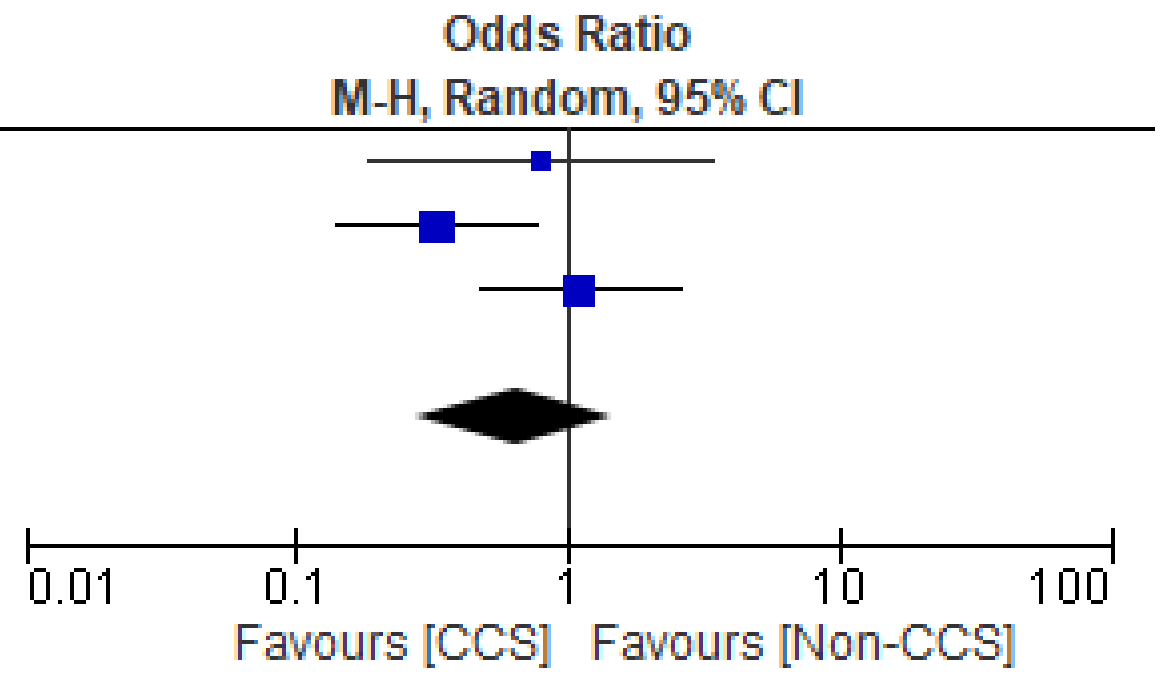

Hydrocortisone - CCS vs Non-CCS: 4.Morbidity global

	Experimental (CCS)		Control (Non-CCS)			Odds Ratio
**Study or Subgroup**	**Events**	**Total**	**Events**	**Total**	**Weight**	**M-H, Random, 95% CI**
Antila 2019	11	17	12	14	18.6%	0.31 [0.05, 1.84]
Kant 2024	16	35	49	70	31.2%	0.36 [0.16, 0.83]
Laaninen 2016	5	28	14	34	26.3%	0.31 [0.10, 1.01]
Tarvainen 2020	60	63	54	63	23.9%	3.33 [0.86, 12.95]
**Total (95% CI)**		**143**		**181**	**100.0%**	**0.57 [0.19, 1.68]**
Total events	92		129			

Heterogeneity: $Tau^2 = 0.78$; $Chi^2 = 9.06$, df = 3 (P = 0.03); $I^2 = 67\%$

Test for overall effect: Z = 1.02 (P = 0.31)

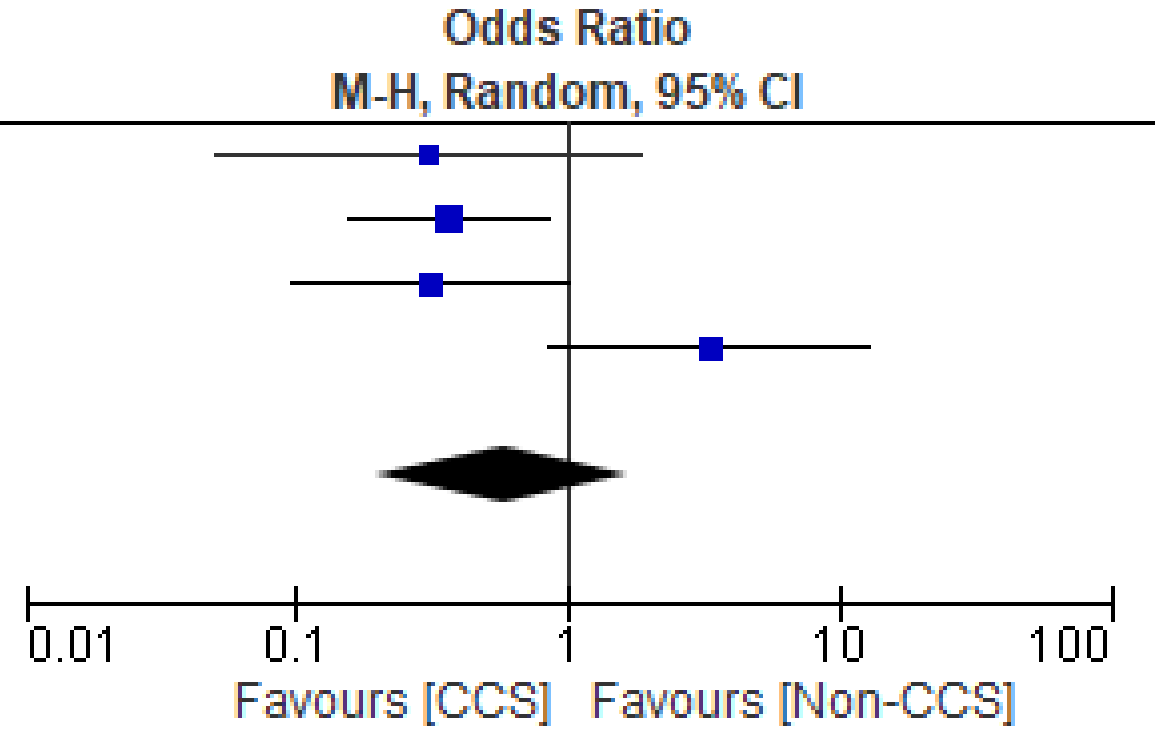

Hydrocortisone - CCS vs Non-CCS: 5.CR-POPF

	Experimental (CCS)		Control (Non-CCS)			Odds Ratio
Study or Subgroup	Events	Total	Events	Total	Weight	M-H, Random, 95% CI
Antila 2019	1	17	6	14	13.1%	0.08 [0.01, 0.82]
Kant 2024	5	35	15	70	32.9%	0.61 [0.20, 1.85]
Laaninen 2016	1	28	4	34	13.3%	0.28 [0.03, 2.64]
Tarvainen 2020	14	63	13	63	40.7%	1.10 [0.47, 2.57]
**Total (95% CI)**		**143**		**181**	**100.0%**	**0.54 [0.21, 1.36]**
Total events	21		38			

Heterogeneity: Tau² = 0.36; Chi² = 5.18, df = 3 (P = 0.16); I² = 42%
Test for overall effect: Z = 1.31 (P = 0.19)

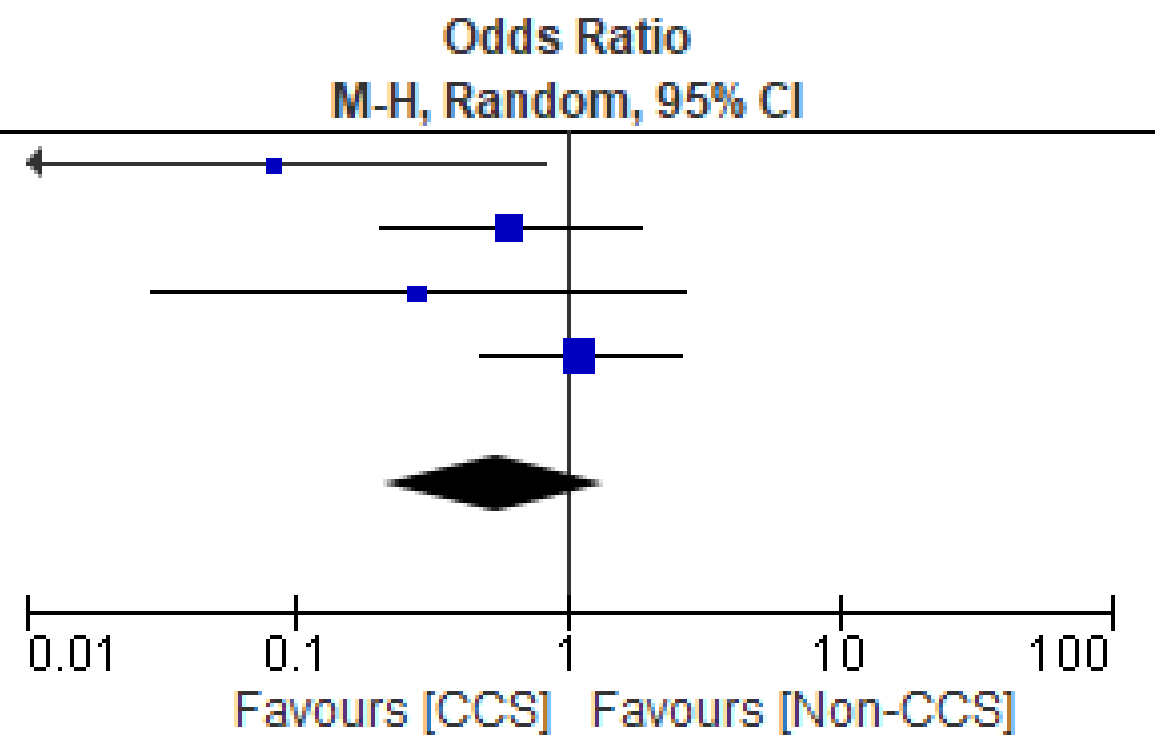

Hydrocortisone - CCS vs Non-CCS: 6.POPF (biochemical leak and CR-POPF)

	Experimental (CCS)		Control (Non-CCS)			Odds Ratio	Odds Ratio
**Study or Subgroup**	**Events**	**Total**	**Events**	**Total**	**Weight**	**M-H, Random, 95% CI**	**M-H, Random, 95% CI**
Antila 2019	7	17	7	14	17.2%	0.70 [0.17, 2.91]	
Kant 2024	5	35	15	70	24.7%	0.61 [0.20, 1.85]	
Laaninen 2016	3	28	10	34	17.5%	0.29 [0.07, 1.18]	
Tarvainen 2020	39	63	34	63	40.6%	1.39 [0.68, 2.82]	
**Total (95% CI)**		**143**		**181**	**100.0%**	**0.76 [0.39, 1.50]**	
Total events	54		66				

Heterogeneity: Tau² = 0.16; Chi² = 4.52, df = 3 (P = 0.21); I² = 34%

Test for overall effect: Z = 0.78 (P = 0.43)

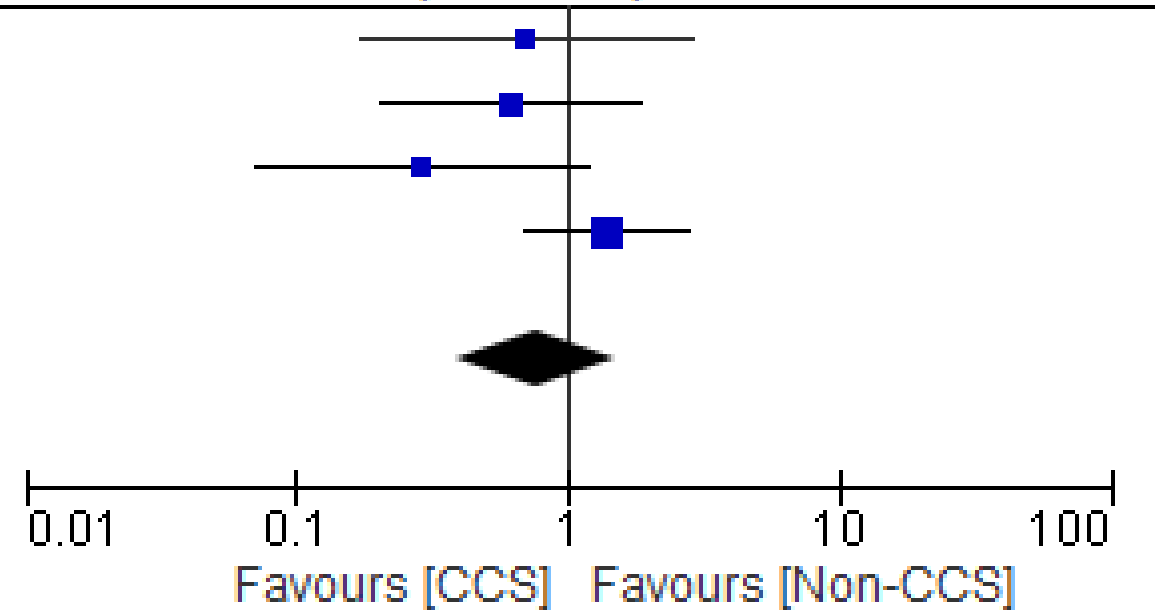

Hydrocortisone - CCS vs Non-CCS: 7.Bile Leak

Study or Subgroup	Experimental (CCS) Events	Experimental (CCS) Total	Control (Non-CCS) Events	Control (Non-CCS) Total	Weight	Odds Ratio M-H, Random, 95% CI
Laaninen 2016	1	28	5	34	100.0%	0.21 [0.02, 1.96]
**Total (95% CI)**		**28**		**34**	**100.0%**	**0.21 [0.02, 1.96]**
Total events	1		5			
Heterogeneity: Not applicable						
Test for overall effect: Z = 1.36 (P = 0.17)						

Odds Ratio
M-H, Random, 95% CI

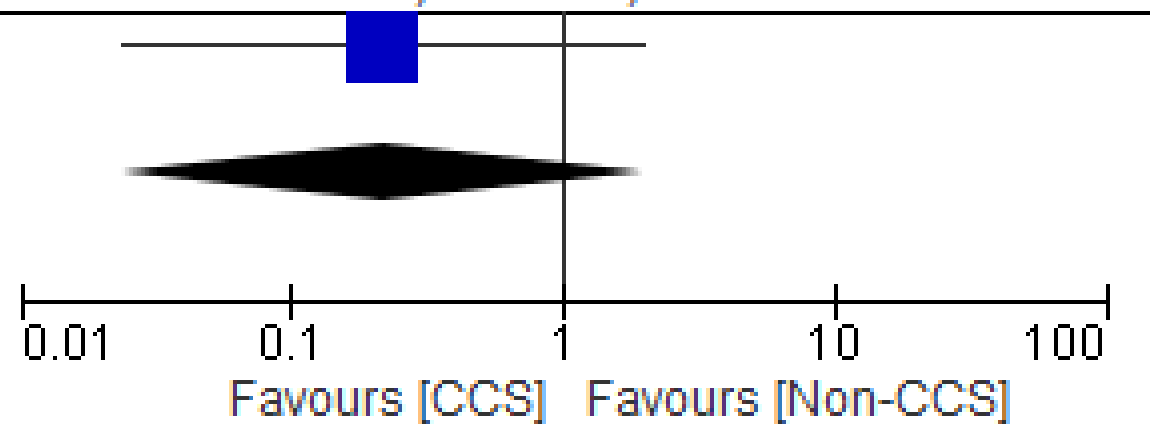

Hydrocortisone - CCS vs Non-CCS: 8.SSI

Study or Subgroup	Experimental (CCS) Events	Experimental (CCS) Total	Control (Non-CCS) Events	Control (Non-CCS) Total	Weight	Odds Ratio M-H, Random, 95% CI
Antila 2019	1	17	3	14	10.0%	0.23 [0.02, 2.50]
Kant 2024	5	35	15	70	46.9%	0.61 [0.20, 1.85]
Laaninen 2016	6	28	11	34	43.1%	0.57 [0.18, 1.81]
**Total (95% CI)**		**80**		**118**	**100.0%**	**0.54 [0.25, 1.15]**
Total events	12		29			

Heterogeneity: Tau² = 0.00; Chi² = 0.55, df = 2 (P = 0.76); I² = 0%

Test for overall effect: Z = 1.61 (P = 0.11)

Odds Ratio
M-H, Random, 95% CI

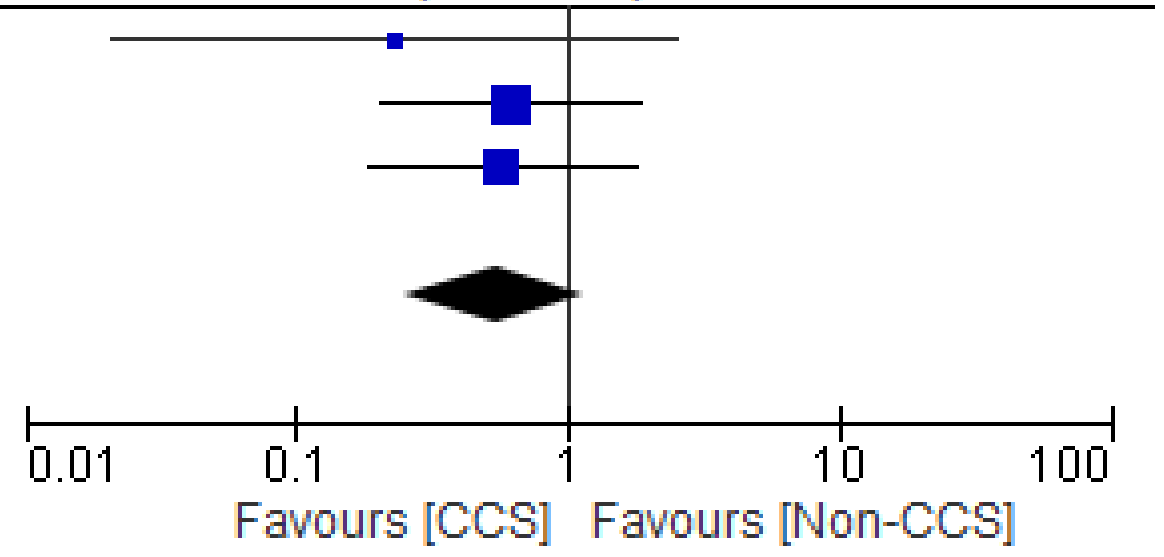

Hydrocortisone - CCS vs Non-CCS: 9.DGE

	Experimental (CCS)		Control (Non-CCS)			Odds Ratio	Odds Ratio
**Study or Subgroup**	**Events**	**Total**	**Events**	**Total**	**Weight**	**M-H, Random, 95% CI**	**M-H, Random, 95% CI**
Antila 2019	1	17	1	14	9.1%	0.81 [0.05, 14.28]	
Kant 2024	3	35	17	70	25.4%	0.29 [0.08, 1.08]	
Laaninen 2016	8	28	15	34	30.2%	0.51 [0.17, 1.47]	
Tarvainen 2020	19	63	12	63	35.3%	1.84 [0.80, 4.20]	
**Total (95% CI)**		**143**		**181**	**100.0%**	**0.72 [0.28, 1.89]**	
Total events	31		45				

Heterogeneity: Tau² = 0.50; Chi² = 6.83, df = 3 (P = 0.08); I² = 56%

Test for overall effect: Z = 0.66 (P = 0.51)

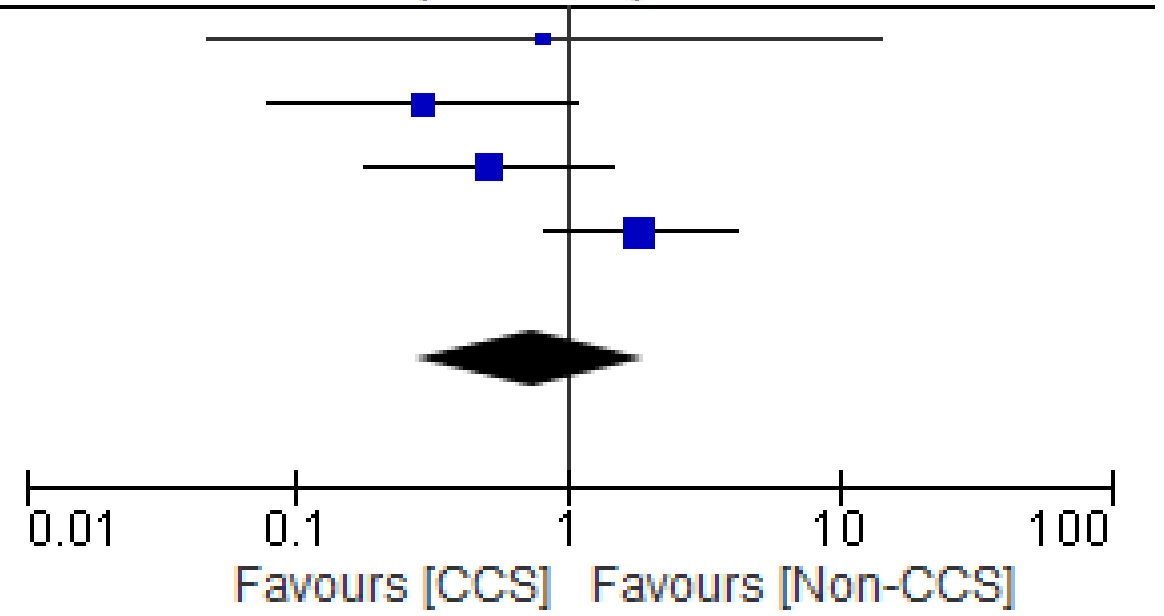

Hydrocortisone - CCS vs Non-CCS: 10.Reintervention

Study or Subgroup	Experimental (CCS) Events	Experimental (CCS) Total	Control (Non-CCS) Events	Control (Non-CCS) Total	Weight	Odds Ratio M-H, Random, 95% CI
Antila 2019	0	17	1	14	35.6%	0.26 [0.01, 6.82]
Kant 2024	1	35	2	70	64.4%	1.00 [0.09, 11.42]
**Total (95% CI)**		**52**		**84**	**100.0%**	**0.62 [0.09, 4.36]**
Total events	1		3			

Heterogeneity: Tau² = 0.00; Chi² = 0.43, df = 1 (P = 0.51); I² = 0%
Test for overall effect: Z = 0.48 (P = 0.63)

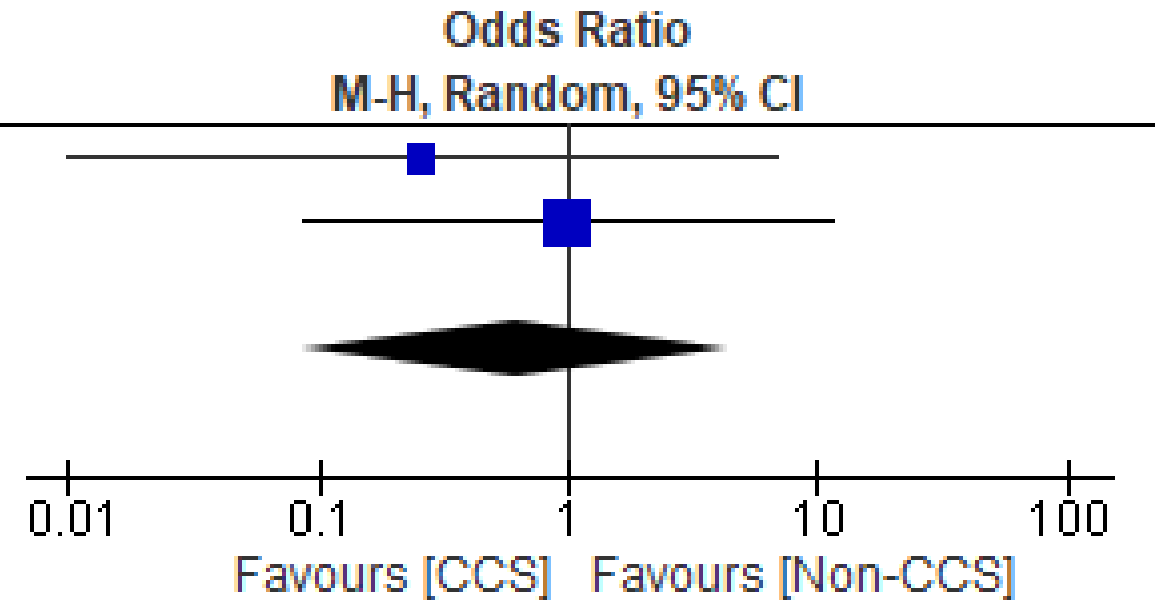

Hydrocortisone - CCS vs Non-CCS: 11.Readmission

	Experimental (CCS)		Control (Non-CCS)			Odds Ratio
**Study or Subgroup**	**Events**	**Total**	**Events**	**Total**	**Weight**	**M-H, Random, 95% CI**
Antila 2019	3	17	3	14	18.6%	0.79 [0.13, 4.68]
Kant 2024	2	35	4	70	19.4%	1.00 [0.17, 5.74]
Laaninen 2016	0	28	4	34	6.7%	0.12 [0.01, 2.31]
Tarvainen 2020	10	63	7	63	55.2%	1.51 [0.54, 4.25]
**Total (95% CI)**		**143**		**181**	**100.0%**	**1.04 [0.48, 2.25]**
Total events	15		18			

Heterogeneity: $Tau^2 = 0.00$; $Chi^2 = 2.73$, df = 3 (P = 0.43); $I^2 = 0\%$

Test for overall effect: Z = 0.10 (P = 0.92)

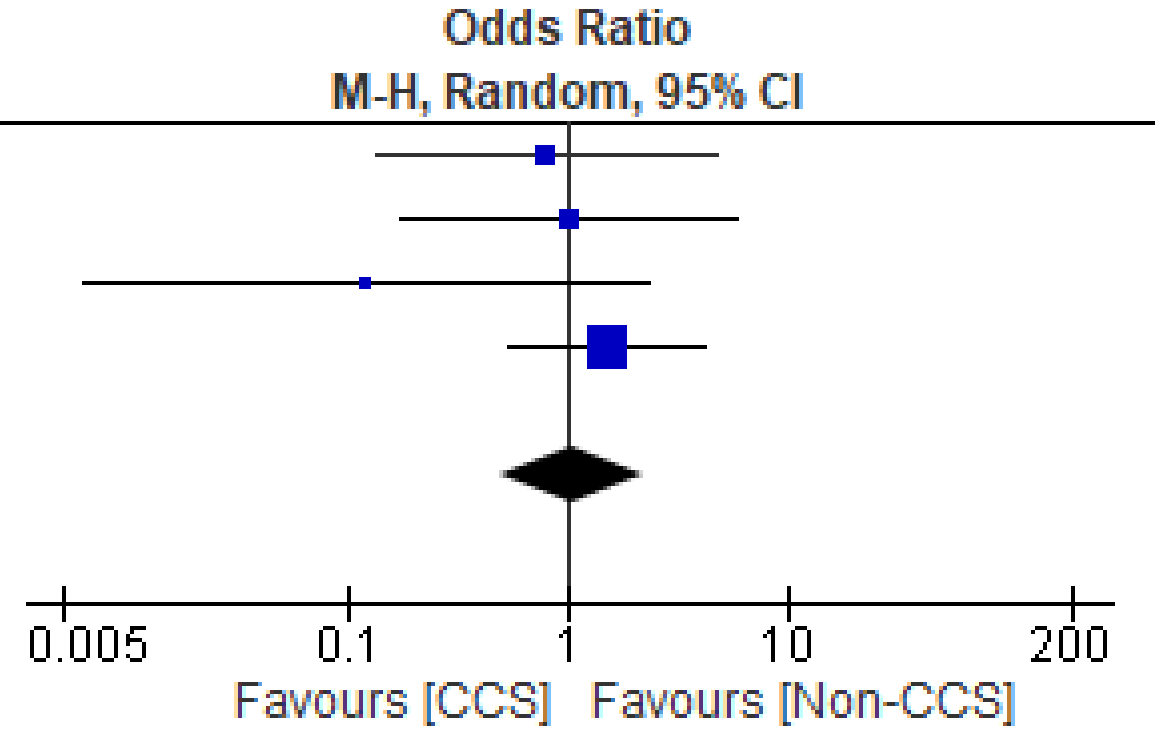

Hydrocortisone - CCS vs Non-CCS: 12.LOS

Study or Subgroup	Experimental (CCS) Mean	SD	Total	Control (Non-CCS) Mean	SD	Total	Weight	Mean Difference IV, Random, 95% CI
Antila 2019	8	5.2	17	7	8	14	13.6%	1.00 [-3.87, 5.87]
Kant 2024	10	4.4	35	11	4.4	70	37.0%	-1.00 [-2.79, 0.79]
Laaninen 2016	9	12.2	28	11	12.2	34	9.6%	-2.00 [-8.10, 4.10]
Tarvainen 2020	10	4.4	63	8	4.4	63	39.8%	2.00 [0.46, 3.54]
**Total (95% CI)**			**143**			**181**	**100.0%**	**0.37 [-1.73, 2.47]**

Heterogeneity: Tau² = 2.26; Chi² = 6.99, df = 3 (P = 0.07); I² = 57%
Test for overall effect: Z = 0.35 (P = 0.73)

Mean Difference
IV, Random, 95% CI

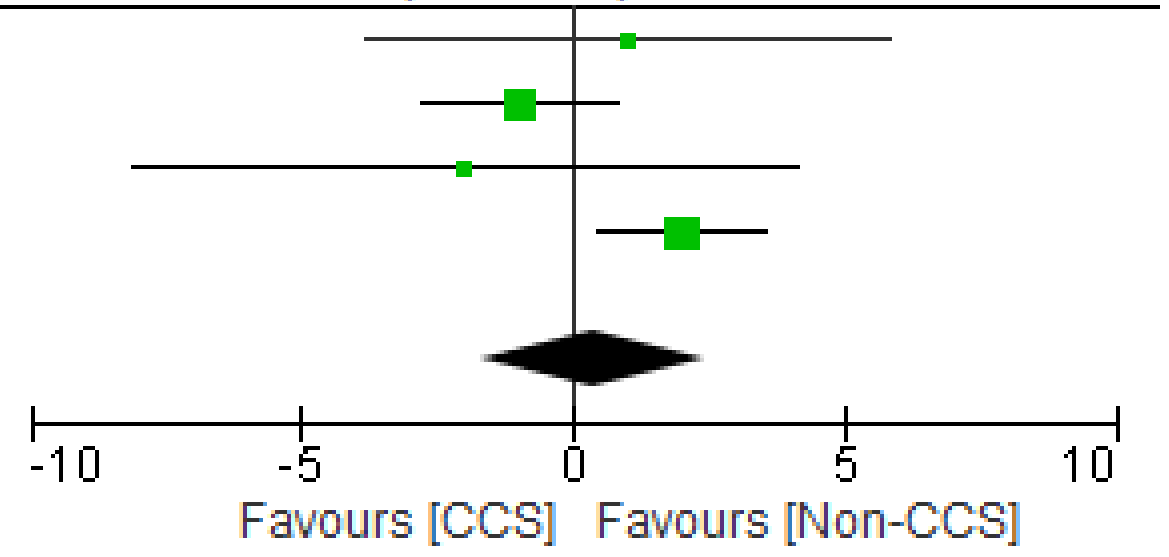

Dexamethasone - CCS vs Non-CCS: 1.Mortality

	Experimental (CCS)		Control (Non-CCS)			Odds Ratio	Odds Ratio
**Study or Subgroup**	**Events**	**Total**	**Events**	**Total**	**Weight**	**M-H, Random, 95% CI**	**M-H, Random, 95% CI**
Chen 2024	3	134	3	131	47.2%	0.98 [0.19, 4.93]	
Sandini 2018	2	117	10	562	52.8%	0.96 [0.21, 4.44]	
**Total (95% CI)**		**251**		**693**	**100.0%**	**0.97 [0.32, 2.94]**	
Total events	5		13				

Heterogeneity: Tau² = 0.00; Chi² = 0.00, df = 1 (P = 0.99); I² = 0%

Test for overall effect: Z = 0.06 (P = 0.95)

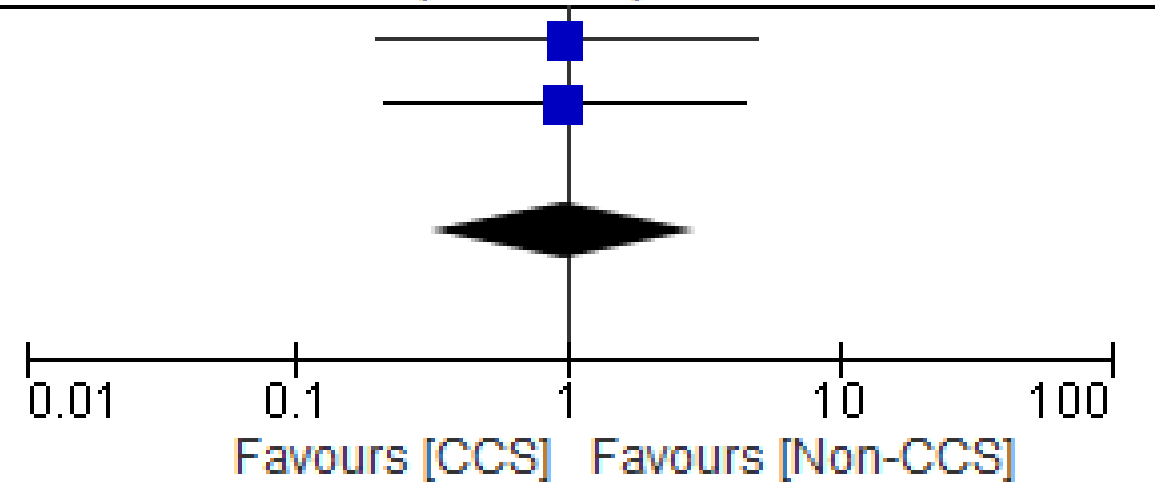

Dexamethasone - CCS vs Non-CCS: 2.Morbidity mayor

Study or Subgroup	Experimental (CCS) Events	Experimental (CCS) Total	Control (Non-CCS) Events	Control (Non-CCS) Total	Weight	Odds Ratio M-H, Random, 95% CI	Odds Ratio M-H, Random, 95% CI
Chen 2024	17	134	21	131	22.2%	0.76 [0.38, 1.52]	
Newhook 2021	47	223	29	150	39.5%	1.11 [0.66, 1.87]	
Sandini 2018	20	117	104	562	38.2%	0.91 [0.54, 1.54]	
**Total (95% CI)**		**474**		**843**	**100.0%**	**0.95 [0.68, 1.31]**	
Total events	84		154				

Heterogeneity: Tau² = 0.00; Chi² = 0.79, df = 2 (P = 0.67); I² = 0%

Test for overall effect: Z = 0.33 (P = 0.74)

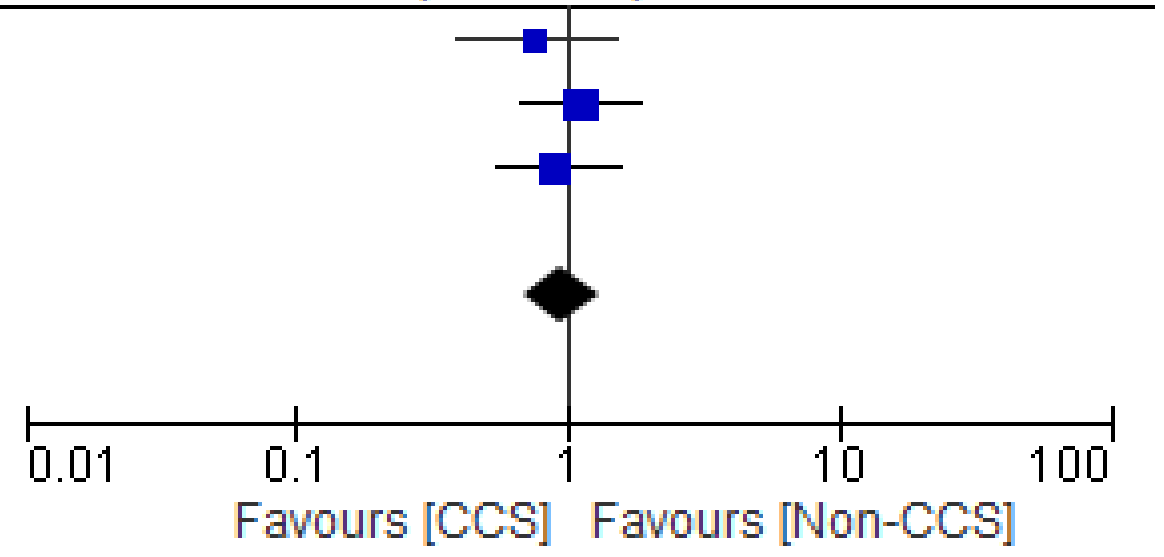

Dexamethasone - CCS vs Non-CCS: 3.Morbidity minor

Study or Subgroup	Experimental (CCS) Events	Experimental (CCS) Total	Control (Non-CCS) Events	Control (Non-CCS) Total	Weight	Odds Ratio M-H, Random, 95% CI
Chen 2024	17	134	21	131	25.4%	0.76 [0.38, 1.52]
Sandini 2018	49	117	249	562	74.6%	0.91 [0.61, 1.36]
**Total (95% CI)**		**251**		**693**	**100.0%**	**0.87 [0.61, 1.23]**
Total events	66		270			

Heterogeneity: Tau² = 0.00; Chi² = 0.18, df = 1 (P = 0.67); I² = 0%

Test for overall effect: Z = 0.81 (P = 0.42)

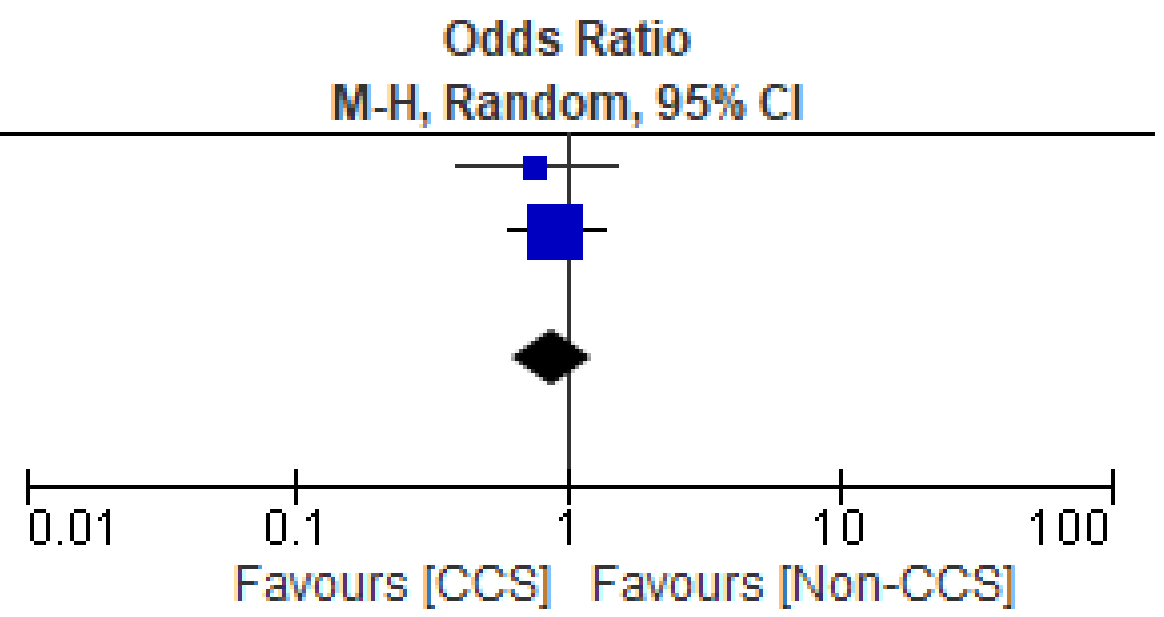

Dexamethasone - CCS vs Non-CCS: 4.Morbidity global

Study or Subgroup	Experimental (CCS) Events	Experimental (CCS) Total	Control (Non-CCS) Events	Control (Non-CCS) Total	Weight	Odds Ratio M-H, Random, 95% CI
Chen 2024	17	134	21	131	17.6%	0.76 [0.38, 1.52]
Newhook 2021	47	223	29	150	31.4%	1.11 [0.66, 1.87]
Sandini 2018	69	117	353	562	51.0%	0.85 [0.57, 1.28]
**Total (95% CI)**		**474**		**843**	**100.0%**	**0.91 [0.68, 1.21]**
Total events	133		403			

Heterogeneity: Tau² = 0.00; Chi² = 0.95, df = 2 (P = 0.62); I² = 0%

Test for overall effect: Z = 0.65 (P = 0.51)

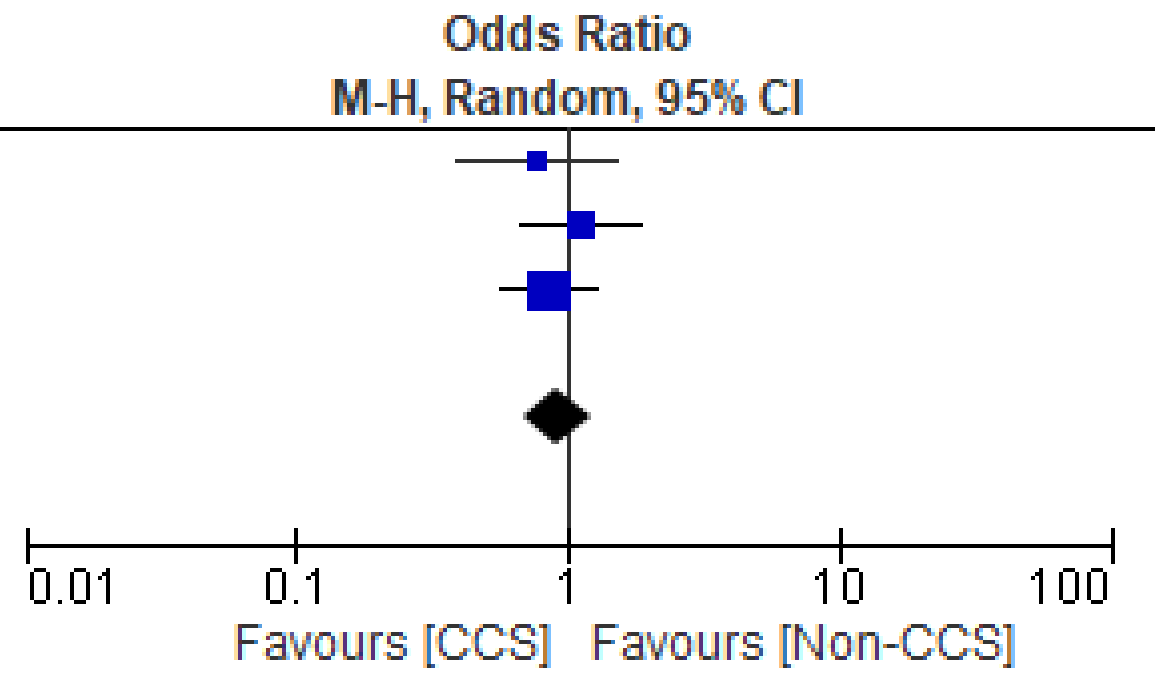

Dexamethasone - CCS vs Non-CCS: 5.CR-POPF

	Experimental (CCS)		Control (Non-CCS)			Odds Ratio
Study or Subgroup	Events	Total	Events	Total	Weight	M-H, Random, 95% CI
Chen 2024	34	134	41	131	56.5%	0.75 [0.44, 1.28]
Newhook 2021	14	223	10	150	23.1%	0.94 [0.41, 2.17]
Sandini 2018	6	117	34	562	20.4%	0.84 [0.34, 2.05]
**Total (95% CI)**		**474**		**843**	**100.0%**	**0.81 [0.54, 1.21]**
Total events	54		85			

Heterogeneity: Tau² = 0.00; Chi² = 0.21, df = 2 (P = 0.90); I² = 0%

Test for overall effect: Z = 1.05 (P = 0.29)

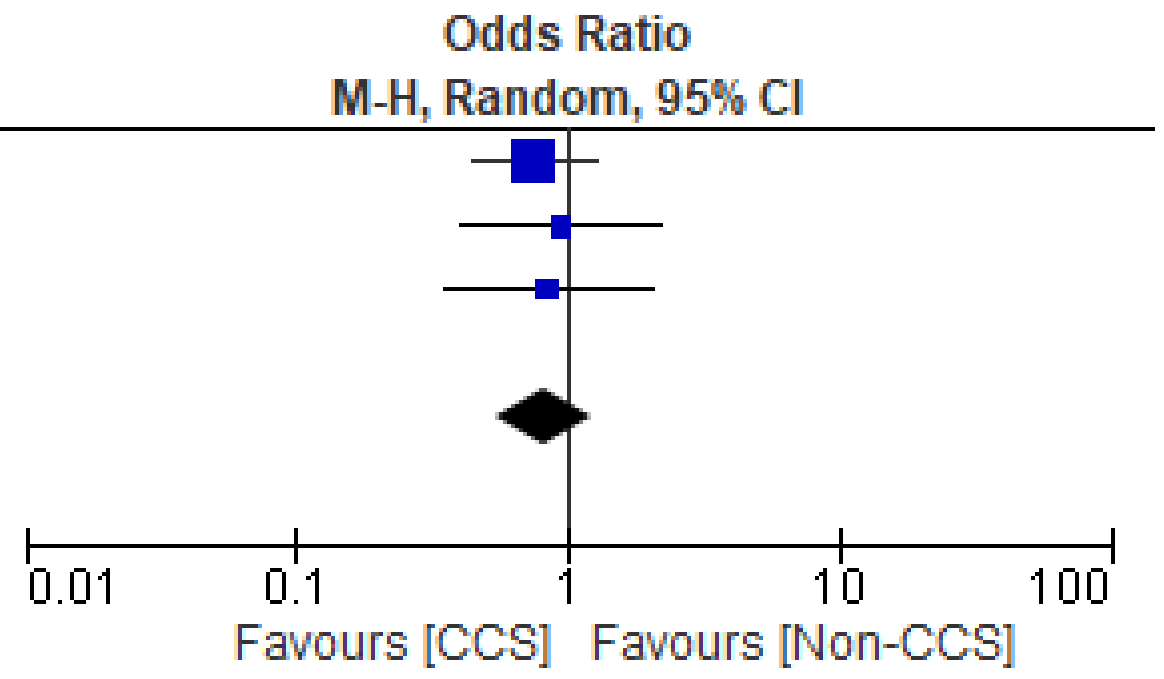

Dexamethasone - CCS vs Non-CCS: 6.POPF (biochemical leak and CR-POPF)

Study or Subgroup	Experimental (CCS) Events	Experimental (CCS) Total	Control (Non-CCS) Events	Control (Non-CCS) Total	Weight	Odds Ratio M-H, Random, 95% CI
Chen 2024	34	134	41	131	56.5%	0.75 [0.44, 1.28]
Newhook 2021	14	223	10	150	23.1%	0.94 [0.41, 2.17]
Sandini 2018	6	117	34	562	20.4%	0.84 [0.34, 2.05]
**Total (95% CI)**		**474**		**843**	**100.0%**	**0.81 [0.54, 1.21]**
Total events	54		85			

Heterogeneity: $Tau^2 = 0.00$; $Chi^2 = 0.21$, $df = 2$ ($P = 0.90$); $I^2 = 0\%$

Test for overall effect: $Z = 1.05$ ($P = 0.29$)

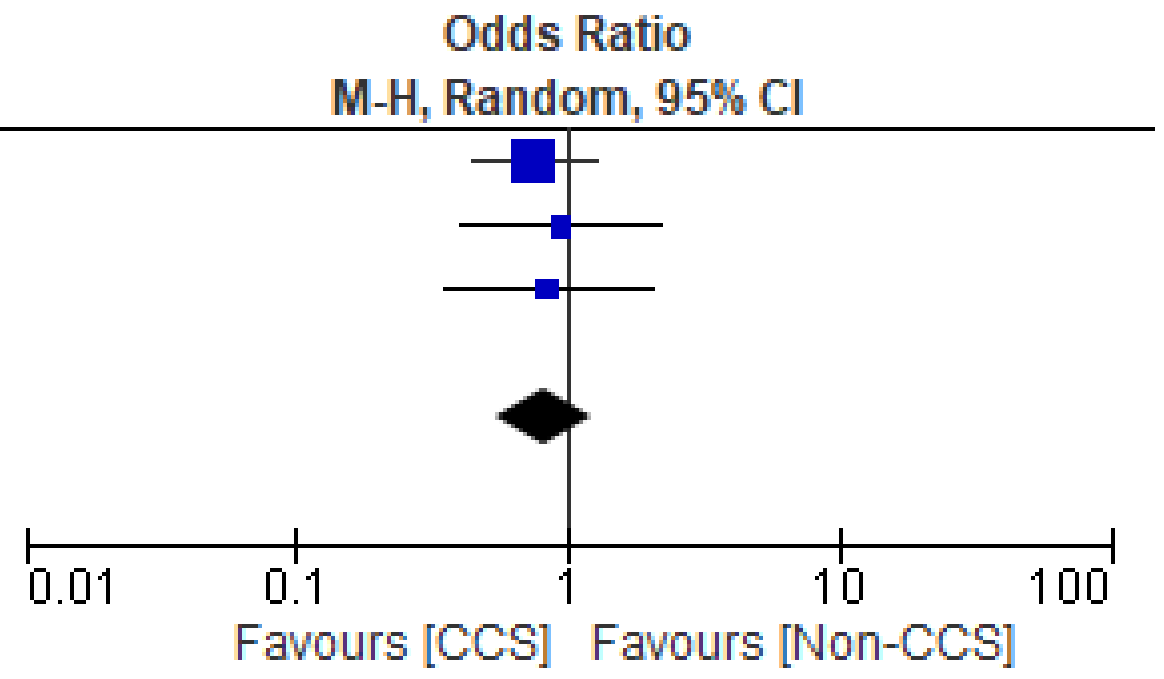

Dexamethasone - CCS vs Non-CCS: 7.Bile Leak

	Experimental (CCS)		Control (Non-CCS)			Odds Ratio
**Study or Subgroup**	**Events**	**Total**	**Events**	**Total**	**Weight**	**M-H, Random, 95% CI**
Chen 2024	6	134	6	131	43.3%	0.98 [0.31, 3.11]
Sandini 2018	5	117	18	562	56.7%	1.35 [0.49, 3.71]
**Total (95% CI)**		**251**		**693**	**100.0%**	**1.17 [0.55, 2.51]**
Total events	11		24			

Heterogeneity: Tau² = 0.00; Chi² = 0.17, df = 1 (P = 0.68); I² = 0%

Test for overall effect: Z = 0.41 (P = 0.68)

Odds Ratio
M-H, Random, 95% CI

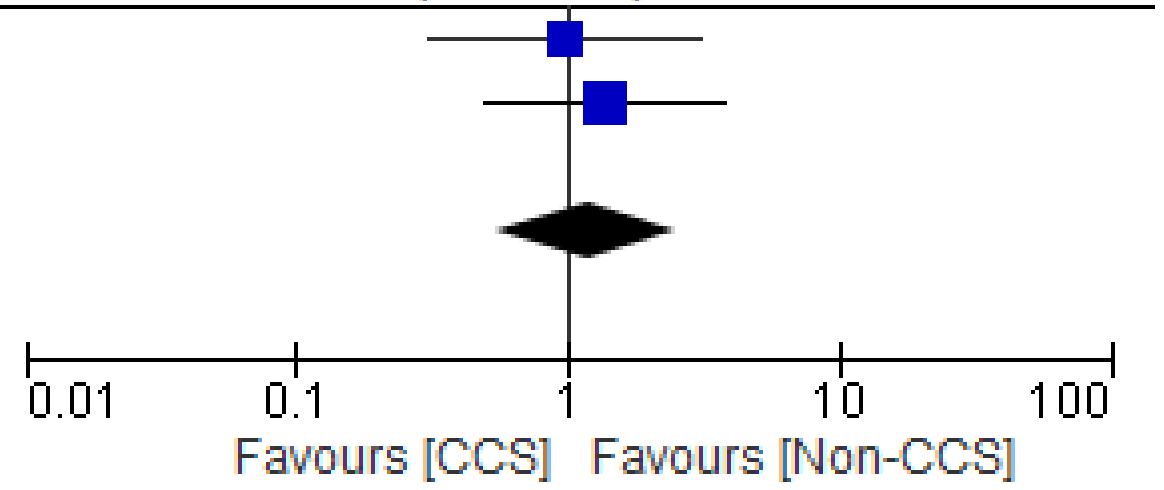

Dexamethasone - CCS vs Non-CCS: 8.SSI

Study or Subgroup	Experimental (CCS) Events	Experimental (CCS) Total	Control (Non-CCS) Events	Control (Non-CCS) Total	Weight	Odds Ratio M-H, Random, 95% CI
Chen 2024	13	134	22	131	26.9%	0.53 [0.26, 1.11]
Newhook 2021	64	223	37	150	37.1%	1.23 [0.77, 1.97]
Sandini 2018	22	117	160	562	36.0%	0.58 [0.35, 0.96]
**Total (95% CI)**		**474**		**843**	**100.0%**	**0.75 [0.43, 1.30]**
Total events	99		219			

Heterogeneity: Tau² = 0.16; Chi² = 5.95, df = 2 (P = 0.05); I² = 66%
Test for overall effect: Z = 1.02 (P = 0.31)

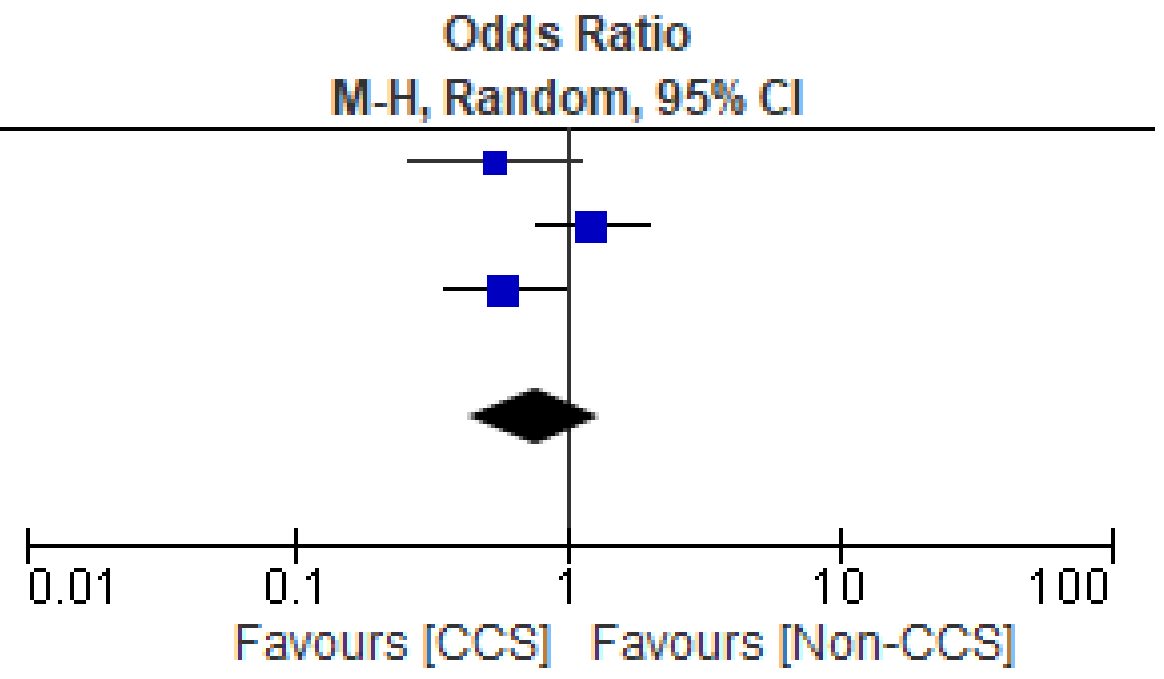

Dexamethasone - CCS vs Non-CCS: 9.DGE

Study or Subgroup	Experimental (CCS) Events	Experimental (CCS) Total	Control (Non-CCS) Events	Control (Non-CCS) Total	Weight	Odds Ratio M-H, Random, 95% CI
Chen 2024	13	134	17	131	18.2%	0.72 [0.33, 1.55]
Newhook 2021	60	223	33	150	45.2%	1.31 [0.80, 2.12]
Sandini 2018	19	117	90	562	36.6%	1.02 [0.59, 1.75]
**Total (95% CI)**		**474**		**843**	**100.0%**	**1.07 [0.77, 1.48]**
Total events	92		140			

Heterogeneity: Tau² = 0.00; Chi² = 1.70, df = 2 (P = 0.43); I² = 0%

Test for overall effect: Z = 0.40 (P = 0.69)

Odds Ratio
M-H, Random, 95% CI

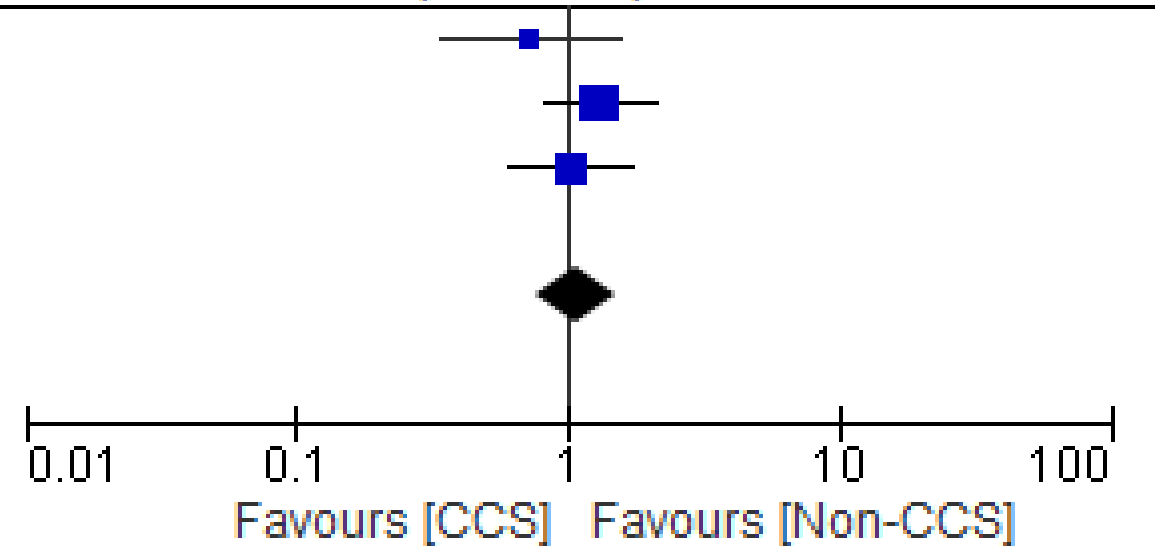

Dexamethasone - CCS vs Non-CCS: 10.Reintervention

Study or Subgroup	Experimental (CCS) Events	Experimental (CCS) Total	Control (Non-CCS) Events	Control (Non-CCS) Total	Weight	Odds Ratio M-H, Random, 95% CI
Chen 2024	1	134	7	131	40.8%	0.13 [0.02, 1.10]
Sandini 2018	3	117	15	562	59.2%	0.96 [0.27, 3.37]
**Total (95% CI)**		**251**		**693**	**100.0%**	**0.43 [0.06, 2.99]**
Total events	4		22			

Heterogeneity: Tau² = 1.25; Chi² = 2.59, df = 1 (P = 0.11); I² = 61%
Test for overall effect: Z = 0.85 (P = 0.39)

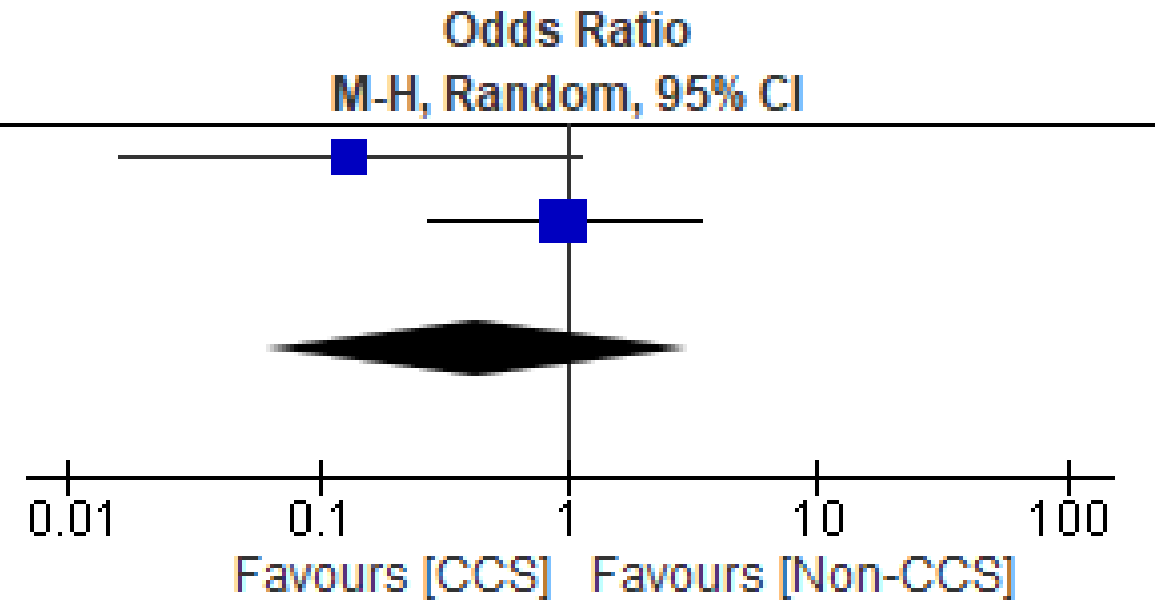

Dexamethasone - CCS vs Non-CCS: 11.Readmission

Study or Subgroup	Experimental (CCS) Events	Experimental (CCS) Total	Control (Non-CCS) Events	Control (Non-CCS) Total	Weight	Odds Ratio M-H, Random, 95% CI
Sandini 2018	19	117	104	562	100.0%	0.85 [0.50, 1.46]
**Total (95% CI)**		**117**		**562**	**100.0%**	**0.85 [0.50, 1.46]**
Total events	19		104			

Heterogeneity: Not applicable
Test for overall effect: Z = 0.58 (P = 0.56)

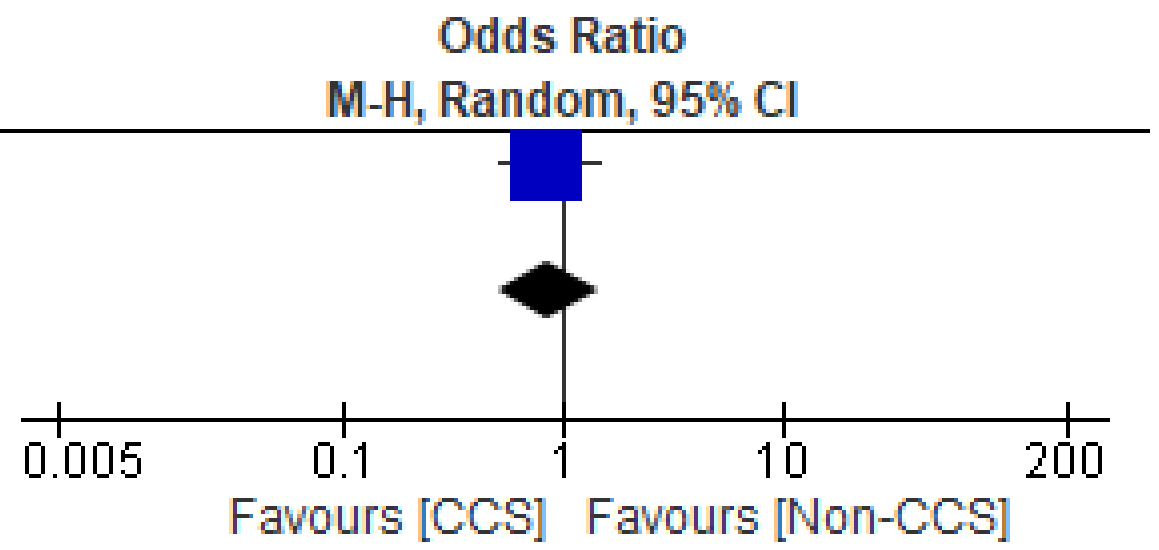

Dexamethasone - CCS vs Non-CCS: 12.LOS

	Experimental (CCS)			Control (Non-CCS)				Mean Difference	Mean Difference
**Study or Subgroup**	**Mean**	**SD**	**Total**	**Mean**	**SD**	**Total**	**Weight**	**IV, Random, 95% CI**	**IV, Random, 95% CI**
Chen 2024	16	8.3	134	19	15	131	40.0%	-3.00 [-5.93, -0.07]	
Newhook 2021	8	6.2	223	8	6.2	150	60.0%	0.00 [-1.28, 1.28]	
**Total (95% CI)**			**357**			**281**	**100.0%**	**-1.20 [-4.08, 1.68]**	

Heterogeneity: $Tau^2 = 3.17$; $Chi^2 = 3.38$, df = 1 (P = 0.07); $I^2 = 70\%$

Test for overall effect: Z = 0.82 (P = 0.41)

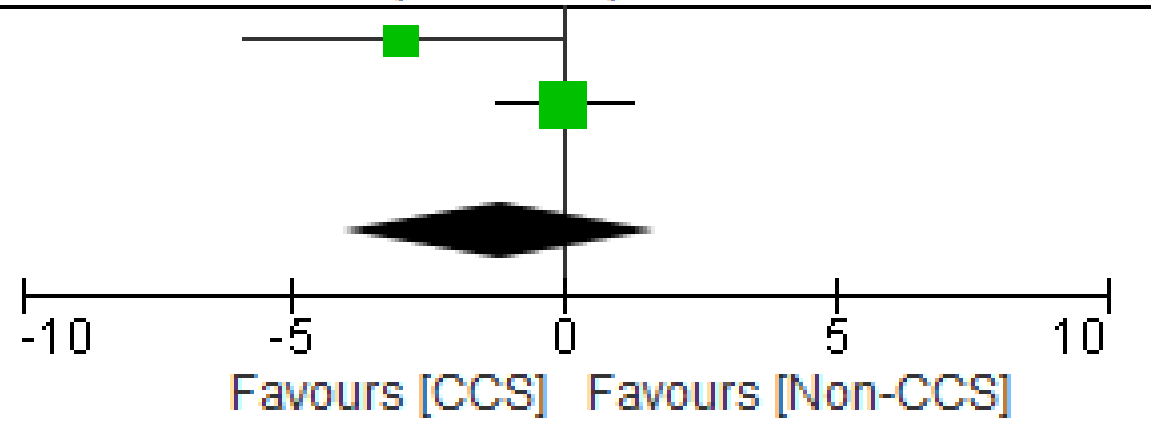

Supplement: Supplementary file 2 [file 13304_2026_2750_MOESM2_ESM.pdf]
