# Supplementary material for: Perioperative corticosteroids and pancreatic surgery outcomes: a systematic review and meta-analysis combining human expertise and AI support (ChatGPT)
Source: Updates Surg. 2026 Jul 8;78(6):2549–59. doi: 10.1007/s13304-026-02750-3 (PMC13737091; doi:10.1007/s13304-026-02750-3)
Supplement: Supplementary file 3 [file 13304_2026_2750_MOESM3_ESM.docx]

Supplementary Table 1: Risk of bias assessment of randomised clinical trials according to the Risk of Bias-2 (RoB-2) tool

| **Study** | **D1: Randomization process** | **D2: Deviations from intended interventions** | **D3: Missing outcome data** | **D4: Measurement of the outcomes** | **D5: Selection of the reported result** | **Overall RoB** |
| --- | --- | --- | --- | --- | --- | --- |
| Antila 2019 | Some concerns | Some concerns | Some concerns | Low | Low | Some concerns |
| Chen 2024 | Low | Low | Low | Low | Low | Low |
| Kant 2024 | Low | Low | Low | Low | Low | Low |
| Laaninen 2016 | Low | Low | Low | Low | Some concerns | Some concerns |
| Tarvainen 2020 | Low | Low | Low | Low | Low | Low |

Supplementary Table 2: Risk of Bias assessment of observational studies according to the ROBINS-I tool

| **Study** | **Confounding** | **Selection of Participants** | **Classification of Interventions** | **Deviations from Interventions** | **Missing Data** | **Measurement of Outcomes** | **Selection of Reported Results** | **Overall Risk of Bias** |
| --- | --- | --- | --- | --- | --- | --- | --- | --- |
| Call 2015 | Serious | Moderate | Low | Low | Moderate | Moderate | Moderate | Serious |
| Newhook 2021 | Moderate | Low | Low | Low | Low | Low | Moderate | Moderate |
| Sandini 2018 | Serious | Moderate | Low | Low | Moderate | Moderate | Moderate | Serious |
| Zhang 2021 | Moderate | Low | Low | Low | Low | Low | Low | Moderate |

Supplementary Table 3: Summary table of CCS vs Non-CCS Outcomes

| **CCS vs Non-CCS Outcomes: data on hydrocortisone** | | | | | |
| --- | --- | --- | --- | --- | --- |
| **Outcomes** | **Studies** | **Patients (CCS vs non-CCS)** | **OR / MD (95% CI)** | **p-value** | **I²** |
| Mortality | 3 | 143 vs 181 | 1.35 (0.19–9.37) | 0.76 | 0% |
| Major morbidity | 4 | 143 vs 181 | 0.76 (0.26–2.27) | 0.63 | 56% |
| Minor morbidity | 3 | 115 vs 147 | 0.64 (0.28–1.45) | 0.28 | 50% |
| Global morbidity | 4 | 143 vs 181 | 0.57 (0.19–1.68) | 0.31 | 67% |
| CR-POPF | 4 | 143 vs 181 | 0.54 (0.21–1.36) | 0.19 | 42% |
| POPF | 4 | 143 vs 181 | 0.76 (0.39–1.50) | 0.43 | 34% |
| Bile leak | 1 | 28 vs 34 | 0.21 (0.02–1.96) | 0.17 | – |
| SSI | 3 | 80 vs 118 | 0.54 (0.25–1.15) | 0.11 | 0% |
| DGE | 4 | 143 vs 181 | 0.72 (0.28–1.89) | 0.51 | 56% |
| Reintervention | 2 | 52 vs 84 | 0.62 (0.09–4.36) | 0.63 | 0% |
| Readmission | 4 | 143 vs 181 | 1.04 (0.48–2.25) | 0.92 | 0% |
| Length of stay (days) | 4 | 143 vs 181 | 0.37 (–1.73–2.47) | 0.73 | 57% |
| **CCS vs Non-CCS Outcomes: data on dexamethasone** | | | | | |
| Mortality | 2 | 251 vs 693 | 0.97 (0.32–2.94) | 0.95 | 0% |
| Major morbidity | 3 | 474 vs 843 | 0.95 (0.68–1.31) | 0.74 | 0% |
| Minor morbidity | 2 | 251 vs 693 | 0.87 (0.61–1.23) | 0.42 | 0% |
| Global morbidity | 3 | 474 vs 843 | 0.91 (0.68–1.21) | 0.51 | 0% |
| CR-POPF | 3 | 474 vs 843 | 0.81 (0.54–1.21) | 0.29 | 0% |
| POPF | 3 | 474 vs 843 | 0.81 (0.54–1.21) | 0.29 | 0% |
| Bile leak | 2 | 251 vs 693 | 1.17 (0.55–2.51) | 0.68 | 0% |
| SSI | 3 | 474 vs 843 | 0.75 (0.43–1.30) | 0.31 | 66% |
| DGE | 3 | 474 vs 843 | 1.07 (0.77–1.48) | 0.69 | 0% |
| Reintervention | 2 | 251 vs 693 | 0.43 (0.06–2.99) | 0.39 | 61% |
| Readmission | 1 | 117 vs 562 | 0.85 (0.50–1.46) | 0.56 | NA |
| Length of stay (days) | 2 | 357 vs 281 | –1.20 (–4.08–1.68) | 0.41 | 70% |

Supplementary Table 4. Correlation of matched outcomes between human and ChatGPT generated dataset. Pearson’s correlation coefficients (r) and corresponding p-values for all matched variables across studies. Intervention arms are reported as Intervention 1 and Intervention 2. The analysis shows strong agreement (r = 0.97–1.00, all p < 0.001).

| **Variable** | **Pearson r** | **p-value** |
| --- | --- | --- |
| Sample size (n) | 0.989836 | <0.001 |
| Intervention 1 number of patients (n) | 0.969969 | <0.001 |
| Intervention 2 number of patients (n) | 0.993938 | <0.001 |
| Intervention 1 mortality (n) | 1 | <0.001 |
| Intervention 2 mortality (n) | 0.990828 | <0.001 |
| Intervention 1 CRPF (n) | 1 | <0.001 |
| Intervention 2 CRPF (n) | 0.997095 | <0.001 |
| Intervention 1 PF (n) | 0.975258 | 0.142 |
| Intervention 2 PF (n) | 0.997827 | 0.042 |
| Intervention 1 mayor complications (n) | 1 | <0.001 |
| Intervention 2 mayor complications (n) | 0.999949 | <0.001 |
| Intervention 1 PPH (n) | 0.97828 | 0.004 |
| Intervention 2 PPH (n) | 0.981814 | 0.003 |
| Intervention 1 DGE (n) | 1 | <0.001 |
| Intervention 2 DGE (n) | 0.999926 | <0.001 |
| Intervention 1 SSI (n) | 1 | <0.001 |
| Intervention 2 SSI (n) | 0.999979 | <0.001 |
| Intervention 1 Reintervention (n) | 1 | <0.001 |
| Intervention 2 Reintervention (n) | 0.991518 | 0.008 |
| Intervention 1 readmission (n) | 1 | <0.001 |
| Intervention 2 readmission (n) | 1 | <0.001 |

Supplementary Table 5: Substantial discrepancies (>5% relative difference) between human and ChatGPT generated dataset

| **Study ID** | **Variable** | **Dataset 1** | **Dataset 2** | **Absolute Difference** | **Relative Difference (%)** |
| --- | --- | --- | --- | --- | --- |
| Antila 2019 | Intervent1 PF (n) | 1 | 7 | 6 | 600 |
| Antila 2019 | Intervent2 PF (n) | 6 | 7 | 1 | 16.67 |
| Chen 2024 | Intervent2 mortality (n) | 3 | 2 | -1 | 33.33 |
| Kant 2024 | Intervent2 mortality (n) | 1 | 0 | -1 | 100 |
| Kant 2024 | Intervent2 CRPF (n) | 9 | 6 | -3 | 33.33 |
| Kant 2024 | Intervent2 mayor complications (n) | 3 | 2 | -1 | 33.33 |
| Kant 2024 | Intervent2 PPH (n) | 5 | 4 | -1 | 20 |
| Kant 2024 | Intervent2 DGE (n) | 8 | 9 | 1 | 12.5 |
| Kant 2024 | Intervent2 SSI (n) | 9 | 10 | 1 | 11.11 |
| Kant 2024 | Intervent2 Reintervention (n) | 2 | 0 | -2 | 100 |
| Laaninen 2016 | Intervent1 PF (n) | 6 | 3 | -3 | 50 |
| Laaninen 2016 | Intervent2 PF (n) | 10 | 9 | -1 | 10 |
| Laaninen 2016 | Intervent1 PPH (n) | 4 | 3 | -1 | 25 |
| Laaninen 2016 | Intervent2 PPH (n) | 8 | 5 | -3 | 37.5 |
| Zhang 2021 | Sample size (n) | 128 | 215 | 87 | 67.97 |
| Zhang 2021 | Intervent1 number of patients (n) | 64 | 112 | 48 | 75 |
| Zhang 2021 | Intervent2 number of patients (n) | 64 | 103 | 39 | 60.94 |
